# A patient-safety and professional perspective on non-conveyance in ambulance care: a systematic review

**DOI:** 10.1186/s13049-017-0409-6

**Published:** 2017-07-17

**Authors:** Remco H.A. Ebben, Lilian C.M. Vloet, Renate F. Speijers, Nico W. Tönjes, Jorik Loef, Thomas Pelgrim, Margreet Hoogeveen, Sivera A.A. Berben

**Affiliations:** 1 Research Department of Emergency and Critical Care, HAN University of Applied Sciences, Faculty of Health and Social Studies, PO Box 6960, 6503 GL Nijmegen, The Netherlands; 2 Radboud University Medical Center, Radboud Institute for Health Sciences, IQ healthcare, Nijmegen, The Netherlands; 3 Ambulance Service Gelderland-Zuid, Nijmegen, The Netherlands; 4 Ambulance Service IJsselland, Zwolle, The Netherlands; 5 Ambulance Academy, Harderwijk, The Netherlands; 6 Dutch National Sector Organisation for Ambulance Care, Zwolle, The Netherlands; 7 Radboud University Medical Center, Eastern Regional Emergency Healthcare Network, Nijmegen, The Netherlands

**Keywords:** Emergency medical services [MeSH], Patient safety [MeSH], Clinical competence [MeSH], Non-conveyance

## Abstract

**Background:**

This systematic review aimed to describe non-conveyance in ambulance care from patient-safety and ambulance professional perspectives. The review specifically focussed at describing (1) ambulance non-conveyance rates, (2) characteristics of non-conveyed patients, (3) follow-up care after non-conveyance, (4) existing guidelines or protocols, and (5) influencing factors during the non-conveyance decision making process.

**Methods:**

We systematically searched MEDLINE, PubMed, CINAHL, EMBASE, and reference lists of included articles, in June 2016. We included all types of peer-reviewed designs on the five topics. Couples of two independent reviewers performed the selection process, the quality assessment, and data extraction.

**Results:**

We included 67 studies with low to moderate quality. Non-conveyance rates for general patient populations ranged from 3.7%–93.7%. Non-conveyed patients have a variety of initial complaints, common initial complaints are related to trauma and neurology. Furthermore, vulnerable patients groups as children and elderly are more represented in the non-conveyance population. Within 24 h–48 h after non-conveyance, 2.5%–6.1% of the patients have EMS representations, and 4.6–19.0% present themselves at the ED. Mortality rates vary from 0.2%–3.5% after 24 h, up to 0.3%–6.1% after 72 h. Criteria to guide non-conveyance decisions are vital signs, ingestion of drugs/alcohol, and level of consciousness. A limited amount of non-conveyance guidelines or protocols is available for general and specific patient populations. Factors influencing the non-conveyance decision are related to the professional (competencies, experience, intuition), the patient (health status, refusal, wishes and best interest), the healthcare system (access to general practitioner/other healthcare facilities/patient information), and supportive tools (online medical control, high risk card).

**Conclusions:**

Non-conveyance rates for general and specific patient populations vary. Patients in the non-conveyance population present themselves with a variety of initial complaints and conditions, common initial complaints or conditions are related to trauma and neurology. After non-conveyance, a proportion of patients re-enters the emergency healthcare system within 2 days. For ambulance professionals the non-conveyance decision-making process is complex and multifactorial. Competencies needed to perform non-conveyance are marginally described, and there is a limited amount of supportive tools is available for general and specific non-conveyance populations. This may compromise patient-safety.

**Electronic supplementary material:**

The online version of this article (doi:10.1186/s13049-017-0409-6) contains supplementary material, which is available to authorized users.

## Background

The past decades, ambulance care has evolved from a health care facility that conveys patients to the hospital, into emergency medical services (EMS) that provide advanced out-of-hospital care for (non-) life-threatening conditions [1, 2]. At the same time, the utilization of ambulance care has increased throughout the developed world, with various underlying reasons such as ageing of the population, changes in social support, accessibility and costs [3]. Together, these developments put a growing demand on ambulance systems and ambulance capacity, the emergency departments (ED) and the wider healthcare system, and this may compromise patient safety, healthcare quality, and access [3]. In addition to this growing demand, frequent overcrowding of the ED occurs [4, 5].

The ambulance process is situated within this context. This process often results in patient conveyance to an ED or other healthcare facility, but ambulance care can also result in patients not being conveyed. The NHS Litigation Authority (2012) defines conveyance as “the transfer of patients, medical and clinical personnel, equipment and associated records, as appropriate including from one healthcare facility to another as well as the initial journey from the scene.” [6]. Non-conveyance is defined as “an ambulance deployment as appropriate, where the patient after examination and/or treatment on-scene does not require conveyance with medical personnel and equipment to the healthcare facility” [7]. Non-conveyed patients can be treated and ‘discharged’ on-scene, or may be referred to other (primary) healthcare facilities such as the general practitioner. According to the literature, non-conveyance can be divided in two categories: the patient-initiated refusal and the ambulance professional decision [8]. Often, non-conveyance is a combination of these two categories.

Non-conveyance rates of patients who received on-scene emergency care from an ambulance emergency crew, have been reported up to 30% [9, 10]. On the other hand, it has been estimated that 11%–61% of the conveyances is medically not necessary [11]. Factors influencing these non-conveyance rates are patients with low-acuity problems or primary care problems who call an ambulance [12, 13], accuracy of triage systems at the EMS dispatch centre [14], and professional competencies [15].

The priority to conduct research on non-conveyance is reflected on the Dutch National Pre-hospital Research Agenda for EMS 2014–2018 [16]. From patient-safety and professional perspective, little is known about non-conveyance. Insight into characteristics and outcomes of the non-conveyance patients is lacking. Furthermore, it is unknown how often non-conveyance exactly occurs, which complaints non-conveyed patients have, what care is provided after non-conveyance, and how often these patients have adverse events. Conversely from the professional perspective, little is known about the on-scene non-conveyance decision-making process. As ambulance care has become a more complex environment, ambulance professionals are faced with decision-making over multiple care options as conveyance to an emergency department, or another non-emergency service, treat-and-release or referral to another healthcare professional [17]. Literature described that this decision-making process requires adequate competencies, skills and clinical reasoning of ambulance professionals [18], although ambulance professionals curricula include a little on conveyance decision making [19]. Also, few ambulance services developed non-conveyance protocols and policies [20]. However, the question is whether the literature describes guidelines, protocols or triage criteria to support the ambulance professionals in the decision making process for non-conveyance, how competent are they to decide and apply for non-conveyance, and how are they influenced during the decision making process for non-conveyance? These aspects of patient safety and ambulance professional perspectives related to non-conveyance in ambulance EMS have not yet been synthesized in an overview.

### Aim

The aim of this systematic review is twofold. The first aim is safety orientated, as we want to describe non-conveyance rates, characteristics of patients, and follow-up care after non-conveyance. The second aim is formulated from the perspective of the ambulance professional, as we want to describe available guidelines or protocols and triage criteria, competencies needed by ambulance professionals to make appropriate (non-) conveyance decisions, and also to describe which factors influence ambulance professionals during the decision-making process.

## Methods

### Design

A systematic review of the literature was performed according to the steps of the Cochrane Handbook for Systematic Reviews of Interventions [21]. This review is reported in concordance with the Preferred Reporting Items for Systematic reviews and Meta-Analyses (Additional file 1: PRISMA) statement [22].

### Search strategy

Firstly, the Cochrane database for systematic reviews and the DARE database were checked for a similar review (protocol). No review was identified, therefore systematic searches were performed in MEDLINE (EBSCO), PubMed, CINAHL (EBSCO), and EMBASE (OVID) in June 2016. Search strategies were developed to represent ‘terms for non-conveyance’ AND ‘terms for pre-hospital ambulance care’. Full search strategies per database are given in Additional file 2: Appendix 1. Searches were not restricted by year of publication. In addition to the electronic searches, after full-text inclusion we hand-searched reference lists to identify relevant studies.

### Study selection procedure

We included all types of peer-reviewed systematic reviews, and quantitative or qualitative designs in real clinical practice or simulation situations, on non-conveyance. We defined non-conveyance as ‘the situation where an ambulance was dispatched and where the patient received on-scene diagnostics and/or treatment, followed by professional and/or patient initiated non-conveyance to the ED or another emergency care facility’. Studies were included when reporting on one or more of the following criteria:

- Non-conveyance rates;
- Characteristics of non-conveyed patients;
- Follow-up care after non-conveyance;
- Non-conveyance guidelines, protocols, or on-scene triage criteria;
- Professional competencies needed to initiate non-conveyance;
- Factors influencing the non-conveyance decision-making process.

Conference abstracts, narrative reviews, editorials, personal communications, or unpublished studies were excluded. All articles were screened on title and abstract by two independent reviewers (RE, SB, RS, NT, LV). In case of doubt, a third reviewer (SB, LV) was asked to make a final decision. The remaining articles were screened full text by two independent reviewers (RE, SB, RS, NT, JL, LV). In addition, reference lists of included articles were screened (RE, JL) and potentially relevant publications were screened in a similar way (RE, RS, NT, JL).

### Quality assessment

To assess the risk of bias of (pre-, or quasi-) experimental studies we used the ‘risk of bias assessment tool’ [21]. This tool is a domain-based evaluation to assess selection bias, performance bias, attrition bias, detection bias and reporting bias. For non-randomized studies, the Cochrane collaboration recommends to add additional domains. Therefore, we added two domains to the tool: (1) randomization (yes/no), and (2) control group (yes/no). To assess the quality of systematic reviews we used AMSTAR, as recommended by Cochrane [23]. To assess the quality of observational studies (retrospective, cross-sectional, prospective) and qualitative studies we used tools developed for evaluating primary research papers in a variety of fields [24]. From the 14-criteria quantitative tool, we deleted three criteria (criteria five, six, and seven) on experimental research as we assessed quality of experimental studies with the tool described above. For qualitative studies we used the 10-criteria tool. The quality assessment was performed by couples of two independent researchers (RE, RS, NT, JL). In case of doubt, a third reviewer from these four researchers was asked to make a final decision.

### Data extraction

Data were extracted by two independent researchers (RE, RS, NT, JL). Outcomes extracted were non-conveyance rates, characteristics of non-conveyed patients, existing guidelines, protocols or triage criteria for non-conveyance, follow-up care by patients after non-conveyance, ambulance professionals competencies needed to perform non-conveyance, and factors influencing ambulance professionals during the non-conveyance decision-making process.

### Data synthesis and presentation

Due to heterogeneity of the studies with regard to patient populations, interventions and outcomes, a meta-analysis was not possible. Instead, we extensively analysed and synthesized the studies, by scrutinizing and categorizing data and formulating (sub)themes. To report non-conveyance rates, percentages were extracted or calculated. When patients died or left the scene before ambulance arrival, these were not taken into account for non-conveyance rates. To compare patients’ initial complaints or conditions across studies, we classified these according to the ICD-10 classification [25]. The ICD-10 classification is an international standard to classify diseases or other health problems, and is widely accepted and used. For each ICD-10 category we described the proportions of the patients who had a certain classification.

## Results

### Review statistics

**Fig. 1:**
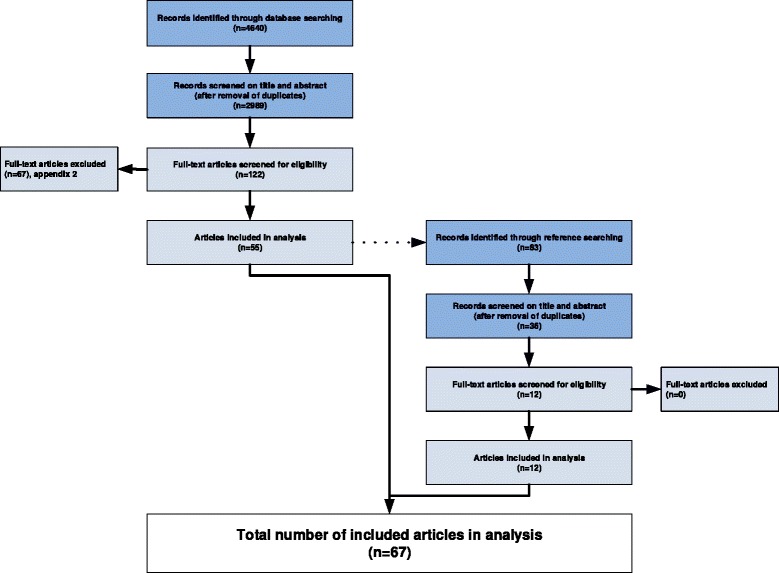
study selection process

The initial search identified 2989 unique records, after the selection procedure 67 studies were included (see Fig. 1). A list of excluded articles (*n* = 67) is provided in Additional file 3: Appendix 2.

### Study Characteristics

The designs of the included studies concerned two systematic reviews [10, 26], four experimental designs: one cluster-randomized controlled trial [27], one quasi-experimental [28], and two pre-test post-test [29, 30], 52 observational designs: 27 retrospective [8, 9, 31–55], 23 prospective [56–78], and two cross-sectional [79, 80], one mixed method design [81], and eight qualitative designs [82–89] (Table 1 and Table 2).

**Table 1:** Characteristics of quantitative and qualitative included studies (*n* = 65)

1st author (Year) Country [ref]	Design	Methods/Data sources	Patients (n)	Professionals (n)
Alicandro (1995) USA [29]	Pre-test post-test	Data card, Online database	Patients (*n* = 361) who refused conveyance	Not described
Alrazeeni (2016) Saudi Arabia [31]	Retrospective, observational	Patient care reports	Patients (*n* = 1791) who were not conveyed	EMTs
Anderson (2002) Denmark [32]	Retrospective, observational	Prehospital database, National Patient Register, Central Personal registry, Registry of Causes of Death	Patients (*n* = 1187) with hypoglycaemia	MICU physicians
Burstein (1996) USA [56]	Prospective, cohort	Identifying card, Telephone follow-up	Patients (*n* = 361) who refused medical assistance	Emergency physicians (*n* = 22), ALS and BLS providers
Burstein (1998) USA [57]	Prospective, observational	10-point assertiveness scale, ED disposition instrument	Patients (*n* = 130) who refused medical assistance	Paramedic medical-control console operators, EMS-crews, Emergency physicians
Burrell (2013) UK [82]	Qualitative	Topic guided in-depth interviews	No patient population included	EMT level 2 (*n* = 1), EMT level (*n* = 4), Paramedics (*n* = 5), Paramedic team leaders (*n* = 4), Emergency care practitioner (*n* = 1)
Cain (2003) USA [58]	Prospective, observational	Patient care report, Refusal form	Ambulance calls (*n* = 17,416)	Basic & advanced paramedics
Carter (2002) Canada [59]	Prospective, observational	Telephone calls, Ambulance call reports	Patients (*n* = 100) with hypoglycaemia receiving IV dextrose	Paramedics, Emergency medicine senior residents
Chen (1996) Taiwan [60]	Prospective, observational	Dispatch record, Ambulance run record, ED disposition form	Patients (*n* = 1035) who called an ambulance	EMTs
Cone (1995) USA [8]	Retrospective, observational	Emergency department records, Telephone follow-up, Ambulance call reports, Medical command control forms	Patients (*n* = 85) who refused conveyance	Paramedics, Volunteer municipal basic life support units
Deasy (2008) Ireland [61]	Prospective observational	Data sheets	Ambulance calls (*n* = 263)	Emergency Medicine Specialists, Paramedics
Ebrahimian (2014) Iran [83]	Qualitative	Semi-structured interviews	No patient population included	EMS staffs (*n* = 18)
Gerlacher (2001) USA [79]	Cross-sectional	Patient records	Patients (*n* = 15,409) ≤ 12 years	First responder firefighters, EMTs, Paramedics
Goldstein (2015) Canada [33]	Retrospective, observational	Electronic patient care records	Patients (*n* = 63,067) ≥ 65 years	Primary care paramedics, Intermediate care paramedics, Advanced care paramedics
Haines (2006) USA [62]	Prospective, observational	Telephone follow-up questionnaire, Ambulance records	Patients (*n* = 5336) <21 years	ALS-paramedics, Physicians
Halter (2011) UK [84]	Qualitative	Semi-structured interviews	No patient population included	EMTs, Paramedics (*n* = 12)
Hipskind (1997) USA [63]	Prospective, observational	Ambulance run reports	Patients (*n* = 683) who refused conveyance	Paramedics (n ≈ 350)
Højfeld (2014) Denmark [34]	Retrospective, observational	MECU database, Medical records	Mobile emergency care unit runs (*n* = 15,392)	Anaesthesiologists
Jensen (2013) Canada [64]	Prospective, observational	Data from emergency health services, Patient care records, Databases	Ambulance calls (*n* = 265) for long term care facility patient	Extended care paramedics (*n* = 7), Paramedics
Kahalé (2006) Canada [65]	Prospective, observational	Ambulance call reports, Hospital charts, Telephone interviews	Patients (*n* = 345) <16 years	EMTs, Paramedics
Kamper (2001) USA [35]	Retrospective, observational	Ambulance run records, ED records, Hospital records	Ambulance calls (*n* = 53,627)	Paramedics
Kannikeswaran (2007) USA [36]	Retrospective, observational	Standardized data extraction sheets	Ambulance runs (*n* = 5976) for children <18 years	EMT-Ps, EMT-Bs
Keene (2015) Australia [85]	Mixed-methods	Structured interviews, Patient care records	Patients (*n* = 33,333) where an ambulance was dispatched	Ambulance paramedics, Intensive care paramedics
Key (2003) USA [30]	Pre-test post-test	Patient/ambulance records	Ambulance calls (*n* = 11,488)	Paramedics, EMTs
Knight (2003) USA [37]	Retrospective, descriptive	State-wide EMS data, State-wide ED data, Death certificate data	EMS dispatches (*n* = 277,221)	Not described
Lerner (2003) USA [66]	Prospective, observational	Telephone interviews	Patients (*n* = 36) with hypoglycaemia	EMT-Ps (*n* = 23)
Magnusson (2016) Sweden [38]	Retrospective, observational	Patient notes	Patients (*n* = 529) with low priority, uncertain need for ambulance and vague symptoms	Ambulance nurses
Marks (2002) UK [9]	Retrospective, observational	Patient report forms	Patients (*n* = 500) not conveyed	EMTs, Paramedics
Mechem (1998) USA [67]	Prospective, observational	Telephone interviews	Ambulance calls (*n* = 115,135)	Nurses, Paramedics
Minhas (2015) Canada [39]	Retrospective, cohort	EMS patient records, ED patient records	Patients (*n* = 286) 18–65 years with supraventricular tachycardia	ALS paramedics
Moss (1998) USA [40]	Retrospective, observational	Prehospital records	EMS responses (*n* = 6512)	Paramedics
Murphy-Jones (2016) UK [86]	qualitative, phenomenological	Semi-structured interviews	No patient population included	Paramedics (*n* = 6)
Newton (2015) South-Africa [68]	Prospective, observational	Computerized dispatch logs, Patient report forms	Ambulance calls (*n* = 1689)	BLS emergency care providers, ILS emergency care providers, ALS emergency care providers
O’Hara (2015) UK [87]	Qualitative	Reviewing relevant national and local documents (Reports, policies, protocols), Semi-structured interviews, Observations, Digital diaries, Informal interviews, Focus groups, Written notes	No patient population included	Directors, Managers, Specialist paramedics, Paramedics, Emergency care assistants/technicians/support workers
Persse (2002) USA [69]	Prospective, observational	Patient care records, Structured telephone interviews	Patients (*n* = 2207) ≥ 65 years	Paramedics, EMTs
Peyravi (2013) Iran [41]	Retrospective, observational	National data registry, Ambulance station data registry	Ambulance runs (*n* = 84,084)	Nurses, Paramedics, GPs
Peyravi (2015) Iran [42]	Retrospective observational	Patient care records, Telephone interviews	Ambulance runs (*n* = 81,999)	Not described
Porter (2007) UK [88]	Qualitative	Focus groups (*n* = 3)	No patient population included	Paramedics (*n* = 25)
Pringle (2005) USA [43]	Retrospective, observational	EMS reports, Telephone interviews	EMS patient encounters (*n* = 1894)	EMT-Bs, Paramedics
Rudolph (2011) Denmark [44]	retrospective, observational	Medical emergency care unit database, Autopsy reports	Patients (*n* = 4762) with acute opioid overdose	Anaesthesiology specialists, ALS providers
Schmidt (2001) USA [70]	Prospective, observational	Patient records	Patients (*n* = 1433) were an ambulance was dispatched	EMT-Ps, EMT-ILSs, EMT-Bs
Schmidt (1998) USA [71]	Prospective, observational	Structured telephone interview	Patients (*n* = 324) who refused conveyance	Paramedics
Schmidt (2000) USA [72]	Prospective observational	Data sheets	Patients (*n* = 1433) where an ambulance was dispatched	EMT-Ps, EMT-ILSs, EMT-Bs
Schmidt (2006) USA [45]	retrospective, observational	EMS database	Ambulance runs (*n* = 1501)	Paramedics
Selden (1990) USA [46]	Retrospective, observational	Run records	Ambulance runs (*n* = 11,780)	Paramedics
Seltzer (2001) USA [47]	Retrospective, observational	Run records, Structured telephone interviews	Patients (*n* = 89) <18 years who refused conveyance against medical advice	EMT-Ds, EMT-Ps
Shaw (2006) UK [81]	Mixed methods	Patient records	Ambulance runs (*n* = 76,635)	Paramedics, EMTs
Simpson (2014a) Australia [74]	Prospective, cohort	Data sheets, Administrative databases	Patients (*n* = 1610) ≥65 years who have fallen	Paramedics
Simpson (2014b) Australia [73]	Prospective, cohort	Data collection tool, Dispatch system	Patients (*n* = 1610) ≥65 years who have fallen	Paramedics (*n* = 384)
Snooks (2005) UK [89]	Qualitative	Focus groups	No patient population included	Paramedics (*n* = 26)
Snooks (2014) UK [27]	CRCT	Paramedic records, ED records	Patients (*n* = 779) ≥65 years who have fallen	Paramedics (*n* = 42)
Snooks (2004a) UK [28]	Quasi-experimental	Patient report forms, ED records, GP records, Questionnaire	Patients (*n* = 797) were an ambulance was dispatched	Paramedics (*n* = 5), EMTs (*n* = 5)
Socransky (1998) USA [48]	Retrospective, observational	Patient records, Hospital records	Ambulance runs (*n* = 10,888)	Paramedics
Stark (1990) USA [49]	Retrospective, observational	EMS database	Ambulance calls (*n* = 1715)	Paramedics, Physicians
Staudenmayer (2012) USA [50]	Retrospective, cohort	Population-based injury database	Patients (*n* = 69,413) with a primary diagnosis of ‘injury’ or ‘trauma’	Not described
Strote (2008) USA [75]	Prospective, cohort	Medical incident report forms, Telephone interviews	Patients (*n* = 2359) with hypoglycaemia	EMTs, Paramedics
Stuhlmiller (2005) USA [51]	Retrospective, observational	On-line medical command audio tapes, Patient run sheets, Non-conveyance sheets	On-line medical control calls (*n* = 137) for patient-initiated refusals	Paramedics
Tiedemann (2013) Australia [76]	Prospective, cohort	Patient records, Questionnaires (e-mail)	Patients (*n* = 2842) ≥70 years who have fallen	Paramedics
Tohira (2016a) Australia [53]	Retrospective cohort	Patient care records, ED information system, Death registry	Patients (*n* = 1238) post-ictal or with hypoglycaemia	Paramedics
Tohira (2016b) Australia [52]	Retrospective, cohort	Patient care records, ED information system, Death registry	Patients (*n* = 127,574) were an ambulance was dispatched	Paramedics
Van der Pols (2011) Netherlands [77]	Prospective, cohort	Patient record, Hospital databases, Dispatch centre database	Patients (*n* = 1842) were an ambulance was dispatched	Ambulance nurses
Vilke (1999) USA [54]	Retrospective, observational	Prehospital database, Death registry	Patients (*n* = 94,466) were an ambulance was dispatched	Paramedics
Vilke (2002) USA [78]	Prospective, observational	Telephone interviews	Patients (*n* = 636) ≥ 65 years and who signed out against medical advise	EMTs, EMT-Ps, EMT-Ds
Zachariah (1992) USA [55]	Retrospective, observational	Patient records, Structured telephone interviews	Patients (*n* = 158) not conveyed	Paramedics
Zorab (2015) UK [80]	Cross-sectional	Questionnaires	No patient population included	Emergency Care Assistants, Ambulance Technicians, Student Paramedics, Paramedics, Emergency Care Practitioners, Critical Care Paramedics

Abbreviations: *ALS* Advanced life Support, *BLS* Basic Life Support, *ED* Emergency Department, *EMD* emergency medical department, *EMS* Emergency Medical Service, *EMT* Emergency Medical Technician, *EMT-B* Emergency Medical Technician Basic, *EMT-D* Emergency Medical Technician Defibrillation, *EMT-ILS* Emergency Medical Technician Intermediate Life Support, *EMT-P* Emergency Medical Technician Paramedics, *GP* general practitioner, *ILS* Intermediate Life Support, *MECU* Mobile Emergency Care Unit, *MICU* Mobile Intensive Care Unit

**Table 2:** Characteristics systematic reviews (*n* = 2)

1st author (year) country	Aim	Databases	Selection criteria	Included articles
Mikolaizak (2013) Australia [26]	To summarize the evidence in relation to (1) non-conveyance rates, (2) outcomes following non-conveyance, and (3) outcomes from alternative care pathways for non-conveyed older people who have fallen	1. Medline 2. Embase 3. CINAHL 4.PsycINFO 5.Cochrane Library 6. Web of Science	1. Peer-reviewed articles 2. Original data relating to non-transport rates for older people who have fallen 3. Outcomes on falls or outcomes for alternate care pathways for non-transported people who have fallen	12 articles: 2 randomized controlled trials, 5 prospective cohort studies, 4 retrospective cohort studies and 1 historical cohort trial.
Snooks (2004b) UK [10]	1. To describe outcomes of non-conveyed patients 2. To describe triage ability of crews 3. To assess effectiveness and safety of protocols that allow crews to convey patients to alternative receiving units or to self-care	1. Medline 2. BIDS 3.Healthplan 4. Helmis	Articles on paramedics trained with extra skills to perform tasks beyond their baseline competencies	31 articles: 13 retrospective observational studies, 8 prospective observational studies, 6 cross-sectional studies, 3 case studies and 1 quasi-experimental study

The two systematic review were performed in Australia and the UK. The empiric studies were conducted in North America (*n* = 36), Europe (*n* = 17), Australia (*n* = 6), Asia (*n* = 5), and Africa (*n* = 1), and concerned general patient populations or specific patient populations, including patients with hypoglycaemia, patients who refused conveyance, paediatric and/or older patients, patients with supraventricular tachycardia, patient with acute opioid overdose, post-ictal patients, and patients who had fallen. The ambulance professionals in these studies were ambulance nurses, basic and advanced life support paramedics, emergency medical technicians, (specialized) physicians, general practitioners, and first responder fire fighters. For this review we will use the term ‘ambulance professional’ to cover all these types of professionals.

### Quality assessment (Additional file 4: Appendix 3, Additional file-5: Appendix 4, Additional file 6: Appendix 5, Additional file 7: Appendix 6)

The two included systematic reviews had moderate [26] and low quality [10] (Additional file 4: Appendix 3). The four experimental designs included one CRCT of moderate quality [27], one quasi-experimental study [28] and two pre-test post-test [29, 30] of poor quality (Additional file 5: Appendix 4). The quality of the quantitative studies (*n* = 53) varied from good [76] to poor [42] (Additional file 6: Appendix 5), and the quality of the qualitative studies (*n* = 8) varied from good [83] to poor [88] (Additional file 7: Appendix 6).

### Outcomes

#### Non-conveyance rates (Additional file 8: Appendix 7)

Non-conveyance was initiated by the ambulance professional, the patient and/or his relatives, or a joint decision. Non-conveyance rates for general patient populations ranged from 3.7% up to 93.7% [28, 30, 31, 33–35, 37, 38, 40–43, 45, 46, 49, 51, 52, 57, 60, 61, 64, 68, 77, 81]. Seventeen studies reported non-conveyance rates for specific patient populations. For patients with *hypoglycaemia* non-conveyance rates ranged from 12.2% up to 84.3% [32, 48, 53, 58, 59, 75]. Non-conveyance rates for *people who had fallen* ranged from 11%–56% [26, 27, 73, 74]. For *paediatric* patients non-conveyance rates ranged from 13.2%–27.7% [36, 62, 79]. Two studies reported non-conveyance rates for patients with an *opioid overdose*, ranging from 6.0%–77.0% [44, 54]. Non-conveyance rates for other specific patient groups were 14.0% for *post-ictal patients* [53], 33.2% for patients with *supraventricular tachycardia* [39], 10.7%–11.5% for *elder people* [69], and 8.6% for patients with *injuries* [50].

#### Characteristics of non-conveyed patients (Additional file 8 Appendix 7)

The demographic characteristics were age, gender, ethnicity, and geographic area. For general patient populations, the *age* ranges from 14 up to 90 years [9, 29, 31, 33, 38–40, 45, 48, 50, 52, 54, 56, 62, 63, 65–67, 73, 74, 76, 78, 79, 85]. Twenty studies reported on patient gender: in ten studies the *gender* is predominantly male, in the other studies the population is predominantly female [9, 33, 38–40, 45, 48, 50, 52, 54, 62, 63, 65–67, 73, 74, 76, 79, 85]. Three studies described the *geographic location* of non-conveyed patients [33, 65, 74]. Two of these show that most non-conveyed people stay in a metropolitan/urban area. The third study showed that 58.6% of the patient are in their residence. Two studies described the patient’s *ethnicity* [45, 79], with one study reporting 90.6% of the non-conveyed patient as white, the other study reported 48.3% of the patient as African-American.

The clinical characteristics of the patient were initial complaints and conditions, vital signs, and patient history. A variety of *initial complaints and conditions* was described [9, 29, 34, 38, 40, 45, 52, 56, 57, 61–63, 65, 74, 77–79, 85]. Most often, we found initial complaints and conditions classified as *VI- diseases of the nervous* system (*n* = 16) or *category XX - External causes of morbidity and mortality* (*n* = 16). For category *VI* the proportion of patients with these complaints and conditions ranged from 1.0%–29.0% [9, 29, 34, 38, 40, 45, 52, 56, 57, 61, 63, 65, 77–79, 85], for category *XX* the proportion ranged from 11.0%–68.5% [9, 29, 38, 40, 45, 52, 56, 57, 61–63, 65, 77–79, 85].

Three studies described the *vital signs* of non-conveyed patients [50, 52, 63]. One study on a general population reported that 14.9% of the non-conveyed patients had abnormal vital signs (blood pressure, O_2_-saturation, Glasgow Coma Scale, and body temperature) [52]. A second study in a non-conveyed general patient population reported that 70.0% had a blood pressure within normal limits, 72.2% had a heart rate within normal limits, and 63.2% had a respiratory rate within normal limits [63]. The last study on vital signs with injured people not conveyed reported a mean systolic blood pressure of 134.7 mmHg (±21.1), a mean pulse rate of 91.8 (±15.9), and a mean Glasgow Coma Scale of 15.0 (±0.3) [50].

Five studies described the patient’s history by describing the *medical history and/or current medication use* [48, 63, 73, 74, 76]. Two studies [63, 76] described the medical history, for general patient populations 68.7% had no medical history [63], for people aged ≥70 years who had fallen 43.8% had urinary incontinence and 39.0% had a central nervous system disorder.

#### Follow-up of patients after non-conveyance (Table 3)

Follow-up was reported as (a) repeated access to healthcare and (b) patient outcomes. Sixteen studies combined these outcome categories, the other studies used outcomes within one category [8, 26, 28, 32, 37–40, 43–45, 48, 50, 52, 55–59, 62, 64–67, 69, 75–78, 90]. Repeated access to healthcare was specified as repeated access to (1) emergency department (2) EMS-system (call or ambulance run), (3) the general practitioner, and (4) walk-in clinic. For all outcomes, a variety of follow-up periods was used. In every study that reported on repeated access to healthcare a proportion of patients re-entered the (emergency) healthcare system.

**Table 3:** Follow-up care after non-conveyance

1st author (year) Country [ref]	Follow-up outcomes	Results
Anderson (2002) Denmark [32]	• Patient outcome – hospitalization • Patient outcome – recurrent symptoms	• 76/968 (7.9%) patients have secondary blood glucose regulatory problems <72 h ◦ 46/76 (60.5%) have a recurrent hypoglycaemia, 33/46 (71.7%) of these cases occur <24-72 h • 49/968 (5.1%) are hospitalized <72 h ◦ 21/49 (42.9%) have a recurrent hypoglycaemia of which 12/21 (57.1%) are hospitalized <24-72 h
Burstein (1996) USA [56]	• Repeat access general healthcare – GP • Repeat access emergency healthcare – EMS (call or EMS run) • Repeat access emergency healthcare – ED	• 199/321 (62.0%) patients who had follow-up. ◦ 95/199 (47.7%) patients sought additional medical care <1 week. ▪ 51/95 (53.7%) went to the ED: 7 through EMS, 41 referred themselves to the ED and 3 were referred by their physician. ▪ 44/95 (46.3%) were seen by their physician.
Burstein (1998) USA [57]	• Repeat access general healthcare – GP • Repeat access emergency healthcare – ED • Patient outcome – mortality • Patient outcome – hospitalization	• 66/69 (95.7%) patients could be contacted through follow-up <2–3 days ◦ 33/66 (50.0%) patients saw their own physicians ◦ 17/66 (25.8%) went to an ED on their own ◦ 8/66 (12.1%) were admitted to the hospital ◦ 4/66 (6.1%) died
Cain (2003 USA [58]	• Repeat access emergency healthcare – EMS (call or EMS run)	40/145 (27.6%) patients had signs and symptoms compatible with low blood sugar occurring <10 months after initial event and requiring a repeat EMS call: • 2/24 (8.3%) patients >65 years • 38/121 (31.4%) patients <65 years 3/145 (2.1%) patients had signs and symptoms compatible with low blood sugar occurring <48 h after initial event and requiring a repeat EMS call: • 0/24 (0.0%) patients >65 years • 3/121 (2.5%) patients <65 years • No significant differences in repeat (*p* = .43) any time during the ten-month study period, recurrences (*p* = .33) <48 h and interval for repeat episodes (*p* = .60) between conveyed and non-conveyed patient calls.
Carter (2002) Canada [59]	• Patient outcome – recurrent symptoms	Repeated access to healthcare <21 days: • 6/41 (14.6%) patients for all complaints • 2/41 (4.9%) patients for the same complaint
Cone (1995) USA [8]	• Repeat access general healthcare – GP • Repeat access emergency healthcare – ED • Patient outcome – hospitalization	54/81 (67%) had follow-up: • 37/54 (68.5%) sought no medical care • 10/54 (18.5%) were evaluated in the ED: 3 were discharged, 7 were admitted: 3 were admitted to monitored beds and 4 were admitted to unmonitored beds • 7/54 (13.0%) saw their own physician <48 h after refusal
Haines (2006) USA [62]	• Repeat access general healthcare – GP • Repeat access emergency healthcare – ED • Patient outcome – hospitalization	527/704 (74.8%) completed phone follow-up: • 13/527 (2.5%) non-transport group hospitalized • 279/527 (52.9%) patients had follow-up-care <72 h (median 2.5 h, inter-quartile range 1.5–13 h) ◦ 203/279 (72.6%) patients had follow-up-care <12 h ◦ 148/279 (65.9%) patients came to ED ◦ 95/279 (34.1%) patients came via primary care physician ◦ 19/279 (6.8%) patients were evaluated by a medical provider more than once in 72 h
Højfeld (2014) Denmark [34]	• Repeat access emergency healthcare – ED • Patient outcome – mortality • Patient outcome – hospitalization	113/1609 (7.0%) patients had renewed treatment in hospital or ED <24 h ◦ 58/113 (51.3%) had to be admitted ◦ 51/113 (45.1%) visited the ED ◦ 4/113 (3.5%) died
Jensen (2013) Canada [64]	• Repeat access emergency healthcare – EMS (call or EMS run)	6/238 (2.5%) patients who received extended paramedic care but who were not transported subsequently triggered a EMS call <48 h
Kahalé (2006) Canada [65]	• Repeat access general healthcare – GP • Repeat access general healthcare – walk-in clinic • Repeat access emergency healthcare – ED	51/345 (14.8%) non-transported children were seen at the ED <48 h Telephone follow-up with patients (*n* = 106) about additional care <48 h: • 51/106 (48.1%) patients did not seek medical follow-up • 28/106 (26.4%) patients went to the ED • 22/106 (20.8%) patients visited the family physician/paediatrician office • 4/106 (3.8%) patients visited a walk-in clinic • 1/106 (0.9%) patients went to a hospital/outpatient clinic
Knight (2003) USA [37]	• Repeat access emergency healthcare – ED • Repeat access emergency healthcare – EMS (call or EMS run) • Patient outcome – mortality • Patient outcome – hospitalization	3454/26574 (13.0%) follow-up was obtained <1 week: • 174/3454 (5.0%) patients were admitted to the hospital • 25/3454 (0.7%) patients died • 465/3454 (13.5%) patient had an EMS dispatch ◦ < 3 years: 8/465 (1.7%) ◦ 3–12 years: 14/465 (3.0%) ◦ 13–17 years: 24/465 (5.2%) ◦ 18–64 years: 301/465 (64.7%) ◦ ≥ 65 years: 118/465 (25.4%) • 2790/3454 (80.1%) of the patients had an ED visit ◦ < 3 years: 133/3454 (3.9%) ◦ 3–12 years: 175/3454 (5.1%) ◦ 13–17 years: 223/3454 (6.5%) ◦ 18–64 years: 2041/3454 (59.1%) ◦ ≥ 65 years: 218/3454 (6.3%) • 174/3454 (5.0%) of the patients were admitted ◦ < 3 years: 12/174 (6.9%) ◦ 3–12 years: 13/174 (7.5%) ◦ 13–17 years: 7/174 (4.0%) ◦ 18–64 years: 97/174 (55.7%) ◦ ≥ 65 years: 45/174 (25.9%)
Lerner (2003) USA [66]	• Repeat access general healthcare – GP • Repeat access emergency healthcare – ED	20/36 (55.6%) sought further medical assistance <48 h: • 11/20 (55.0%) called their personal physician • 8/20 (40.0%) visited their personal physician • 1/20 (5.0%) went to the ED
Magnusson (2016) Sweden [38]	• Repeat access general healthcare – GP • Repeat access emergency healthcare – ED • Patient outcome – hospitalization	38/200 (19.0%) patients visited the ED <72 h: • 24/38 (63.2%) self to ED ◦ 12/24 (50.0%) admitted • 14/38 (36.8%) referred by GP ◦ 8/14 (57.1%) admitted
Mechem (1998) USA [67]	• Repeat access general healthcare – GP • Repeat access emergency healthcare – ED • Repeat access emergency healthcare – EMS (call or EMS run) • Patient outcome – hospitalization	94/103 (91.3%) patients had no recurrence of symptoms in <72 h: • 7/94 (7.4%) contacted private physician 9/103 (8.7%) recontacted the *EMS <* 72 h*:* • 5/9 (55.6%) transported and released from ED • 3/9 (33.3%) transported and admitted • 1/9 (11.1%) refused transport
Mikolaizak (2013) Australia [26]	• Repeat access general healthcare – GP • Repeat access general healthcare – walk-in clinic • Repeat access emergency healthcare – ED • Repeat access emergency healthcare – EMS (call or EMS run) • Patient outcome – mortality • Patient outcome – hospitalization	Follow-up periods varied from 1 to 12 months. Outcomes: 12%–49% readmission in ambulance or other health service facility, non-transported patients have significantly higher risk of death compared to age matched peers
Minhas (2015) Canada [39]	• Repeat access emergency healthcare – EMS (call or EMS run)	1/76 (1.3%) of the patients treated and released had 14 representations <72 h
Moss (1998) USA [40]	• Repeat access emergency healthcare – ED • Repeat access emergency healthcare – EMS (call or EMS run) • Patient outcome – mortality • Patient outcome – hospitalization	431/443 (97.3%) patients a follow-up was obtained: • 10/431 (2.3%) called EMS again <48 h ◦ 4/10 (40.0%) were admitted to a hospital ◦ 4/10 (40.0%) were discharged from the ED ◦ 1/10 (10.0%) died ◦ 1/10 (10.0%) was transferred to another facility
Persse (2002) USA [69]	• Patient outcome – hospitalization	Phase 1: 151/254 (59.5%) patients were contacted by telephone: • 56/151 (37.1%) sought further medical help <24 h • 19/151 (12.6%) were hospitalized Phase 2: 109/198 (55.1%) patients were contacted by telephone: • 37/109 (33.9%) sought further medical help <24 h • 7/109 (6.4%) were hospitalized
Pringle (2005) USA [43]	• Patient outcome – mortality • Patient outcome – hospitalization	310/906 (34.2%) follow-up was obtained (1 week): • 172/310 (55.5%) patients sought medical care: ◦ 106/172 (61.6%) medical care was changed • 25/310 (8.1%) were admitted to a hospital • 1/310 (0.3%) patients died
Rudolph (2011) Denmark [44]	• Patient outcome – mortality	18/2241 (0.8%) patients released on scene died <48 h
Schmidt (2006) USA [45]	• Patient outcome – mortality	2/128 (1.6%) patients not-transported died <30 days
Snooks (2004a) UK [28]	• Patient outcome – hospitalization	Intervention group: 5/93 (5.4%) patients were admitted to a hospital <14 days Control group: 12/195 (6.2%) patients were admitted to a hospital <14 days
Socransky (1998) USA [48]	• Repeat access emergency healthcare – ED • Patient outcome – hospitalization • Patient outcome – recurrent symptoms	25/412 (6.1%) of the patients who refused transport had a relapse <48 h: • 14/25 (56.0%) refused transport again • 6/25 (24.0%) admitted to the ED • 5/25 (20.0%) were admitted to a hospital
Staudenmayer (2011) USA [50]	• Repeat access emergency healthcare – ED • Patient outcome – hospitalization • Patient outcome – mortality	1715/5865 (29.2%) follow-up obtained: • 1616/1715 (94.2%) patients were seen in the ED and discharged • 92/1715 (5.4%) were admitted to the hospital • 7/1715 (0.4%) died
Strote (2008) USA [75]	• Repeat access general healthcare – GP • Repeat access emergency healthcare – ED • Patient outcome – hospitalization	203/402 (49.5%) follow-up obtained: • 111/203 (54.7%) patients contacted their primary care physician <24 h • 8/203 (3.9%) patients called the EMS again <48 h • 16/203 (7.9%) patients went to the hospital <48 h
Tiedemann (2013) UK [76]	• Patient outcome – recurrent symptoms	62/251 (24.7%) of the non-transported patients required ≥1 fall related repeat ambulance attendance <6 months
Tohira (2016b) Australia [52]	• Repeat access emergency healthcare – ED • Repeat access emergency healthcare – EMS (call or EMS run) • Patient outcome – mortality • Patient outcome – hospitalization	Subsequent events after discharge at the scene, Unadj OR (95% CI) ∗ Adj OR (95% CI)∗ Ambulance request • Within 1 day 672/11096 (6*.*1%) 3*.*5 (3*.*1–4*.*0) 3*.*4 (3*.*0–3*.*9) • Within 3 days 995/11096 (9*.*0%) 2*.*3 (2*.*1–2*.*5) 2*.*1 (1*.*9–2*.*4) • Within 7 days 1305/11096 (11*.*8%) 1*.*9 (1*.*7–2*.*0) 1*.*7 (1*.*6–1*.*9) ED attendance • Within 1 day 514/11096 (4*.*6%) 3*.*4 (3*.*0–3*.*9) 3*.*3 (2*.*8–3*.*8) • Within 3 days 710/11096 (6*.*4%) 2*.*0 (1*.*8–2*.*2) 1*.*9 (1*.*7–2*.*2) • Within 7 days 898/11096 (8*.*1%) 1*.*5 (1*.*4–1*.*6) 1*.*4 (1*.*2–1*.*5) Hospitalisation • Within 1 day 361/11096 (3*.*3%) 4*.*1 (3*.*5–4*.*9) 4*.*2 (3*.*4–5*.*1) • Within 3 days 500/11096 (4*.*5%) 2*.*5 (2*.*2–2*.*9) 2*.*3 (2*.*0–2*.*7) • Within 7 days 634/11096 (5*.*7%) 2*.*0 (1*.*8–2*.*2) 1*.*8 (1*.*6–2*.*0) Death • Within 1 day 19/11096 (0*.*2%) 1*.*6 (0*.*9–2*.*8) 1*.*8 (0*.*99–3*.*2) • Within 3 days 32/11096 (0*.*3%) 1*.*7 (1*.*1–2*.*6) 1*.*9 (1*.*2–3*.*0) • Within 7 days 56/11096 (0*.*5%) 1*.*6 (1*.*2–2*.*3) 1*.*8 (1*.*3–2*.*5) ∗ vs. ED-discharge
Van der Pols (2011) The Netherlands [77]	• Repeat access general healthcare – GP	Motorcycle response vehicles with one ambulance nurse with additional training (*n* = 468) compared to regular ambulance (*n* = 1196): • referral to GP 138/468 (29.5%) vs 167/1196 (14.0%) RR 2.11 (95%CI 1.73–2.58)
Vilke (2002) USA [78]	• Repeat access general healthcare – GP • Repeat access general healthcare – walk-in clinic • Repeat access emergency healthcare – ED • Repeat access emergency healthcare – EMS (call or EMS run)	71/121 (58.7%) follow-up was obtained: • 27/71 (38.0%) visited family physician • 25/71 (35.2) visited urgent care facility • 9/71 (12.7%) second EMS call and transported to ED • 9/71 (12.7%) transport to ED by private vehicle • 1/71 (1.4%) second EMS call and treated at scene
Zachariah (1992) USA [55]	• Repeat access general healthcare – GP • Patient outcome – hospitalization	93/158 (58.9%) follow-up was obtained: • 60/93 (64.5%) sought care from a physician: ◦ 15/60 (25.0%) were admitted to hospital.

*Repeated access to the ED* is measured in seventeen studies [8, 26, 37, 38, 40, 48, 50, 52, 56, 57, 62, 65–67, 75, 78, 90]. For general patient populations, the follow-up periods ranged from <24 h up to <7 days, and repeated access percentages varied from 4.6–7.0% (<24 h), 19.0% (<48 h), 6.4–25.8% (72 h) up to 8.1–80.1% (<7 days). For specific patient populations (hypoglycaemia, people who had fallen, people aged >65 years, children and people with minor injuries), the follow-up periods ranged from <48 h up to <12 months, and repeated access percentages varied from 5.0–26.4% (<48 h), 65.9% (<72 h), up to 12.0–49.0% (12 months).

*Repeated access to the EMS-system* is measured in ten studies [26, 37, 39, 40, 52, 56, 58, 64, 67, 78]. For general patient populations, the follow-up periods ranged from <24 h up to <7 days, and repeated access percentages varied from 6.1% (<24 h), 2.3–2.5% (<48 h) up to 7.4–13.5% (<7 days). For specific patient populations (hypoglycaemia, people who had fallen, supraventricular tachycardia, and people aged >65 years), the follow-up periods ranged from <48 h up to <12 months, and repeated access percentages varied from 0.0–2.5% (<48 h), 1.3–8.7% (<72 h), 8.3–31.4% (10 months) up to 12.0–49.0% (12 months).

*Repeated access to the GP* is measured in thirteen studies [8, 26, 38, 55–57, 62, 65–67, 75, 77, 78]. For general patient populations, the follow-up periods ranged from <48 h up to <7 days, and repeated access percentages varied from 13.0% (<24 h), 36.8–50.0% (<72 h) up to 46.2% (<7 days). For specific patient populations (hypoglycaemia, people who had fallen, children, and people aged >65 years), the follow-up periods ranged from <24 h up to 12 months, and repeated access percentages varied from 54.7% (24 h), 7.4–40.0% (<48 h), 34.1% (72 h) up to 12.0–49.0% (12 months).

*Repeated access to walk-in clinic* is measured in three studies for specific patient populations (children, people who had fallen, and patients aged >65 years) [26, 65, 78]. The follow-up periods used for this outcome ranged from <48 h up to 12 months, and repeated access percentages varied from 3.8% (<48 h) up to 12.0–49.0% (12 months).

The patient outcomes measured are mortality, hospitalization and recurrence of symptoms. For general patient populations, the follow-up periods for *mortality* ranged from <24 h up to <30 days, and mortality rates ranged from 0.2–3.5% (<24 h), 0.3% (<48 h), 0.3–6.1% (<72 h), 0.3%–0.7% (<7 days) up to 1.6% (<30 days) [26, 34, 37, 40, 43, 45, 50, 52, 57]. The one study reporting on a specific patient population (opioid overdose) reported a 0.8% mortality rate < 48 h [44].

The *hospitalization* follow-up period for general patient populations ranged from <24 h up to <14 days, and hospitalization rates ranged from 3.3% (<24 h), 1.0% (<48 h), 4.5–12.1% (<72 h), 5.0–8.1% (<7 days) up to 5.4–6.2% (<14 days) [8, 28, 34, 37, 38, 40, 43, 52, 55, 57]. For specific patient populations (hypoglycaemia, people who had fallen, children, people with minor injuries, and people aged >65 years) the follow-up periods ranged from <48 h up to 12 months, and hospitalization rates ranged from 1.2–7.9% (<48 h), 2.5–5.1% (<72 h) up to 12.0–49.0% (<12 months) [26, 32, 48, 50, 62, 67, 69, 75].

*Recurrence of symptoms* for specific patient populations (hypoglycaemia and people who had fallen) varied from 6.1% (48 h), 7.9% (<72 h), 4.9% (<21 days) up to 24.7% (<6 months) [32, 48, 59, 76].

#### Existing guidelines, protocols and triage criteria for non-conveyance (Additional file 9: Appendix 8)

Criteria to guide the (non-) conveyance decision described mostly are abnormal vital functions related to ‘breathing’ (respiration rate, respiratory distress, dyspnea), abnormal vital functions related to ‘circulation’ (systolic/diastolic blood pressure, pulse), suspected or confirmed ingestion of alcohol or drugs, and an altered level of consciousness (Glasgow coma scale) [28, 29, 39, 40, 43, 46, 51–54, 59, 70, 72, 73, 75, 79, 84, 88]. Ten of these studies described more specific flowcharts, tools, checklist or standard operating procedures for non-conveyance in general [43, 51, 72], patients who refuse conveyance [29, 40, 46], and patients who had fallen [84], with supraventricular tachycardia [39], with social problems [28], with hypoglycaemia [53], and post-ictal patients [53].

#### Professionals competencies and other factors influencing the non-conveyance decision-making process (Table 4)

Factors influencing the non-conveyance decision-making process are related to the professional, the patient and his relatives, the healthcare process/system, or supportive tools [26, 29, 49, 51, 57, 64, 65, 74, 77, 78, 80, 83–89] (Table 4). These factors can be present at (a) pre-arrival, when the professional forms an early opinion based on information from the emergency call, during (b) initial patient contact where the ambulance professional gets a first impression of the patient, during (c) patient assessment of vital signs and other parameters, and (d) during the actual (non-) conveyance decision moment [84].

**Table 4:** Competences and influencing factors (*n* = 18)

Authors (publication year) country [ref]	Competences/influencing factors	Type of factor
Alicandro (1995) USA [29]	The implementation of a (1) high risk card (T1) and (2) online medical control (T2) for patients with high-risk criteria improved the transport rate: T0 2/60 (3.3%)- T1 7/70 (10.0%) - T2 12/34 (35.3%) *p* = .00003	1. Supportive tools 2. Healthcare process/system
Burstein (1998) USA [57]	The implementation of medical control by telephone to convince patients who attempt refusal of medical care to be transported to the ED: 61/130 (47%) of the patients was convinced	1. Healthcare process/system
Ebrahimian (2014) Iran [83]	Affecting factors of EMS staffs’ decision about transporting: 1. patient’s condition: a. Physical health status b. Socioeconomic status: i. Patient support system ii. Patient and his family’s educational status iii. Patient and his family’s financial status c. Cultural background: i. Confidence ii. Believes and attitudes 2. The context of the EMS mission: a. Characteristics of the mission b. EMS staffs’ characteristics	1. Patient/relative 2. Healthcare process/system
Halter (2011) UK [84]	Influencing factors: 1. Pre-arrival: forming an early opinion from information from the emergency call 2. Initial contact: assessing the need for any immediate action and establishing a report 3. Continuing assessment: gathering and assimilating medical and social information 4. Making a conveyance decision: negotiation, referral and professional defense using professional experience, instinct	1. Healthcare process/system
Jensen (2013) Canada [64]	Extended care paramedics received additional specialized training in the following “extended care” roles: 1. Geriatric assessments and management 2. End-of-life care 3. Primary wound closure techniques (suturing, tissue adhesive) 4. Point-of-care testing. LTC patients treated by ECPs remained at the LTC facility in 98 of 140 (70%) cases, compared to 21 of 98(21.4%) of emergency paramedic calls.	1. Professional
Kahalé (2006) Canada [65]	Reasons for non-transport as cited in parent/patient interviews (*n* = 106): 1. 31/106 (29.2%) EMS-personnel stated that transport was unnecessary 2. 25/106 (23.6%) parents thought that going to the hospital was unnecessary 3. 22/106 (20.8%) parents wanted to use another method of transportation to seek medical care 4. 5/106 (4.7%) parents were concerned about costs related to ambulance transports 5. 23/106 (21.7%) other	1. Professional 2. Patient/relative
Keene (2015) Australia [85]	Reasons for not accepting transport (from fieldnotes): 1. Just wanted reassurance, assistance, advice or support/ referral 2. Symptoms had resolved prior to arrival or during assessment 3. Concern over ED waiting time/ED workload 4. Prior negative experience with a hospital 5. Personal reasons: (e.g. ‘I just didn’t want to go’. ‘I was embarrassed by all the fuss’	1. Patient/relative
Mikolaizak (2013) Australia [26]	Factors influencing transport decision: 1. refusal to travel 2. patient did not sustain an injury/only minor injuries 3. sufficient on-scene treatment 4.referral to GP	1. Patient/relative
Murphy-Jones (2016) UK [86]	3 main themes: 1. Patient wishes (insufficient care plans, nursing care staff insufficient knowledge of patients’ wishes, patients’ inability to express their wishes) 2. patients’ best interest (when patients were not considered to have the capacity for decision making, paramedics want to act in their best interest, factors used: diagnosis, comorbidities, quality of life, wishes and current condition, risks and benefits of hospitalization, concerns about care provision in some nursing homes 3. influence of others (nursing home staff, patients’ relatives and other paramedics)	1. Patient/relative 2. Healthcare process/system
O’Hara (2015) UK [87]	7 overarching system influences on decision making: 1. Increasing demand (of non-emergent cases) 2. Performance regime and priorities 3. Access to appropriate care options in case of non-conveyance to an ED 4. Disproportionate risk aversion: non-conveyance was perceived as a risk for both patient and paramedic 5. Beneficial impact of additional training on decision making competences 6. Communication and feedback to crews 7. Ambulance service resources	1. Healthcare process/system
Porter (2007) UK [88]	Influencing factors: 1. Patient autonomy 2. Opinion family/carers 3. Clinical need as assessed by crew members 4. Protection of themselves for the risk of litigation by crew members 5. Mental capacity of the patient to make a transport decision 6. Lacking skills or status of the crew member to be judging the mental capacity of the patient 7. Back-up of other professionals 8. Fear of a possible comeback if the non-conveyance decision turned out to be wrong	1. Patient/relative 2. Professional
Simpson (2014a) Australia [74]	6-item predictive model for non-conveyance odds (goodness-of-fit test indicated good model fit (8 DF, χ2 = 7.43, *p* = 0.49), factors associated with increased odds of a non-conveyance outcome. 1. 65–74 year 2. Lower response priority (90 min response time) 3. The presence of personal alarm 4. The absence of new injury/pain 5. Normal physiology 6. Change in usual level of function post fall	1. Patient/relative 2. Healthcare process/system
Snooks (2005) UK [89]	Influencing factors on ED conveyance: 1. Experience and intuition of the paramedic 2. Pragmatism: conveyance – the easy option 3. Patient/carer factors	1. Professional 2. Patient/relative
Stark (1990) USA [49]	Predictors for left at Scene Against Medical Advice: 1. Family present (β = −1.87, *p* = .001) 2. Disorientation (β = −1.04, *p* = .04) 3. Abnormal speech (β = −1.92, *p* = .05) 4. Police hold (β = −2.04, *p* = .03) 5. Alcohol use (β = 1.48, *p* = .006) 6. Treated hypoglycemia (β = 1.63, *p* = .05)	1. Patient/relative 2. Healthcare process/system
Stuhlmiller (2005) USA [51]	28/137 (20.4%) patients with whom the online medical control (OLMC) physician spoke during the encounter: 9/28 (32.1%) agreed to be transported, compared with nine (8.3%) of the 109 patients who did not speak to the OLMC physician (*p* = .001)	1. Supportive tools
Van der Pols (2011) Netherlands [77]	Motorcycle response vehicles with one ambulance nurse with additional training (*n* = 468) compared to regular ambulance (*n* = 1196): (1) treat and release 129/468 (27.6%) vs 149/1196 (12.5%) RR 2.21 (95%CI 1.80–2.73)	1. Professional
Vilke (2002) USA [78]	Patient reasons (*n* = 100) for patients to refuse transport: 1. 37/100 (37.0%) did not want transport and ED care 2. 23/100 (23.0%) concerned about the cost/coverage of ED 3. 19/100 (19.0%) paramedics implied no transport was needed 4. 17/100 (17.0%) concerned about the cost of the ambulance 5. 4/100 (4.0%) language barrier	1. Patient/relative
Zorab (1999) UK [80]	274/302 (90.7%) paramedics felt that a lack of health information of the patient had led to a less appropriate carepathway being selected, information that could have helped according to paramedics: 1. Resuscitation status (*n* = 233, 77.2%) 2. Current medication (*n* = 184, 60.9%) 3. Allergy information (*n* = 103, 34.1%) 4. Previous medical history (*n* = 262, 86.8%) 5. Patient’s normal parameters (*n* = 235, 77.8%) 6. End of life care choices (*n* = 221, 73.2%) 7. Information about implanted devices, e.g. pacemakers (*n* = 106, 35.1%) 8.Other, e.g. ECG, mental health records, blood and other test results (*n* = 38, 1.3%)	1. Professional

As for professional related factors, two studies described *professional competencies* needed to perform non-conveyance. These studies showed that additional training for ambulance professionals led to higher non-conveyance rates compared to ambulance professionals who received regular training [64, 77]. Besides competencies, other professional related factors are *weighing of patient risks and personal litigation risk* in case of a wrong non-conveyance decision [87, 88], *experience and intuition* of the ambulance professional [89], and *pragmatism* as conveyance being an easy option compared to non-conveyance [89].

For patient related factors, firstly the *health status of the patient* influenced the non-conveyance decision of the professional [26, 49, 65, 74, 78, 83, 85, 88]. Only three studies specified these physical conditions: the sufficiency of on-scene treatment [26], if problems/injuries have resolved pre-arrival or were only minor [26, 85], patient physiology [74], the absence of new pain or injury [74], and possible changes in usual level of functioning [74]. A second patient related factor is *refusal*. Refusal might be related to relatives thinking conveyance is not necessary [65], but also by patients concerns about costs of conveyance or ED care [65, 78], or the refusal reasons were not further specified [26, 85]. Thirdly, *patient wishes and the patients’ best interest* are factors that influence a conveyance decision [86].

Influencing factors related to the healthcare system are *access/referral to GP or alternative healthcare facility* in case of non-conveyance [26, 87]. To make appropriate conveyance or referral decisions, access to patient information is essential. One study [80] showed that 90.7% of the ambulance professionals felt that a lack of *patient information* leads to less appropriate care being selected. To make appropriate decisions, ambulance professionals gave high priority to previous medical history, patient’s usual vital signs and resuscitation status as patient information.

Finally, three studies showed that implementing online medical control *as supportive tool*, where a physician can be contacted by the pre-hospital professional, solely or in combination with a high risk card, increased conveyance rates for patients with high risk criteria or patients who refused conveyance [29, 51, 57].

## Discussion

This systematic review includes 67 articles that describe non-conveyance in ambulance care from patient safety and ambulance professional perspectives. Our results show that non-conveyance occurs in all types of EMS systems across the world, and that there is a wide variation in non-conveyance rates for general and specific patient populations. These variations might be caused by differences in patient populations (medical acuity and medical necessity to convey), and differences between EMS-systems in terms of triage systems, types of services, educational levels of ambulance professionals, and type of vehicles (conveying and non-conveying) [91–93]. Although non-conveyance in itself is a valid outcome of ambulance care [17], our results do not distinct between justified or unjustified non-conveyance. This can be a focus of future research.

Our review provides a first insight in characteristics of non-conveyed patients. Our results show that patients of all ages and both men and women are represented in the non-conveyance population. Non-conveyed patients most often had a neurological or trauma related complaint or condition. Vulnerable patients as children and elderly, and specific patient groups of people who had fallen or people with hypoglycaemia are relatively high represented in the non-conveyance population. Another subpopulation is patients who refuse care and/or conveyance. From our results it remains unknown what kinds of complaints or conditions these patients have from ICD-10 perspective, and what consequences their refusal has from patient-safety perspective.

Although the assessment of vital signs is an important aspect of the primary survey in ambulance care to make appropriate treatment and triage decisions [94], we found only three studies describing vital signs of non-conveyed patients. These studies show that roughly 15% of the non-conveyed patients have vital signs that deviate from limits. We do not know whether vital signs differ between conveyed and non-conveyed patients. Therefore future research should focus on a comparison of vital signs and follow-up outcomes between conveyed and non-conveyed patient groups. Furthermore, it remains unclear if abnormal vital signs were present in the medical history due to illness or medication use. Poor access to healthcare information systems by ambulance professionals is reported [80], this underlines the possible advantage of access to healthcare information systems in the chain of emergency care, and the accessibility of the general practitioner.

Results show that a significant amount of non-conveyed patient re-enters the (emergency) healthcare system. For instance, 6.1% of the patients re-enters the EMS-system <24 h after non-conveyance, and up to 19.0% of the patient visits an ED within 48 h after non-conveyance. From the patient-safety perspective it remains unclear whether these repeated EMS calls and ED visits are based on medical necessity, as it remained unclear in the data which complaints or conditions these patients had during this repeated access to emergency healthcare, and whether it was similar to the initial EMS contact. Furthermore, the studies did not describe whether the re-entry is based on professional referral or self-referral. Clinical practice could benefit from the development of valid quality indicators for patient safety in the chain of emergency care. These could measure systematically (un)justified re-entry of the emergency healthcare system and quality of care provided.

From the professional perspective, our results indicate that the non-conveyance decision-making process is multifactorial, with influences from the professional, the patient and his relatives, the healthcare system, and supportive tools. Our results do not give clear direction which additional competencies ambulance professionals need to make safe non-conveyance decisions, as only two studies describe positive effects of additional training. Studies not included in our review suggest that pre-hospital professionals with additional training on the conveyance decision, and on management of minor illness and injuries, are less likely to convey patients compared to regular ambulance staff [15, 95]. Initiatives to implement new competencies of pre-hospital professionals in EMS or possibly new professionals with additional competencies in clinical reasoning and conveyance decision-making should be explored and tested regarding patient safety.

As for supportive tools, our results show that there is a limited number of flowcharts, checklists or protocols available to guide non-conveyance decisions for general and specific patient populations. However, it remains unclear how these tools were developed and to what degree they are evidence-based. This urges the need to develop evidence-based supportive tools to guide non-conveyance decision-making for different patient groups. In order to do so, future research should be aimed at identifying factors to guide accurate non-conveyance decision making, to predict non-conveyance in the EMS dispatch phase through tailored triage criteria, or to predict follow-up outcomes such as mortality and re-enters in the emergency healthcare system. This with the aim to support professionals in their decision making and to enhance quality and safety in pre-hospital care.

### Limitations of included studies

As described in the result section, the quality of included studies varied. For the quantitative studies (Additional file 4: Appendix 3, Additional file 5: Appendix 4, Additional file 6: Appendix 5), the quality assessment criteria objective/aim, design, methods of subject/group selection, appropriateness of sample size, description analytical methods, and detailed reporting of results scored good quality. The moderate assessment criteria were description of subject characteristics, outcome definition, and the relationship between results and conclusion. The reporting of estimate of variance was poor, and due to design most studies could not be controlled for confounding. Within the qualitative studies (Additional file 7: Appendix 6) the quality assessment criteria objective/aim, design, connection to theoretical framework, data-collection and data-analysis scored good quality. The moderate assessment criteria were description of context, sampling strategy, and conclusion supported by results. Use of verification procedures and reflexivity of account were the two poor assessment criteria. Another limitation concerned the studies describing initial complaints and conditions. These studies used different types of classification systems, or systems were lacking. Therefore, we recommend to use one classification system, such as the ICD-10, in future research to enhance generizability and comparability of results.

### Study strengths and limitations

Despite the fact that this systematic review is the most complete and systematic analysis to date of non-conveyance in ambulance care, there are some limitations. A possible limitation is that our review did not cover the entire ambulance care process, as we focused on the phases after ambulance dispatch. Additional research should focus on the accuracy and predictive value of current EMS dispatch systems for non-conveyance decisions. Secondly, a meta-analyses was not feasible due to heterogeneity amongst studies. Another limitation concerns the quality assessment tools for quantitative and qualitative designs. A variety of these tools exist without a clear evidence-base. Strengths of our study concern the usage of Cochrane and PRISMA methods and tools to perform and report our research.

## Conclusion

This systematic review shows that non-conveyance occurs in all types of EMS systems across the world, and that a wide variation in non-conveyance rates for general and specific patient populations exists. Patients in the non-conveyance population present themselves with a variety of initial complaints and conditions, although initial complaints or conditions related to trauma and neurology, and vulnerable patients groups such as children, elderly and patients with hypoglycaemia, are well represented. Nevertheless, further insight in characteristics of the non-conveyance population is needed. From patient safety perspective it turns out that a proportion of non-conveyed patients re-enters the emergency healthcare system within one or 2 days after non-conveyance. Why these patients re-enter the emergency healthcare system, and what outcomes these patients have remains unclear. For ambulance professionals the non-conveyance decision-making process is complex and multifactorial, with influences from the professional, the patient and his relatives, the healthcare system (referral or access to general practitioner) and supportive tools. Competencies needed to perform non-conveyance are marginally described, this should be priority in future research. Despite the fact that a limited amount of supportive tools is available for general and specific non-conveyance populations, there is a need to develop evidence-based guidelines and protocols to guide non-conveyance decision-making.

## Additional files

Additional file 1: Prisma 2009 checklist (DOC 63 kb)

Additional file 2: Appendix 1 Search strategies (DOCX 16 kb)

Additional file 3: Appendix 2 Reasons full text exclusion (*n* = 67 articles) (DOCX 24 kb)

Additional file 4: Appendix 3 Quality of systematic reviews (*n* = 2) (DOC 305 kb)

Additional file 5: Appendix 4 Quality of experimental studies (*n* = 4) (DOC 425 kb)

Additional file 6: Appendix 5 Quality of quantitative studies (*n* = 53) (DOC 5545 kb)

Additional file 7: Appendix 6 Quality of qualitative studies (*n* = 8) (DOC 910 kb)

Additional file 8: Appendix 7 Non-conveyance rates and patient characteristics (DOCX 48 kb)

Additional file 9: Appendix 8 Guidelines/protocols/triage criteria (DOCX 29 kb)
