# Supplementary material for: A patient-safety and professional perspective on non-conveyance in ambulance care: a systematic review
Source: Scand J Trauma Resusc Emerg Med. 2017 Jul 17;25:71. doi: 10.1186/s13049-017-0409-6 (PMC5513207; doi:10.1186/s13049-017-0409-6)
Supplement: Supplementary file 2 — Appendix 1 Search strategies (DOCX 16 kb) [file 13049_2017_409_MOESM2_ESM.docx]

**Pubmed**

Emergency Medical Services[Mesh:NoExp] OR Emergency Medical Service*[tiab] OR Medical Emergency Service*[tiab] OR EMS[tiab] OR emergency care[tiab] OR Transportation of patients[Mesh] OR ambulance*[tiab] OR emergency mobile unit*[tiab] OR mobile emergency unit*[tiab] OR Emergency Medical Technicians[mesh] OR Emergency Medical Technician*[tiab] OR EMT[tiab] OR paramedic*[tiab] OR emergency worker*[tiab] OR  emergency provider*[tiab] OR emergency staff[tiab] OR emergency practitioner*[tiab] OR emergency nurs*[tiab] OR Rescue worker*[tiab] OR Rescue staff[tiab] OR Rescue nurs*[tiab] OR recovery worker*[tiab] OR recovery provider*[tiab] OR recovery staff[tiab] OR recovery practitioner*[tiab] OR recovery nurs*[tiab] OR EMS worker*[tiab] OR EMS provider*[tiab] OR EMS staff[tiab] OR EMS practitioner*[tiab] OR EMS nurs*[tiab]

AND

(Health Services Misuse[Mesh] OR transport need*[tiab] OR transportation need*[tiab] OR transport decision*[tiab] OR transportation decision*[tiab] OR non transport*[tiab] OR none transport*[tiab] OR non convey*[tiab] OR none convey*[tiab] OR discharge at the scene[tiab] OR treat and release[tiab] OR prehospital discharge[tiab])

NOT

medline [SB]

**Medline (EBSCO)**

((MH "Emergency Medical Services" OR (TI (Emergency N2 Medical N2 Service*) OR EMS OR (Emergency N2 Health N2 Services) OR ((emergency OR prehospital* or “pre hospital”) N2 care)) OR (AB (Emergency N2 Medical N2 Service*) OR EMS OR (Emergency N2 Health N2 Services) OR ((emergency OR prehospital* or “pre hospital”) N2 care)) OR (SU (Emergency N2 Medical N2 Service*) OR EMS OR (Emergency N2 Health N2 Services) OR (emergency OR prehospital* or “pre hospital”) N2 care))) OR ((MH "Transportation of Patients+") OR (TI (Ambulance* OR (emergency N2 (vehicle* OR car) OR (Emergency N2 Mobile N2 Unit*))) OR (AB (Ambulance* OR (emergency N2 (vehicle* OR car)OR (Emergency N2 Mobile N2 Unit*))) OR (SU (Ambulance* OR (emergency N2 (vehicle* OR car)) OR (Emergency N2 Mobile N2 Unit*)))) OR ((MH "Emergency Medical Technicians") OR ((TI EMT OR (emergency N2 medical N2 technician*) OR paramedic* OR ((emergency OR Rescue OR recovery OR EMS) N2 (worker* OR personnel OR provider* OR staff OR practitioner* OR nurs*))) OR ((AB EMT OR (emergency N2 medical N2 technician*) OR paramedic* OR ((emergency OR Rescue OR recovery OR EMS) N2 (worker* OR personnel OR provider* OR staff OR practitioner* OR nurs*))) OR ((SU EMT OR (emergency N2 medical N2 technician*) OR paramedic* OR ((emergency OR Rescue OR recovery OR EMS) N2 (worker* OR personnel OR provider* OR staff OR practitioner* OR nurs*))))))

AND

((MH "Health Services Misuse+") OR ((TI ((Transport* OR convey*) N2 (need* OR decision* OR misus* OR reduc*)) OR ((Transport* OR convey*) N1 (non OR none)) OR nontransport* OR nonetransport* OR nonconvey* OR noneconvey*) OR (AB ((Transport* OR convey*) N2 (need* OR decision* OR misus* OR reduc*)) OR ((Transport* OR convey*) N1 (non OR none)) OR nontransport* OR nonetransport* OR nonconvey* OR noneconvey*) OR (SU ((Transport* OR convey*) N2 (need* OR decision* OR misus* OR reduc*)) OR ((Transport* OR convey*) N1 (non OR none)) OR nontransport* OR nonetransport* OR nonconvey* OR noneconvey*)) OR (((MH “triage”) OR (AB triag* OR undertriag* OR overtriag*) OR (TI triage* OR undertriag* OR overtriag*) OR (SU triag* OR undertriag* OR overtriag*)) AND ((AB (Transport* OR convey*)) OR (TI (Transport* OR convey*)) OR (SU (Transport* OR convey*)))) OR ((TI ((discharg* N2 (scene* OR site* OR prehospital OR “out of hospital” OR “pre hospital”)) OR (Treat* N2 releas*))) OR (AB ((discharg* N2 (scene* OR site* OR prehospital OR “out of hospital” OR “pre hospital”)) OR (Treat* N2 releas*))) OR (SU ((discharg* N2 (scene* OR site* OR prehospital OR “out of hospital” OR “pre hospital”)) OR (Treat* N2 releas*)))))

**Cinahl**

((MH "Emergency Medical Services" OR (TI (Emergency N2 Medical N2 Service*) OR EMS OR (Emergency N2 Health N2 Services) OR ((emergency OR prehospital* or “pre hospital”) N2 care)) OR (AB (Emergency N2 Medical N2 Service*) OR EMS OR (Emergency N2 Health N2 Services) OR ((emergency OR prehospital* or “pre hospital”) N2 care)) OR (SU (Emergency N2 Medical N2 Service*) OR EMS OR (Emergency N2 Health N2 Services) OR (emergency OR prehospital* or “pre hospital”) N2 care))) OR ((MH "Transportation of Patients+") OR (TI (Ambulance* OR (emergency N2 (vehicle* OR car) OR (Emergency N2 Mobile N2 Unit*))) OR (AB (Ambulance* OR (emergency N2 (vehicle* OR car)OR (Emergency N2 Mobile N2 Unit*))) OR (SU (Ambulance* OR (emergency N2 (vehicle* OR car)) OR (Emergency N2 Mobile N2 Unit*)))) OR ((MH "Emergency Medical Technicians") OR (MH “[Emergency Medical Technician Attitudes](javascript:XslPostBack('ctl00$ctl00$MainContentArea$MainContentArea$ctrlResults','meshDetail','index%7C2%24term%7CEmergency%20Medical%20Technician%20Attitudes%24cmd%7CmeshDetail');)”) OR ((TI EMT OR (emergency N2 medical N2 technician*) OR paramedic* OR ((emergency OR Rescue OR recovery OR EMS) N2 (worker* OR personnel OR provider* OR staff OR practitioner* OR nurs*))) OR ((AB EMT OR (emergency N2 medical N2 technician*) OR paramedic* OR ((emergency OR Rescue OR recovery OR EMS) N2 (worker* OR personnel OR provider* OR staff OR practitioner* OR nurs*))) OR ((SU EMT OR (emergency N2 medical N2 technician*) OR paramedic* OR ((emergency OR Rescue OR recovery OR EMS) N2 (worker* OR personnel OR provider* OR staff OR practitioner* OR nurs*))))))

AND

((MH "Health Services Misuse+") OR ((TI ((Transport* OR convey*) N2 (need* OR decision* OR misus* OR reduc*)) OR ((Transport* OR convey*) N1 (non OR none)) OR nontransport* OR nonetransport* OR nonconvey* OR noneconvey*) OR (AB ((Transport* OR convey*) N2 (need* OR decision* OR misus* OR reduc*)) OR ((Transport* OR convey*) N1 (non OR none)) OR nontransport* OR nonetransport* OR nonconvey* OR noneconvey*) OR (SU ((Transport* OR convey*) N2 (need* OR decision* OR misus* OR reduc*)) OR ((Transport* OR convey*) N1 (non OR none)) OR nontransport* OR nonetransport* OR nonconvey* OR noneconvey*)) OR (((MH “triage”) OR (AB triag* OR undertriag* OR overtriag*) OR (TI triage* OR undertriag* OR overtriag*) OR (SU triag* OR undertriag* OR overtriag*)) AND ((AB (Transport* OR convey*)) OR (TI (Transport* OR convey*)) OR (SU (Transport* OR convey*)))) OR ((TI ((discharg* N2 (scene* OR site* OR prehospital OR “out of hospital” OR “pre hospital”)) OR (Treat* N2 releas*))) OR (AB ((discharg* N2 (scene* OR site* OR prehospital OR “out of hospital” OR “pre hospital”)) OR (Treat* N2 releas*))) OR (SU ((discharg* N2 (scene* OR site* OR prehospital OR “out of hospital” OR “pre hospital”)) OR (Treat* N2 releas*)))))

**EMBASE**

((emergency health service/ OR EXP Emergency medicine/ OR emergency care/ OR ((Emergency adj3 Medical adj3 Service*) OR EMS OR (Emergency adj3 Health adj3 Service*) OR (emergency adj3 care)).ti,ab,kw.) OR (EXP Patient transport/ OR EXP Ambulances/ OR (Ambulance* OR (Emergency adj3 ((Mobile adj3 Unit*) OR car OR cars OR vehicle*))).ti,ab,kw.) OR (Paramedical personnel/ OR Rescue personell/ OR Rescue personell attitude/ OR Rescue work/ OR Emergency nursing/ OR Emergency physician/ OR (EMT OR ((emergency OR first) ADJ1 responder) OR (emergency ADJ2 medical ADJ2 technician*) OR paramedic* OR ((emergency OR Rescue OR recovery OR EMS) ADJ2 (worker* OR personnel OR provider* OR staff OR practitioner* OR nurs* OR team*))).ti,ab,kw.))

AND

(((((Transport* OR convey*) ADJ3 (need* OR decision* OR none OR non OR misus* OR reduc*)) OR nontransport* OR nonetransport* OR nonconvey* OR noneconvey*).ti,ab,kw.) OR (((triag* OR overtriag* OR undertriag*) AND (Transport* OR convey*)).ti,ab,kw.) OR ((((discharg* OR (Treat* adj2 releas*)) adj2 (scene* OR site* OR prehospital OR (out of hospital) OR (pre adj1 hospital)))).ti,ab,kw.))
