# Supplementary material for: A patient-safety and professional perspective on non-conveyance in ambulance care: a systematic review
Source: Scand J Trauma Resusc Emerg Med. 2017 Jul 17;25:71. doi: 10.1186/s13049-017-0409-6 (PMC5513207; doi:10.1186/s13049-017-0409-6)
Supplement: Supplementary file 3 — Appendix 2 Reasons full text exclusion (n = 67 articles) (DOCX 24 kb) [file 13049_2017_409_MOESM3_ESM.docx]

| **Appendix 2 – Reason full text exclusion (n=67 articles)** | | |
| --- | --- | --- |
| Reason | N | References |
| Other design | 28 | [1-28] |
| No non-conveyance | 25 | [29-53] |
| Other non-conveyance topic | 9 | [54-62] |
| Other setting | 2 | [63, 64] |
| Other language (Chinese) | 1 | [65] |
| Article could not be retrieved full text | 1 | [66] |
| Suspected plagiarism* | 1 | [67] |
| *Authors and journal are contacted |  |  |

References

1.Brainard J. and Alefounder H: **Ambulance trust initiatives to reduce conveyances to emergency departments.** *European Journal of Emergency Medicine* 2016, **23:**235-236.

2.Close JCT, Halter M, Elrick A, Brain G, Swift CG: **Falls in the older population: a pilot study to assess those attended by London ambulance service but not taken to A&E.** *Age Ageing* 2002, **31:**488.

3.Colver KA. **Ambulance Service Treat and Refer guidelines: a qualitative investigation into the use of Treat and Refer Guidelines by ambulance clinicians.** *Ambulance Service Treat & Refer Guidelines: A Qualitative Investigation Into the Use of Treat & Refer Guidelines by Ambulance Clinicians* 2012,N.PAG p-N.PAG p 1p.

4.Colver K, Abhyankar P, Niven C: **Ambulance service treat and refer guidelines: a qualitaive investigation into the use of treat and refer guidelines by ambulance clinicians.** *Emerg.Med.J.* 2015, **32:**e10-e10 1p.

5.Ehrlich BA: **Inappropriate use of ambulances.** *N.Engl.J.Med.* 1984, **311:**801.

6.Emerman CL: **Trauma triage: where do we go from here?** *Academic Emergency Medicine: Official Journal Of The Society For Academic Emergency Medicine* 1995, **2:**1025.

7.Essam N, Windle K, Mullineaux D, Knowles S, Gray J, Siriwardena N: **Modified early warning scores (mews) to support ambulance clinicians' decisions to transport or treat at home.** *Emerg.Med.J.* 2015, **32:**e1-e1 1p.

8.Holroyd B, Shalit M, Kallsen G, Culhane D, Knopp R: **Prehospital patients refusing care.** *Ann.Emerg.Med.* 1988, **17:**957.

9.Hunt R.C., Sasser S., Bailey B: **Triage to transport. A case for national field triage guidelines.** *JEMS : a journal of emergency medical services* 2011, **36:**14.

10.Krohmer JR: **Appropriate emergency medical services transport.** *Academic Emergency Medicine: Official Journal Of The Society For Academic Emergency Medicine* 1999, **6:**5.

11.Mann N.C., Schmidt T.A., Cone DC: **Defining Research Criteria to Characterize Medical Necessity in Emergency Medical Services: A Consensus among Experts at the Neely Conference.** *Prehospital Emergency Care* 2004, **8:**138-153.

12.Mikolaizak AS, Simpson PM, Tiedemann A, Lord SR, Caplan GA, Bendall JC, Howard K, Close JCT: **Intervention to prevent further falls in older people who call an ambulance as a result of a fall: a protocol for the iPREFER randomised controlled trial.** *BMC Health Services Research* 2013, **13:**360.

13.Millin M.G., Brown L.H., Schwartz B: **EMS provider determinations of necessity for transport and reimbursement for EMS response, medical care, and transport: combined resource document for the National Association of EMS Physicians position statements.** *Prehospital emergency care : official journal of the National Association of EMS Physicians and the National Association of State EMS Directors* 2011, **15:**562-569.

14.Neely KW: **Ambulance transports: what are the alternatives?** *Academic Emergency Medicine: Official Journal Of The Society For Academic Emergency Medicine* 1997, **4:**1103.

15.Oâ€™Hara R, Johnson M, Hirst E, Weyman A, Shaw D, Mortimer P, Newman C, Storey M, Turner J, Mason S, Quinn T, Shewan J, Siriwardena AN: **A qualitative study of decision-making and safety in ambulance service transitions.**2014,

16.O'Hara R, Johnson M, Hirst E, Weyman A, Shaw D, Mortimer P, Newman C, Storey M, Turner J, Mason S, Quinn T, Shewan J, Siriwardena AN: **Decision making and safety in ambulance service transitions.** *Emerg.Med.J.* 2015, **32:**e2-e2 1p.

17.O'Hara R, Hirst E, Johnson M, Newman C, Shaw D, Turner J: **Service user perspectives on patient safety in the ambulance service.** *Emerg.Med.J.* 2015, **32:**e12-e12 1p.

18.Rea T, Plorde M, Hauswald M, Silvestri S, Rothrock SG, Pagane JR: **Diagnosis and triage by EMS professionals...Silvestri S, Rothrock SG, Kennedy D, Ladde J, Bryant M, Pagane J. Can paramedics accurately identify patients who do not require emergency department care? Prehosp Emerg Care. 2002;6:387-90... Hauswald M. Can paramedics safely decide which patients do not need ambulance transport or emergency care? Prehosp Emerg Care. 2002;6:383-6.** *PREHOSPITAL EMERG CARE* 2003, **7:**295-297 3p.

19.Roberts K and Smith A: **Outcome of diabetic patients treated in the prehospital arena after a hypoglycaemic episode, and an exploration of treat and release protocols: a review of the literature.** *Emerg.Med.J.* 2003,274-276 3p.

20.Sanaei-Zadeh H: **Is discharge-on-scene policy really safe after naloxone reversal of acute long-acting opioid toxicity?...Resuscitation. 2011 Nov;82(11):1414-8.** *Resuscitation* 2013, **84:**e15-e15 1p.

21.Snooks HA, Kingston MR, Anthony RE, Russell IT: **New models of emergency prehospital care that avoid unnecessary conveyance to emergency department: translation of research evidence into practice?** *THESCIENTIFICWORLDJOURNAL* 2013,182102-182102 1p.

22.Taigman M and Fowler R: **Who gets to ride? Deciding when it's appropriate not to transport patients to the ED.** *JEMS* 2004, **29:**46-55 8p.

23.Thompson F, Jacob R, Watson P, Holland A, Clare I: **05â€…Just saying â€˜Noâ€™? factors associated with patientsâ€™ non-acceptance of ambulance transport against cliniciansâ€™ advice.** *Emerg.Med.J.* 2011, **28:**e1-e1 1p.

24.Anonymous **EMS systems cut unnecessary use of emergency resources: communities model different approaches to handing sic] low-acuity calls.** *EMS INSIDER* 2007, **34:**1-7 3p.

25.Anonymous **Get at root causes to reduce risk: patient surveys can be invaluable source.** *ED MANAGE* 2009, **21:**112-113 2p.

26.Anonymous **Services seek to cut unnecessary ambulance runs: cities target low-acuity patients & frequent users.** *EMS INSIDER* 2009, **36:**1-5 3p.

27.Anonymous **County paramedics provide treat and release.** *EMS INSIDER* 1998, **25:**7-8 2p.

28.Anonymous **Emergency Medical Service â€œTreat and Releaseâ€ Protocols: A Review of Clinical and Cost-Effectiveness, Safety, and Guidelines.**2014,

29.Billittier A.J., Lerner E.B., Moscati R.M., Young G: **Triage, transportation, and destination decisions by out-of-hospital emergency care providers.** *Prehospital and disaster medicine : the official journal of the National Association of EMS Physicians and the World Association for Emergency and Disaster Medicine in association with the Acute Care Foundation* 1998, **13:**22-27.

30.Booker MJ, Simmonds RL, Purdy S: **Patients who call emergency ambulances for primary care problems: a qualitative study of the decision-making process.** *Emerg.Med.J.* 2014, **31:**448-452 5p.

31.Booker MJ, Shaw ARG, Purdy S: **Why do patients with 'primary care sensitive' problems access ambulance services? A systematic mapping review of the literature.** *BMJ Open* 2015, **5:**e007726.

32.Brown L.H., Hubble M.W., Cone D.C., Millin M.G., Schwartz B., Patterson P.D., Greenberg B., Richards ME: **Paramedic determinations of medical necessity: A meta-analysis.** *Prehospital Emergency Care* 2009, **13:**516-527.

33.CamassoRichardson K., Wilde J.A., Petrack EM: **Medically unnecessary pediatric ambulance transports: A medical taxi service?.** *Acad.Emerg.Med.* 1997, **4:**1137-1141.

34.CarlstrÃ¶m E and FredÃ©n L: **The first single responders in Sweden - Evaluation of a pre-hospital single staffed unit.** *International Emergency Nursing* 2016,

35.Challen K. and Walter D: **Physiological scoring: an aid to emergency medical services transport decisions?.** *Prehospital and disaster medicine : the official journal of the National Association of EMS Physicians and the World Association for Emergency and Disaster Medicine in association with the Acute Care Foundation* 2010, **25:**320-323.

36.Chu KH, Gregor MA, Maio RF, Hill EM, Swor RA: **Derivation and validation of criteria for determining the appropriateness of nonemergency ambulance transports.** *PREHOSPITAL EMERG CARE* 1997, **1:**219-226 8p.

37.Clark M.J. and FitzGerald G: **Older people's use of ambulance services: A population based analysis.** *Journal of Accident and Emergency Medicine* 1999, **16:**108-111.

38.Clesham K., Mason S., Gray J., Walters S., Cooke V: **Can emergency medical service staff predict the disposition of patients they are transporting?.** *Emergency Medicine Journal* 2008, **25:**691-694.

39.Coats TJ, Wilson AW, Cross FW: **On-scene medical decision making and overtriage.** *Br.J.Surg.* 1993, **80:**1291.

40.Dunne R.B., Compton S., Welch R.D., Zalenski R.J., Bock BF: **Prehospital on-site triaging.** *Prehospital Emergency Care* 2003, **7:**85-88.

41.Durant E. and Fahimi J: **Factors associated with ambulance use among patients with low-acuity conditions.** *Prehospital emergency care : official journal of the National Association of EMS Physicians and the National Association of State EMS Directors* 2012, **16:**329-337.

42.Finn JC, Fatovich DM, Arendts G, Mountain D, Tohira H, Williams TA, Sprivulis P, Celenza A, Ahern T, Bremner AP, Cameron P, Borland ML, Rogers IR, Jacobs IG: **Evidence-based paramedic models of care to reduce unnecessary emergency department attendance - feasibility and safety.** *BMC EMERG MED* 2013, **13:**13-13 1p.

43.Fitzharris M, Stevenson M, Middleton P, Sinclair G: **Adherence with the pre-hospital triage protocol in the transport of injured patients in an urban setting.** *Injury* 2012, **43:**1368-1376 9p.

44.Gibson G: **Measures of emergency ambulance effectiveness: unmet needs and inappropriate use.** *Journal of the American College of Emergency Physicians and the Univ.Ass.for Emergency Med.Services* 1977, **6:**389-392.

45.Gratton M.C., Ellison S.R., Hunt J., Ma OJ: **Prospective determination of medical necessity for ambulance transport by paramedics.** *Prehospital Emergency Care* 2003, **7:**466-469.

46.Hauswald M: **Can paramedics safely decide which patients do not need ambulance transport or emergency department care?** *PREHOSPITAL EMERG CARE* 2002, **6:**383-386 4p.

47.HjÃ¤lte L, Suserud B, Herlitz J, Karlberg I: **Why are people without medical needs transported by ambulance? A study of indications for pre-hospital care.** *European Journal Of Emergency Medicine: Official Journal Of The European Society For Emergency Medicine* 2007, **14:**151.

48.Jones CMC, Cushman JT, Lerner EB, Fisher SG, Seplaki CL, Veazie PJ, Wasserman EB, Dozier A, Shah MN: **Prehospital Trauma Triage Decision-making: A Model of What Happens between the 9-1-1 Call and the Hospital.** *PREHOSPITAL EMERG CARE* 2016, **20:**6-14 9p.

49.Krumperman K., Weiss S., Fullerton L: **Two types of prehospital systems interventions that triage low-acuity patients to alternative sites of care.** *South.Med.J.* 2015, **108:**381-386.

50.Mierek C., Nacca N., Scott J.M., Wojcik S.M., D'Agostino J., Dougher K., Gill H., Maheux S., Grant W.D., Rodriguez E: **View from the door: Making pediatric transport decisions based on first impressions.** *JEMS : a journal of emergency medical services* 2010, **35:**68-69, 71, 73, 75, 77, 79, 81.

51.Newton M, Tunn E, Moses I, Ratcliffe D, Mackway-Jones K: **Clinical navigation for beginners: the clinical utility and safety of the Paramedic Pathfinder.** *Emerg.Med.J.* 2014, **31:**e29-e34 6p.

52.Phillips S.E., Gaskin P.S., Byer D., Cadogan W.L., Brathwaite A., Nielsen AL: **The Barbados emergency ambulance service: High frequency of nontransported calls.** *Emergency Medicine International* 2012, **2012:**Arte Number: 659392. ate of Pubaton: 2012.

53.Pointer J.E., Levitt M.A., Young J.C., Promes S.B., Messana B.J., Ader MEJ: **Can paramedics using guidelines accurately triage patients?.** *Ann.Emerg.Med.* 2001, **38:**268-277.

54.Grange J.T., Baumann G.W., Vaezazizi R: **On-site physicians reduce ambulance transports at mass gatherings.** *Prehospital Emergency Care* 2003, **7:**322-326.

55.Gray J.T. and Wardrope J: **Introduction of non-transport guidelines into an ambulance service: A retrospective review.** *Emergency Medicine Journal* 2007, **24:**727-729.

56.Feldman MJ, Lukins JL, Verbeek PR, Burgess RJ, Schwartz B: **Use of treat-and-release medical directives for paramedics at a mass gathering.** *PREHOSPITAL EMERG CARE* 2005, **9:**213-217 5p.

57.Munjal KG, Shastry S, Loo GT, Reid D, Grudzen C, Shah MN, Chapin HH, First B, Sirirungruang S, Alpert E, Chason K, Richardson LD: **Patient Perspectives on EMS Alternate Destination Models.** *Prehospital Emergency Care: Official Journal Of The National Association Of EMS Physicians And The National Association Of State EMS Directors* 2016,1.

58.Porter A., Shooks H., Youren A., Gaze S., Whitfield R., Rapport F., Woollard M: **"Covering our backs": Ambulance crews' attitudes towards clinical documentation when emergency (999) patients are not conveyed to hospital.** *Emergency Medicine Journal* 2008, **25:**292-295.

59.Rostykus P, Kennel J, Adair K, Fillinger M, Palmberg R, Quinn A, Ripley J, Daya M: **Variability in the Treatment of Prehospital Hypoglycemia: A Structured Review of EMS Protocols in the United States.** *Prehosp.Emerg.Care* 2016,1-7.

60.Snooks H, Kearsley N, Dale J, Halter M: **New models of care for 999 callers with conditions that are neither life threatening nor serious: results of a national survey.** *PREHOSPITAL IMMEDIATE CARE* 2000, **4:**180-182 3p.

61.SoaresOliveira M., Egipto P., Costa I., CunhaRibeiro LM: **Emergency motorcycle: has it a place in a medical emergency system?.** *Am.J.Emerg.Med.* 2007, **25:**620-622.

62.Evans R., McGovern R., Birch J., NewburyBirch D: **Which extended paramedic skills are making an impact in emergency care and can be related to the UK paramedic system? A systematic review of the literature.** *Emergency Medicine Journal* 2014, **31:**594-603.

63.Keller G.B., Lanese R.R., Keller MD: **Predicting EMS dispatch, transport, and admissions decisions with the emergency message.** *Emerg.Health Serv.Q.* 1981, **1:**35-46.

64.Manoguerra A.S., Erdman A.R., Woolf A.D., Chyka P.A., Martin Caravati E., Scharman E.J., Booze L.L., Christianson G., Nelson L.S., Cobaugh D.J., Troutman WG: **Valproic acid poisoning: An evidence-based consensus guideline for out-of-hospital management.** *Clin.Toxicol.* 2008, **46:**661-676.

65.Chi C.H., Tsai M.C., Yen Y.L., Ye Y.J., Lin S.M., Wu MH: **Ambulance utilization in Tainan: Analysis of emergency ambulance missions in urban and rural areas.** *Chin.J.Public Health* 1997, **16:**177-184.

66.Anonymous **Examining system abuse...'Can paramedics accurately identify patients who do not require emergency department care?'.** *Emerg.Med.Serv.* 2003, **32:**12-12 1p.

67.Pandey A. and Ranjan R: **Emergency (108) calls to the ambulance service in the state of gujarat (India) that do not result in the patient being transported to hospital: An epidemiological study.** *Journal of Clinical and Diagnostic Research* 2009, **3:**1519-1522.
