# Supplementary material for: A patient-safety and professional perspective on non-conveyance in ambulance care: a systematic review
Source: Scand J Trauma Resusc Emerg Med. 2017 Jul 17;25:71. doi: 10.1186/s13049-017-0409-6 (PMC5513207; doi:10.1186/s13049-017-0409-6)
Supplement: Supplementary file 4 — Appendix 3 Quality of systematic reviews (n = 2) (DOC 305 kb) [file 13049_2017_409_MOESM4_ESM.doc]

| **Appendix 3 - Quality of systematic reviews (n=2)**  **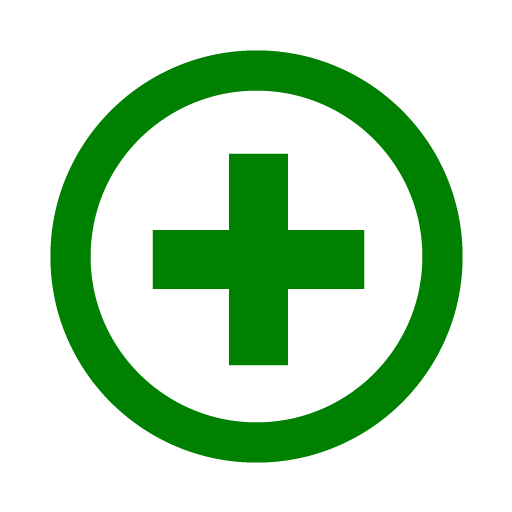 yes 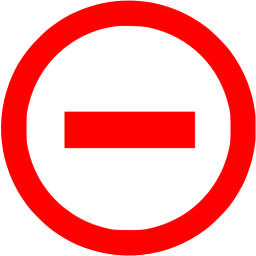 no, 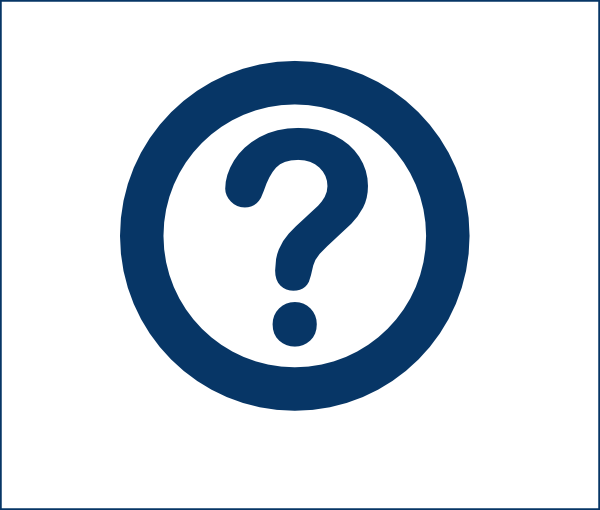 unknown** | | | | | | | | | | | | |
| --- | --- | --- | --- | --- | --- | --- | --- | --- | --- | --- | --- | --- |
| **First author (Year) [ref]** | **Was an 'a priori' design provided?** | **Was there duplicate study selection and data extraction?** | **Was a comprehensive literature search performed?** | **Was the status of publication (i.e. grey literature) used as an inclusion criterion?** | **Was a list of studies (included and excluded) provided?** | **Were the characteristics of the included studies provided?** | **Was the scientific quality of the included studies assessed and documented?** | **Was the scientific quality of the included studies used appropriately in formulating conclusions?** | **Were the methods used to combine the findings of studies appropriate?** | **Was the likelihood of publication bias assessed?** | **Was the conflict of interest included?** | **AMSTAR score** |
| Mikolaizak (2013) [26] | 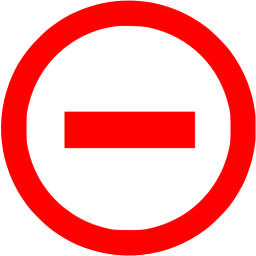 | 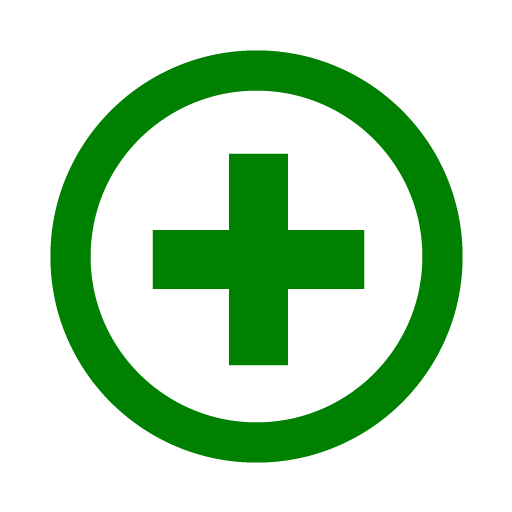 | 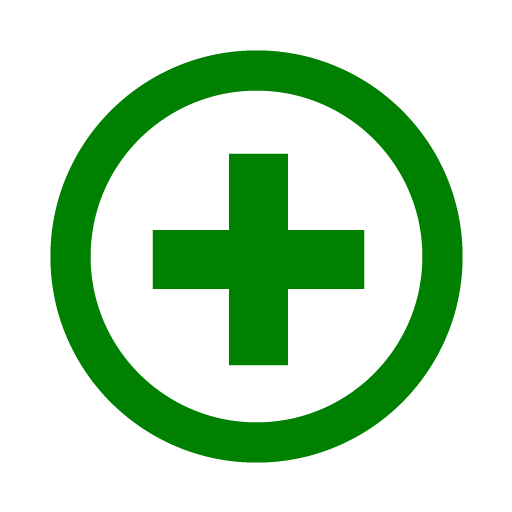 | 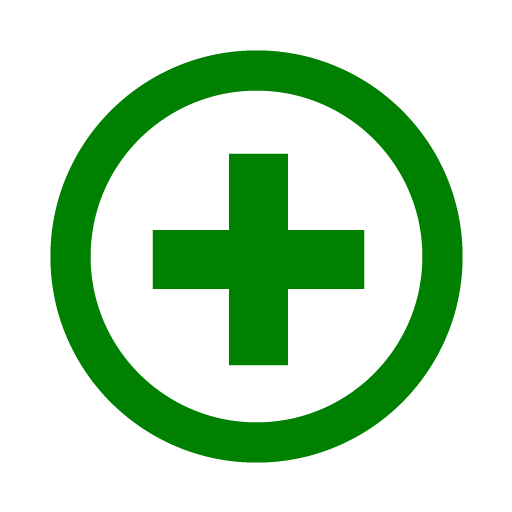 | 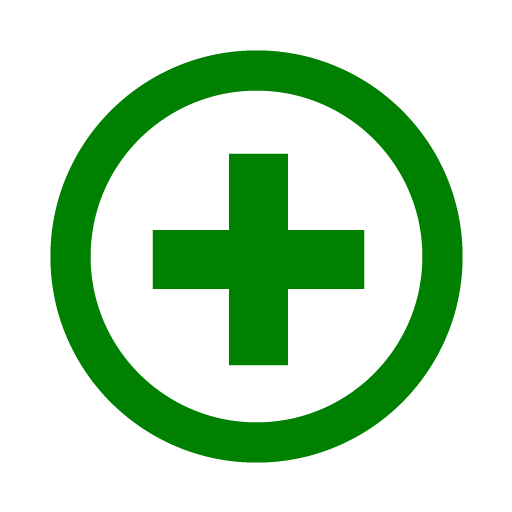 | 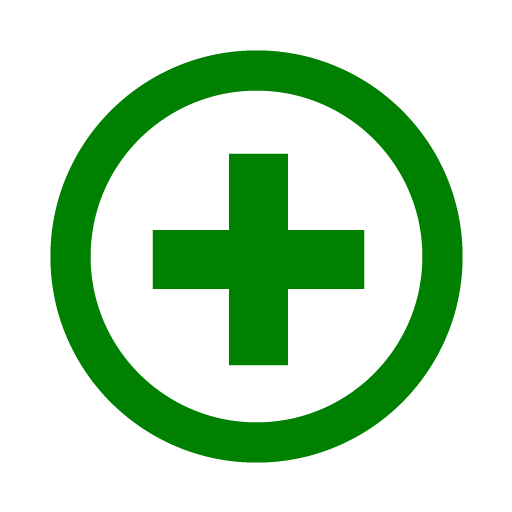 | 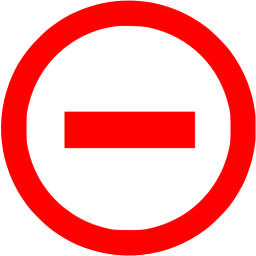 | 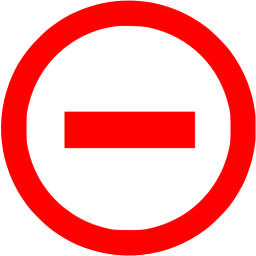 | 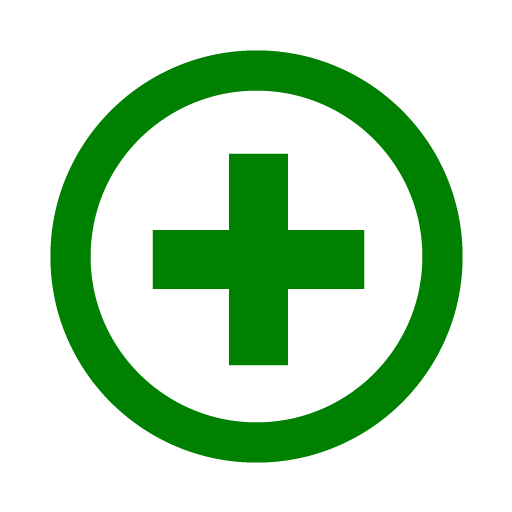 | 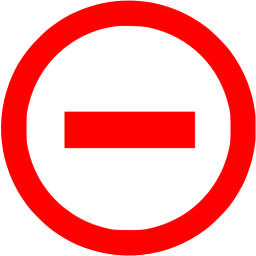 | 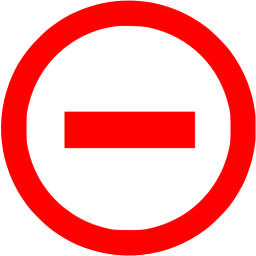 | 6 |
| Snooks (2004b) [10] | 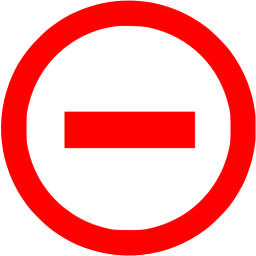 | 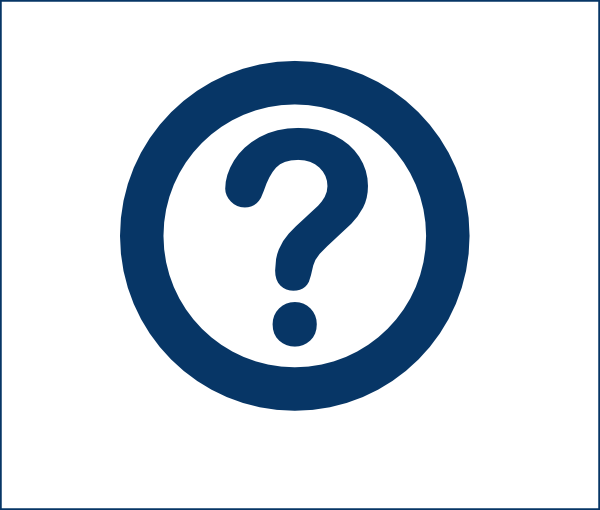 | 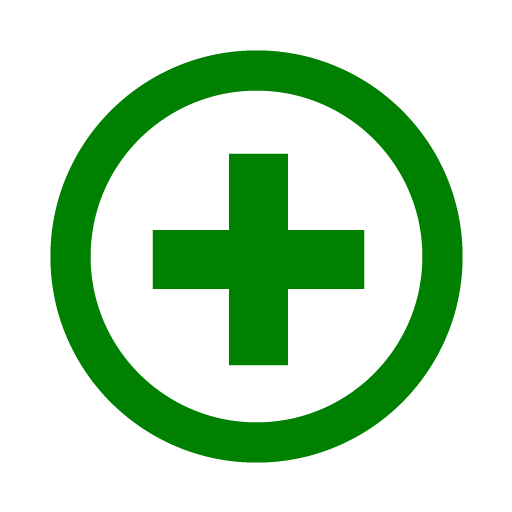 | 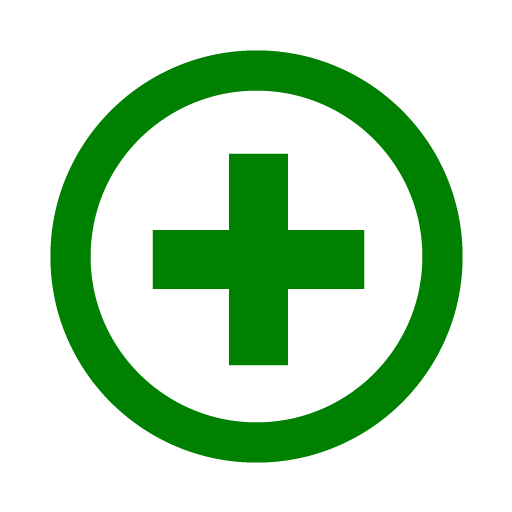 | 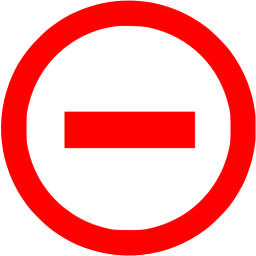 | 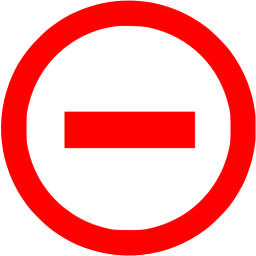 | 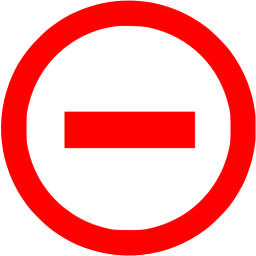 | 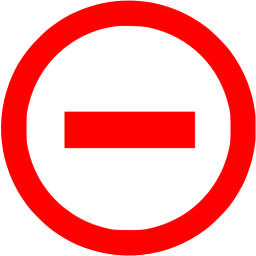 | 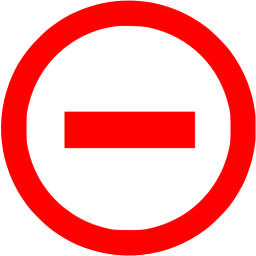 | 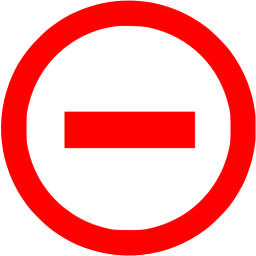 | 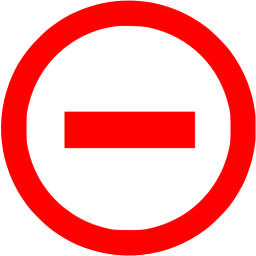 | 2 |
