# Supplementary material for: A patient-safety and professional perspective on non-conveyance in ambulance care: a systematic review
Source: Scand J Trauma Resusc Emerg Med. 2017 Jul 17;25:71. doi: 10.1186/s13049-017-0409-6 (PMC5513207; doi:10.1186/s13049-017-0409-6)
Supplement: Supplementary file 5 — Appendix 4 Quality of experimental studies (n = 4) (DOC 425 kb) [file 13049_2017_409_MOESM5_ESM.doc]

| **Appendix 4 - Quality of experimental studies (n=4)**  **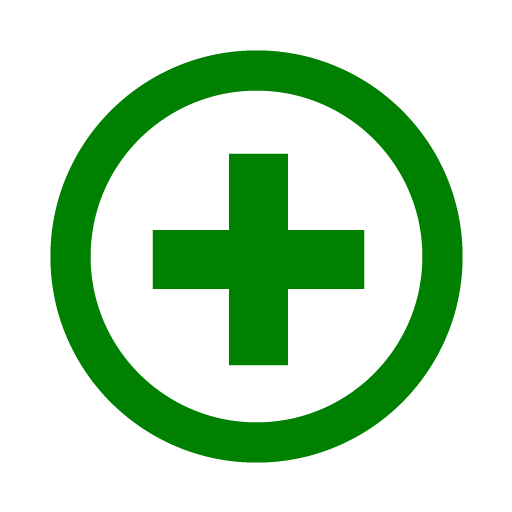 Low risk/Yes, 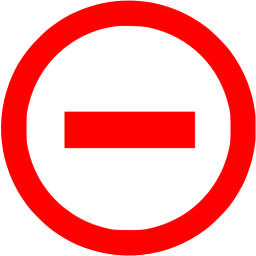 high risk/No, 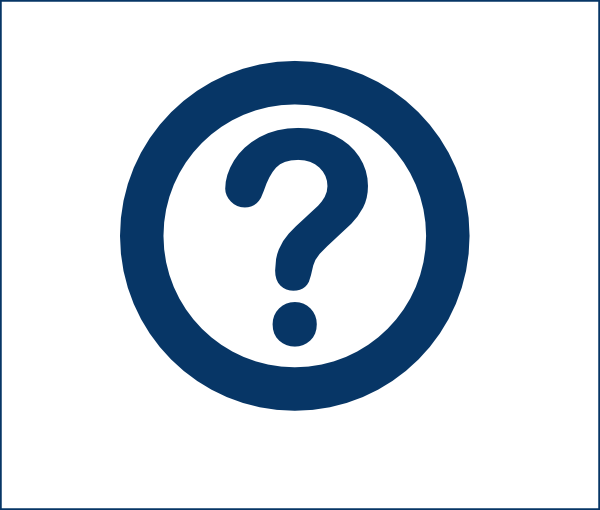 unknown** | | | | | | | | |
| --- | --- | --- | --- | --- | --- | --- | --- | --- |
| **First author (Year) [ref]** | **Randomization** | **Control** | **Selection bias: random sequence generation** | **Selection bias:**  **allocation concealment** | **Performance bias** | **Detection bias** | **Attrition bias** | **Reporting bias** |
| Alicandro (1995) [29] | **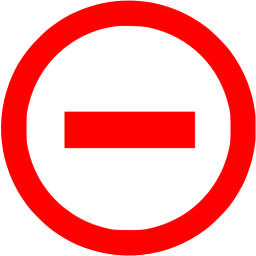** | **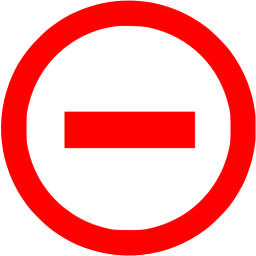** | **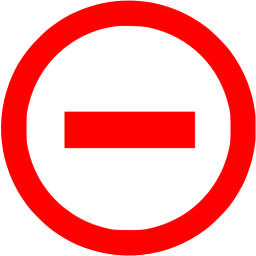** | **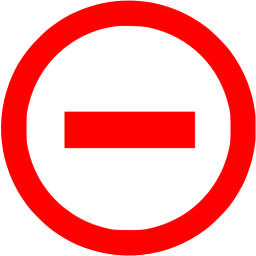** | **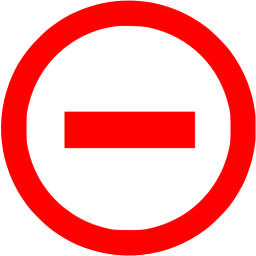** | **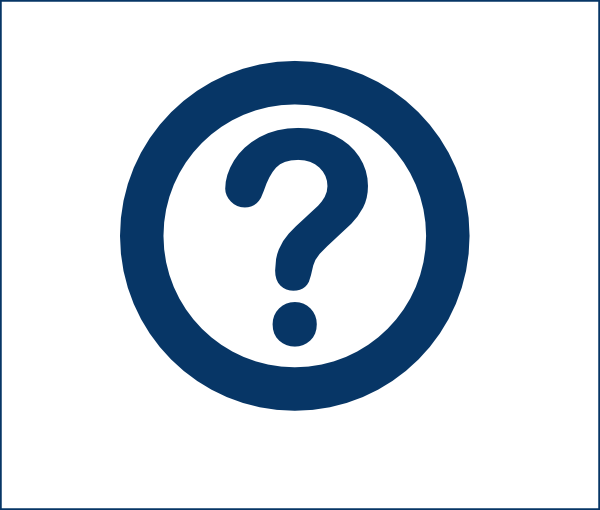** | **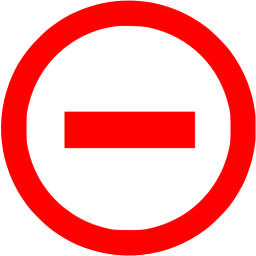** | **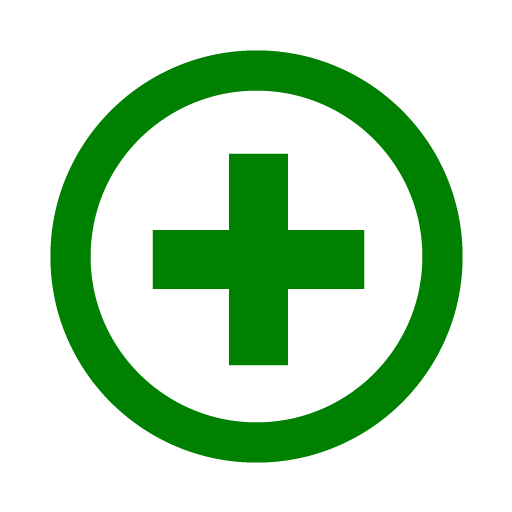** |
| Key (2003) [30] | **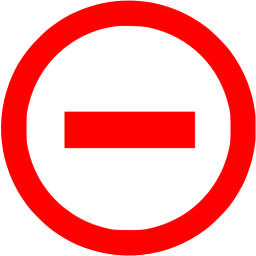** | **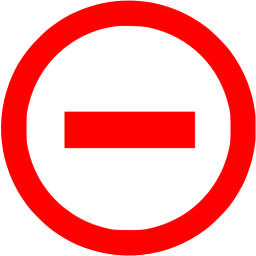** | **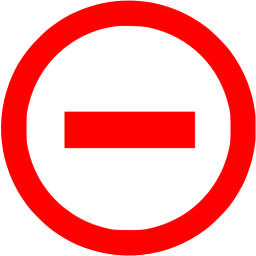** | **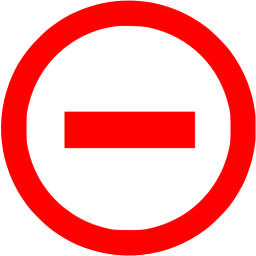** | **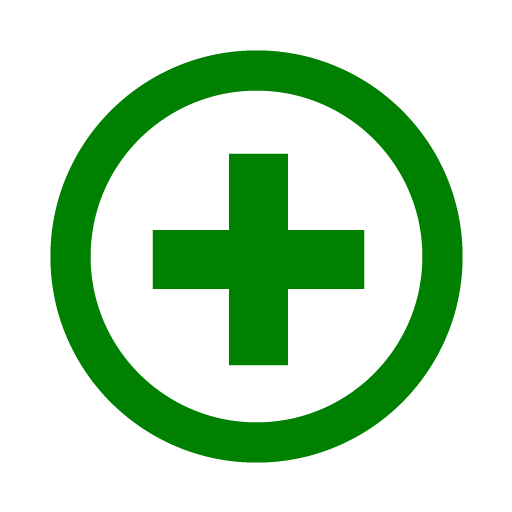** | **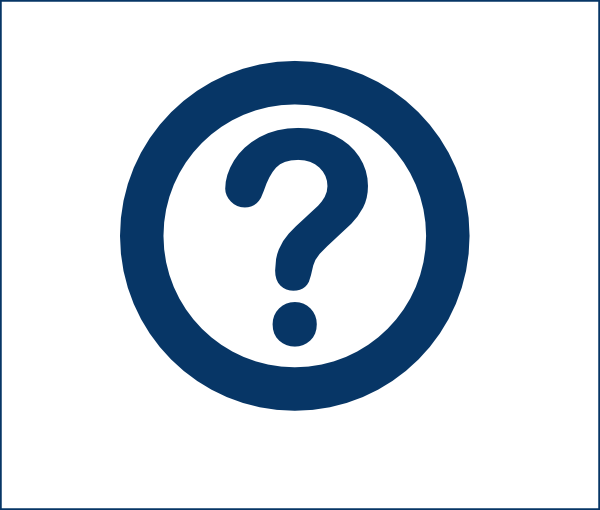** | **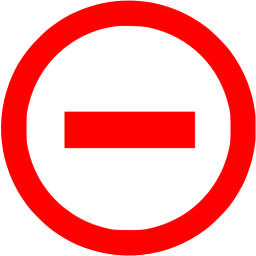** | **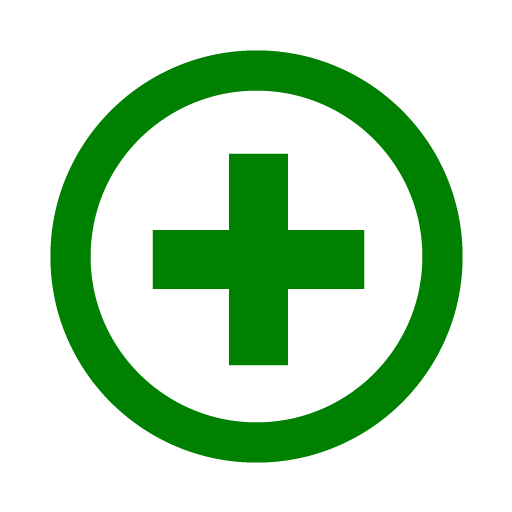** |
| Snooks (2004a) [28] | **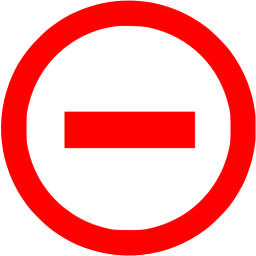** | **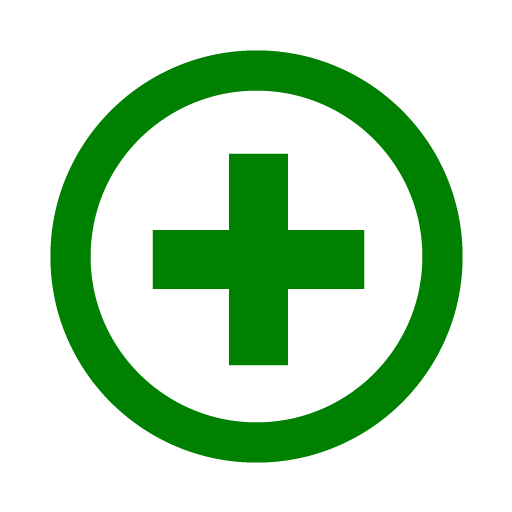** | **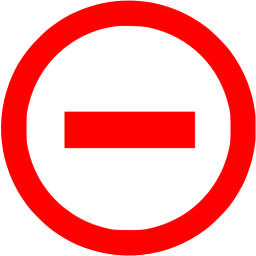** | **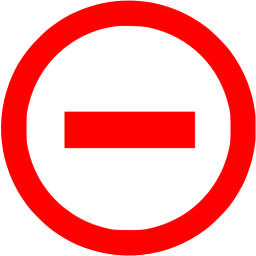** | **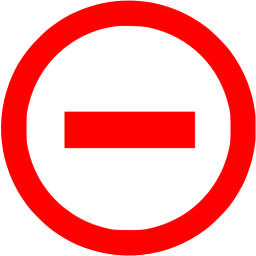** | **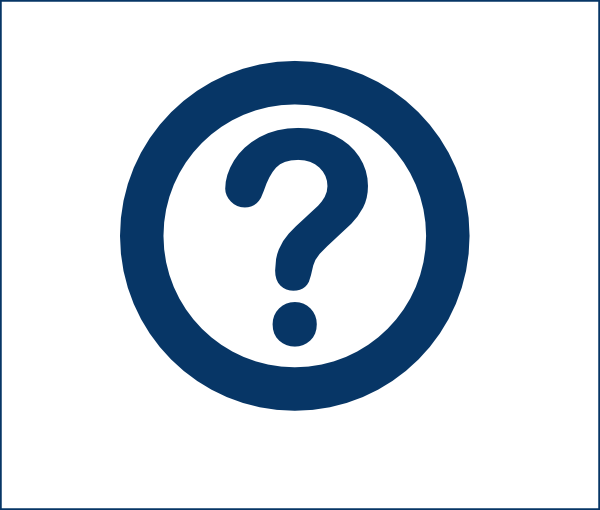** | **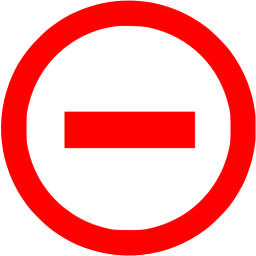** | **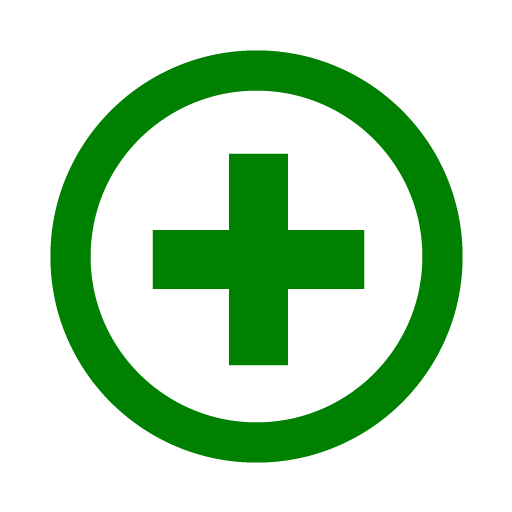** |
| Snooks (2014) [27] | **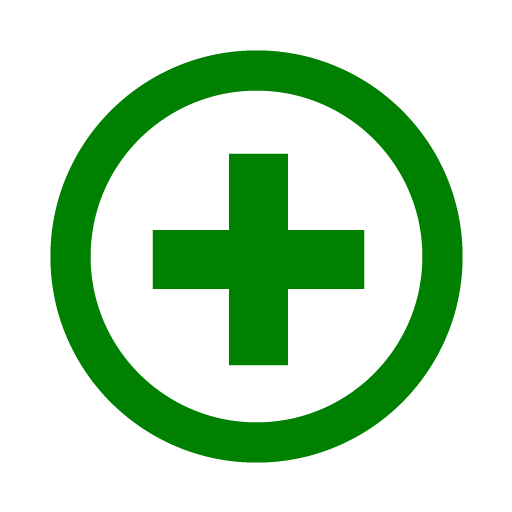** | **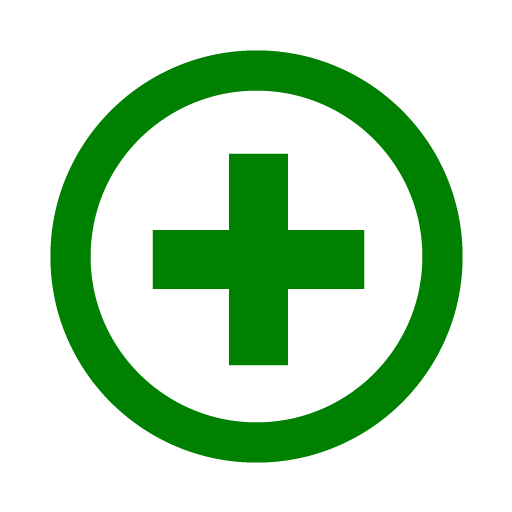** | **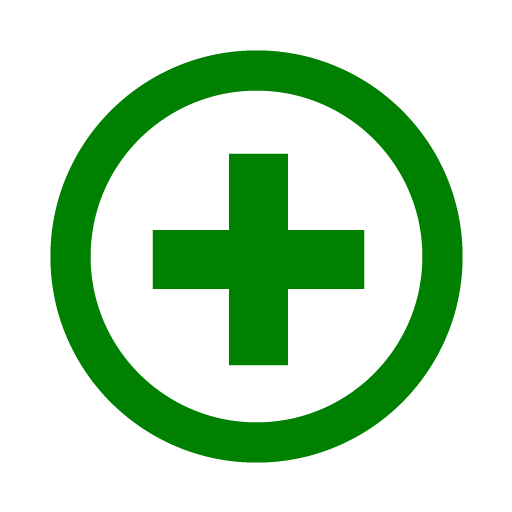** | **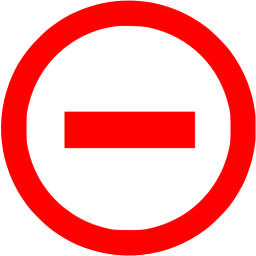** | **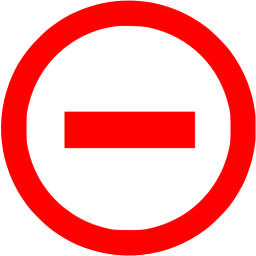** | **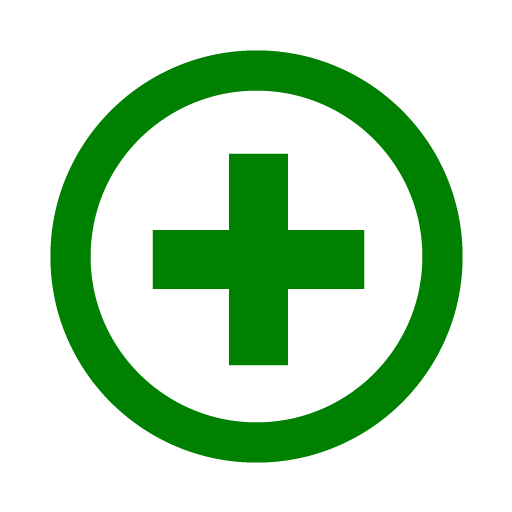** | **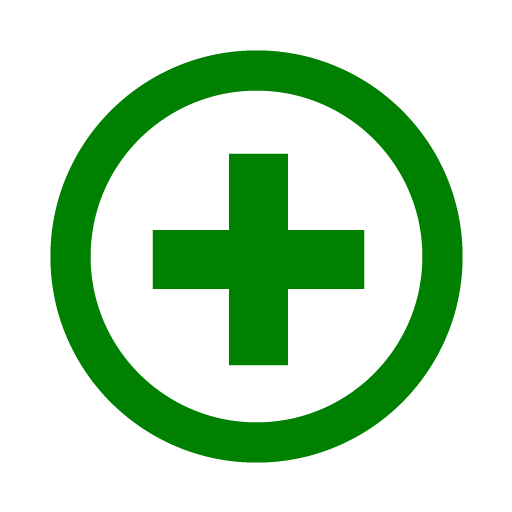** | **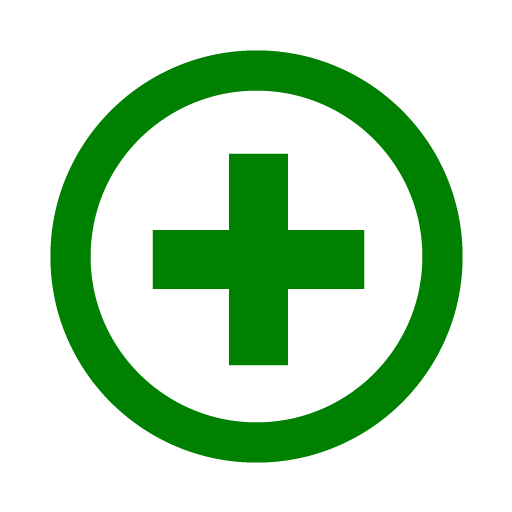** |
