# Supplementary material for: A patient-safety and professional perspective on non-conveyance in ambulance care: a systematic review
Source: Scand J Trauma Resusc Emerg Med. 2017 Jul 17;25:71. doi: 10.1186/s13049-017-0409-6 (PMC5513207; doi:10.1186/s13049-017-0409-6)
Supplement: Supplementary file 6 — Appendix 5 Quality of quantitative studies (n = 53) (DOC 5545 kb) [file 13049_2017_409_MOESM6_ESM.doc]

| **Appendix 5 - Quality of quantitative studies (n=53)**  **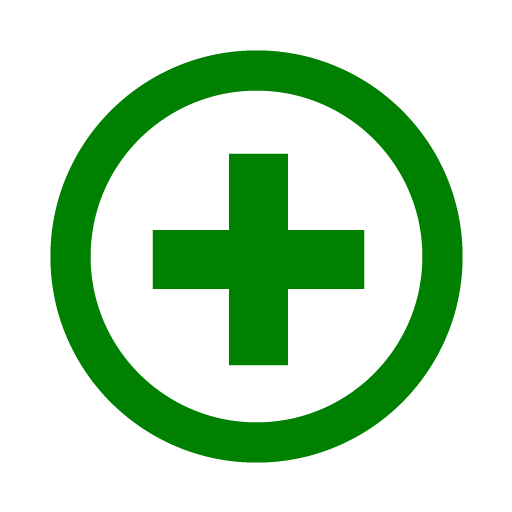 Yes,** 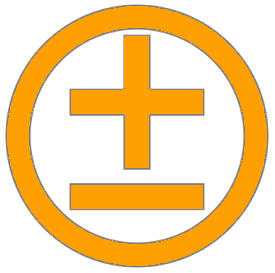**partial , 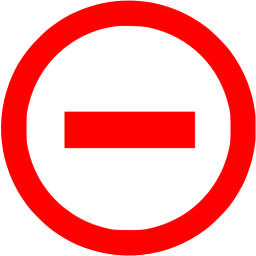 No N/A = Not Available** | | | | | | | | | | | |
| --- | --- | --- | --- | --- | --- | --- | --- | --- | --- | --- | --- |
| **First author (Year) [ref]** | **Question / objective sufficiently described?** | **Study design evident and appropriate?** | **Method of subject/comparison group selection or source of information/input variables described and appropriate?** | **Subject (and comparison group, if applicable) characteristics sufficiently described?** | **Outcome and (if applicable) exposure measure(s) well defined and robust to measurement / misclassification bias? means of assessment reported?** | **Sample size appropriate?** | **Analytic methods described/justified and appropriate?** | **Some estimate of variance is reported for the main results?** | **Controlled for confounding?** | **Results reported in sufficient detail?** | **Conclusions supported by the results?** |
| Alrazeeni (2016) [31] | **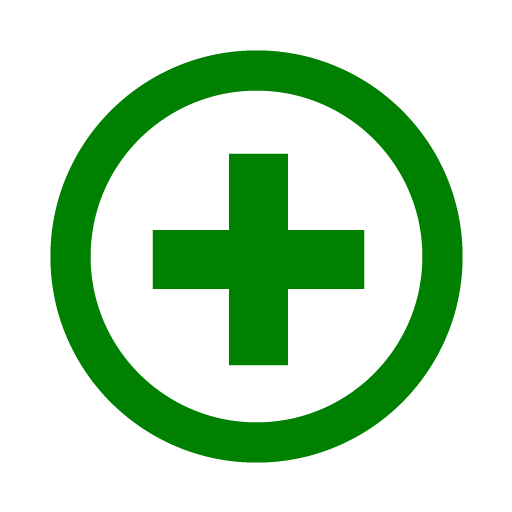** | **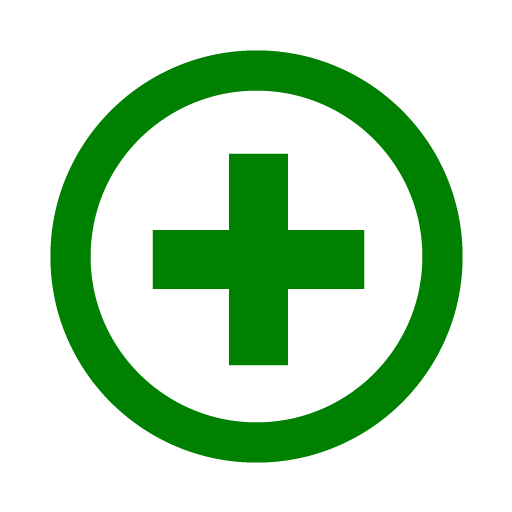** | **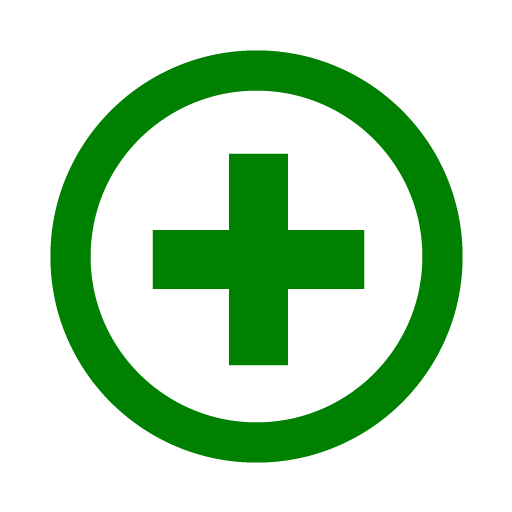** | **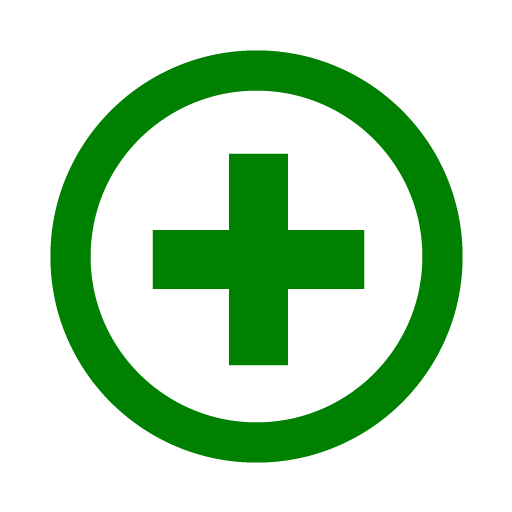** | **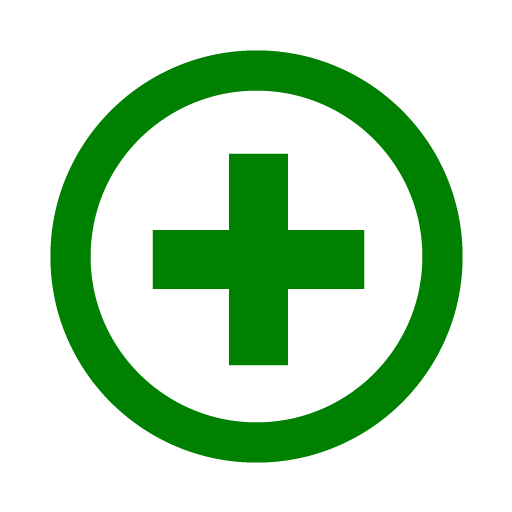** | **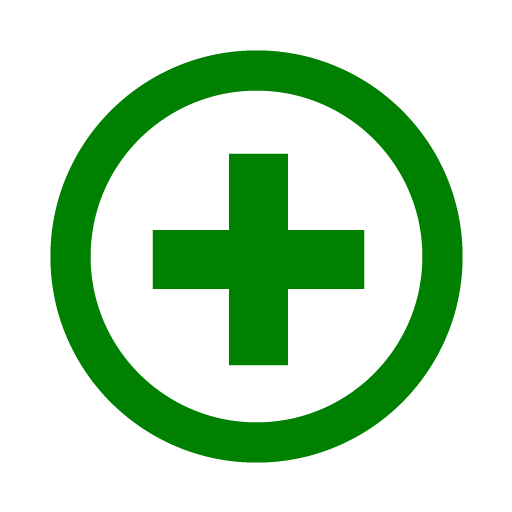** | **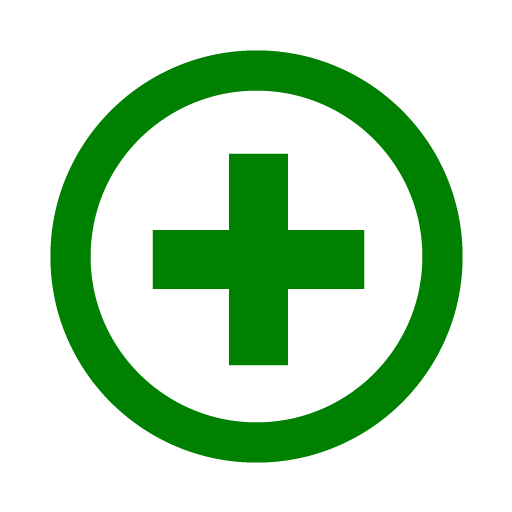** | N/A | N/A | **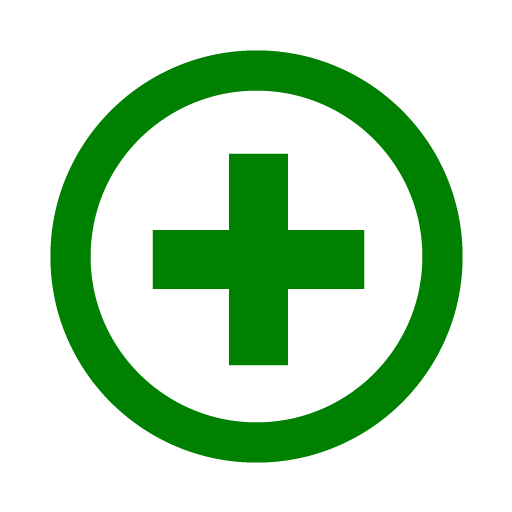** | **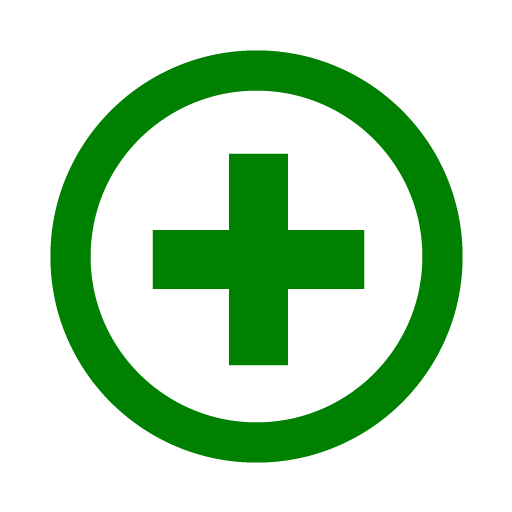** |
| Anderson (2002) Denmark [32] | **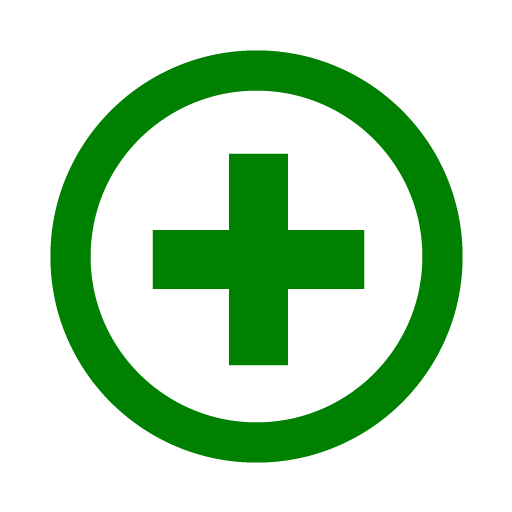** | **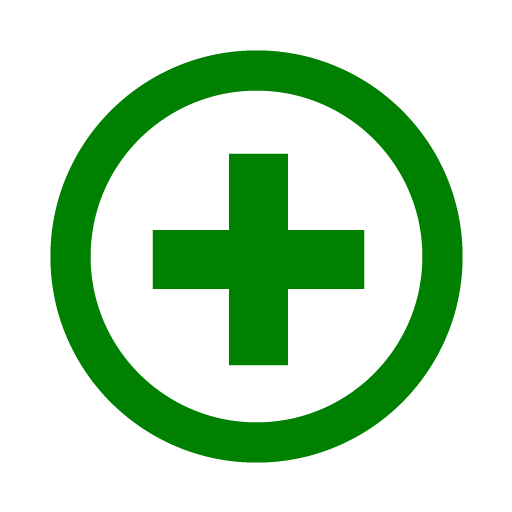** | **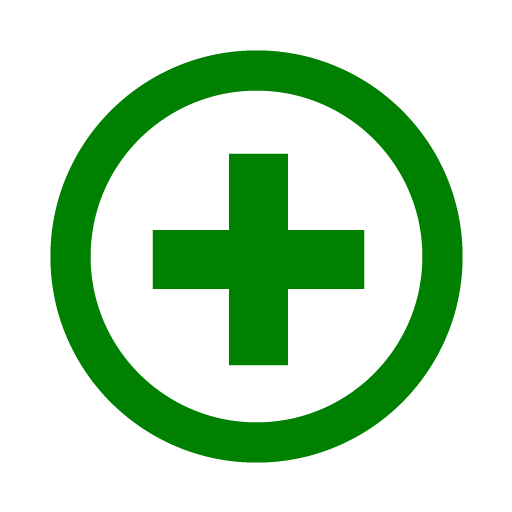** | **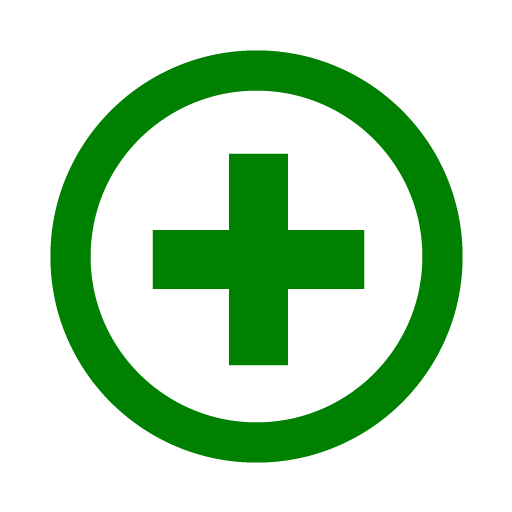** | **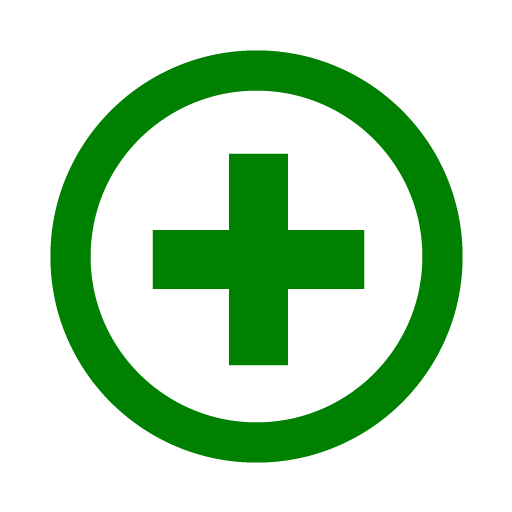** | **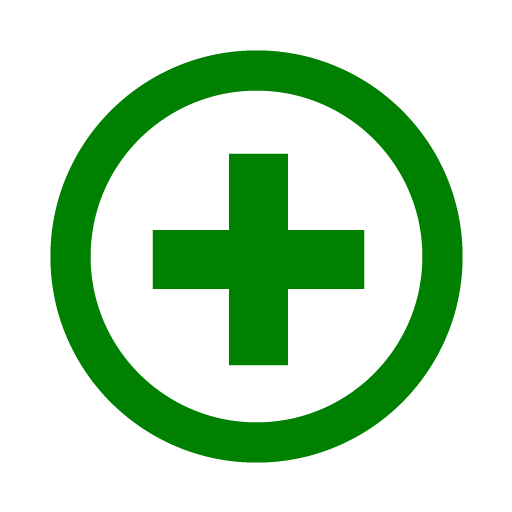** | **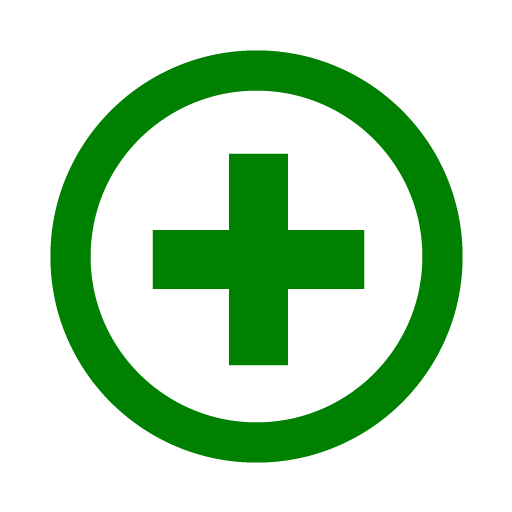** | **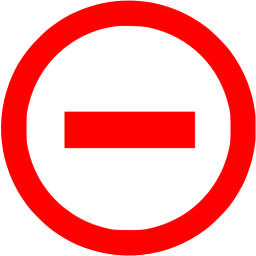** | N/A | **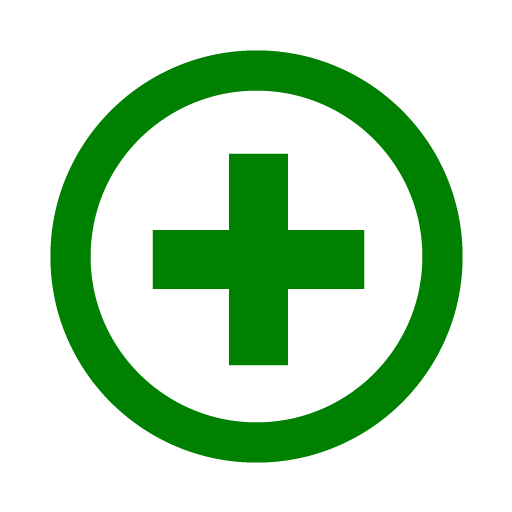** | **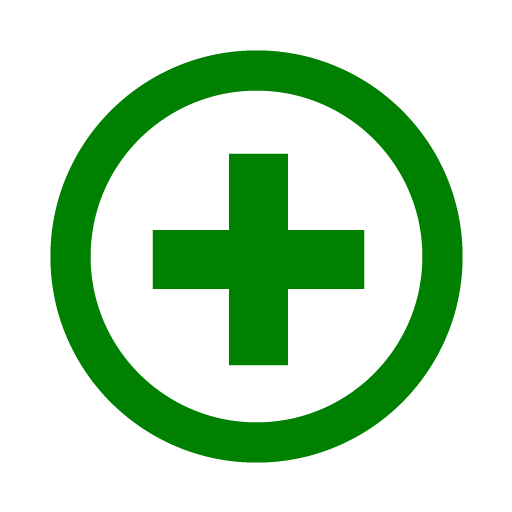** |
| Burstein (1996) USA [56] | **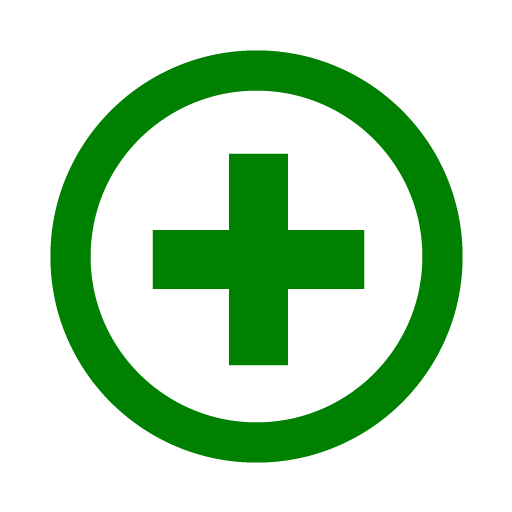** | **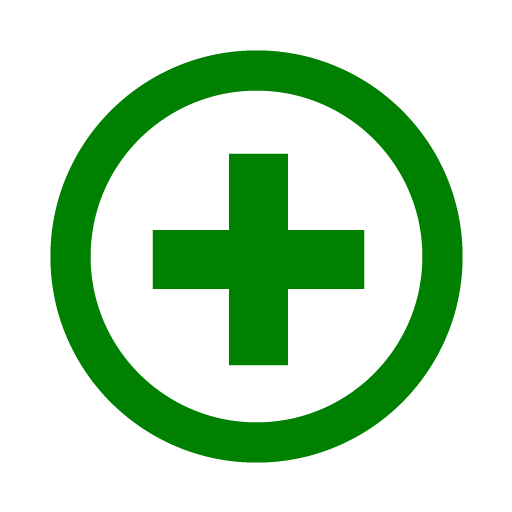** | **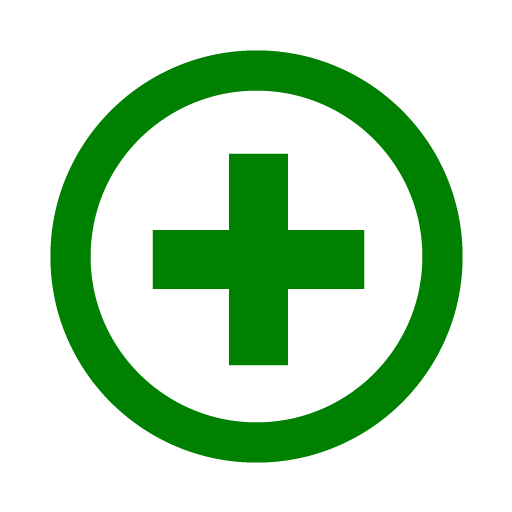** | **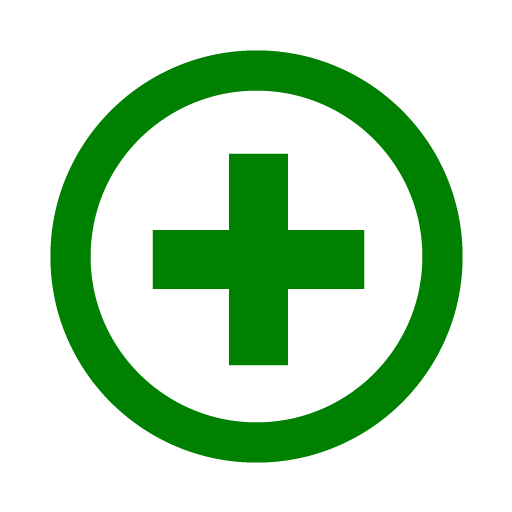** | **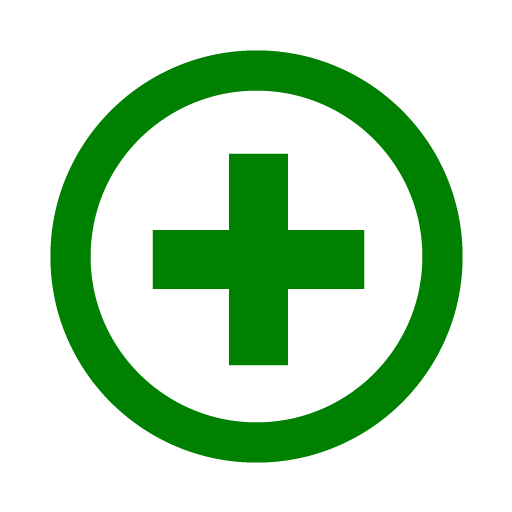** | **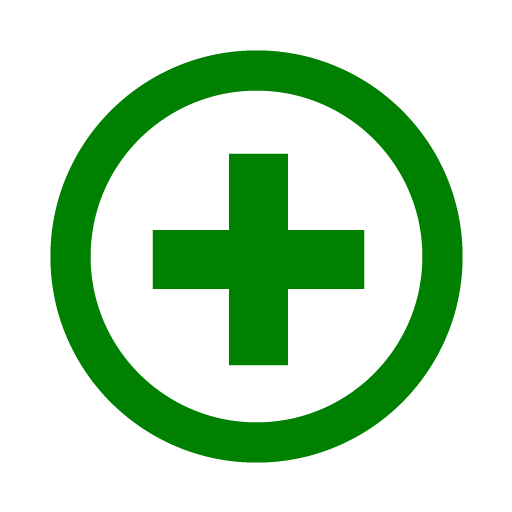** | **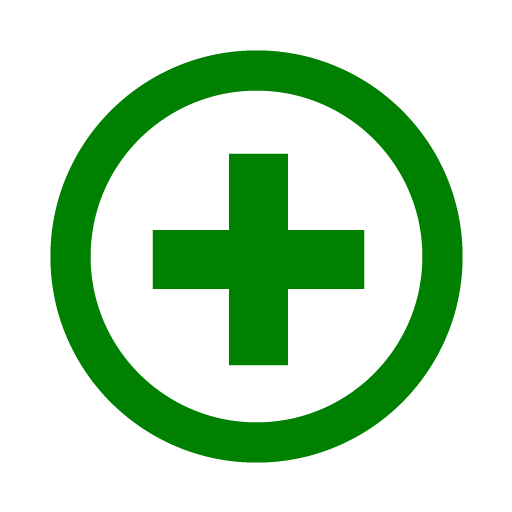** | N/A | N/A | **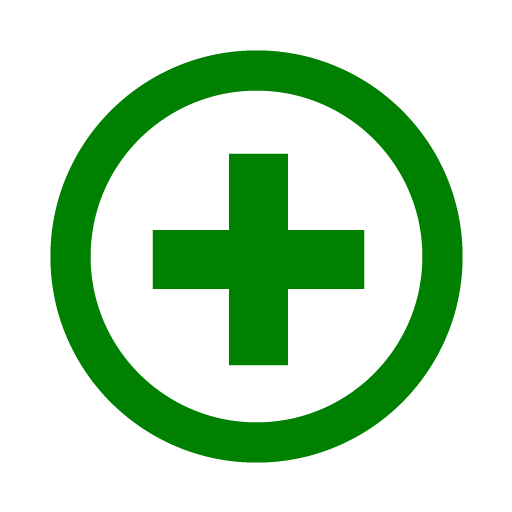** | **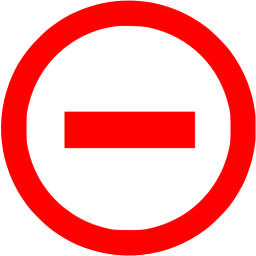** |
| Burstein (1998) USA [57] | **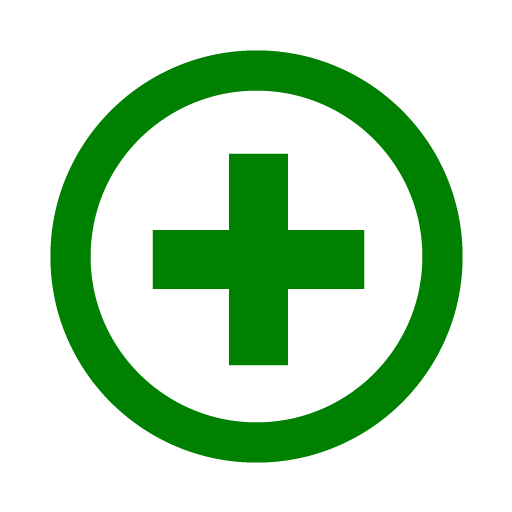** | **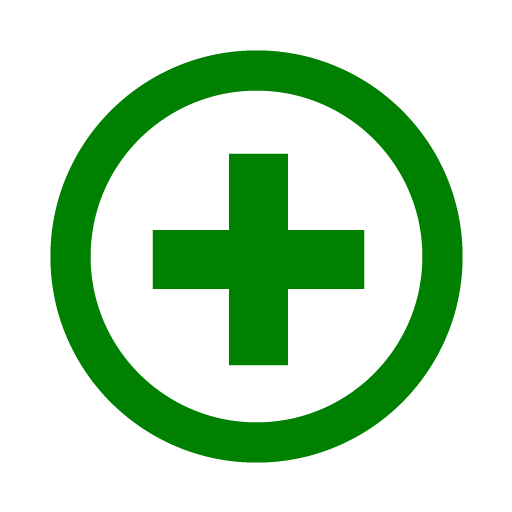** | **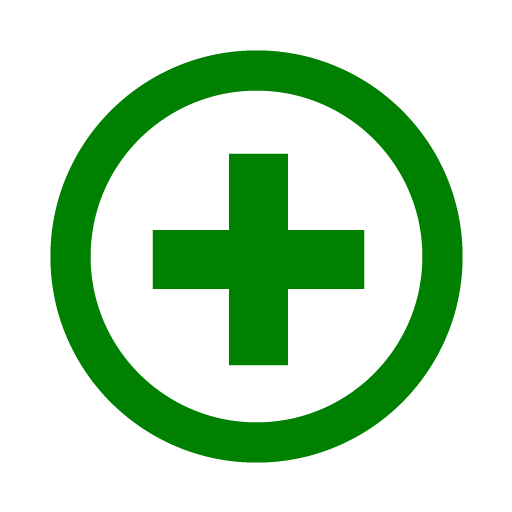** | **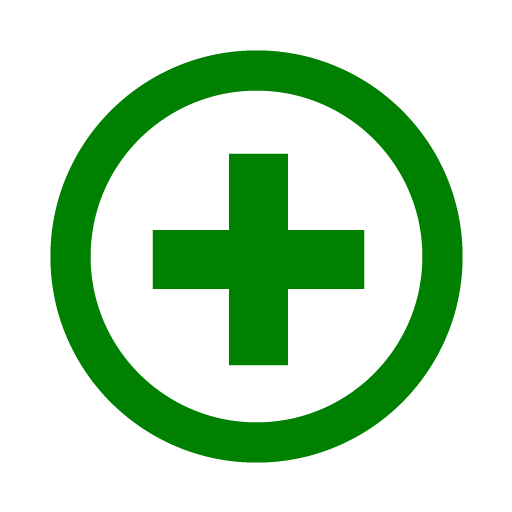** | 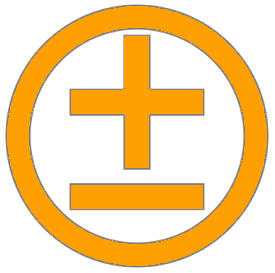 | **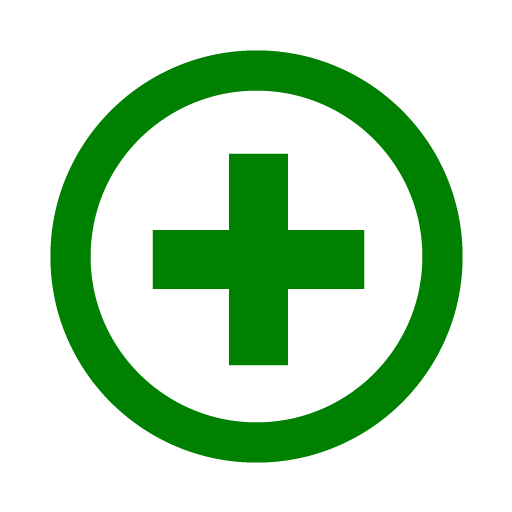** | **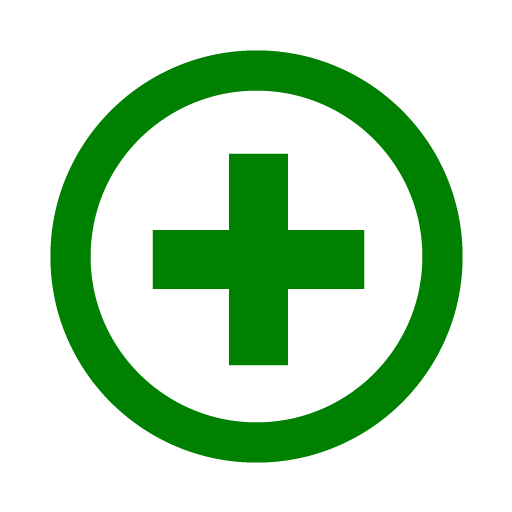** | **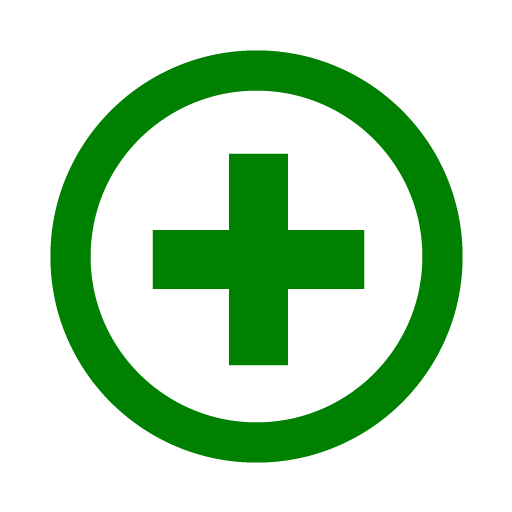** | N/A | **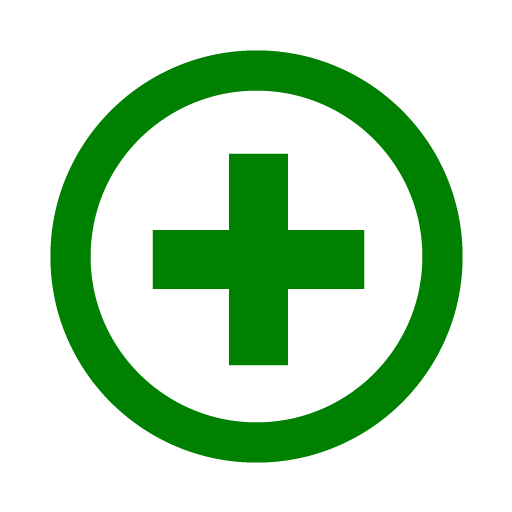** | **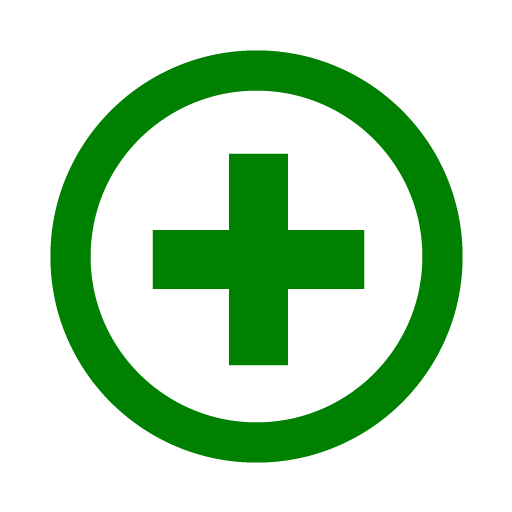** |
| Cain (2003) USA [58] | **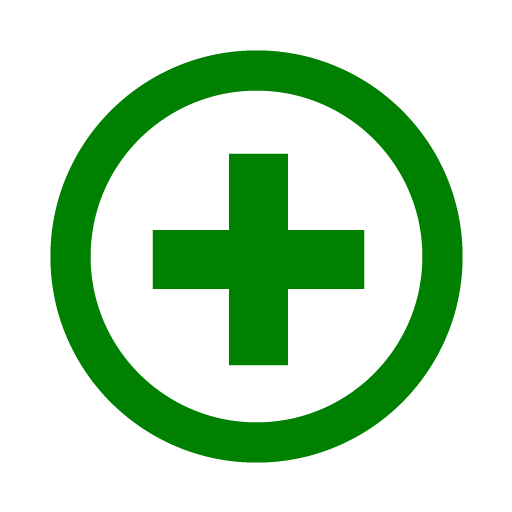** | **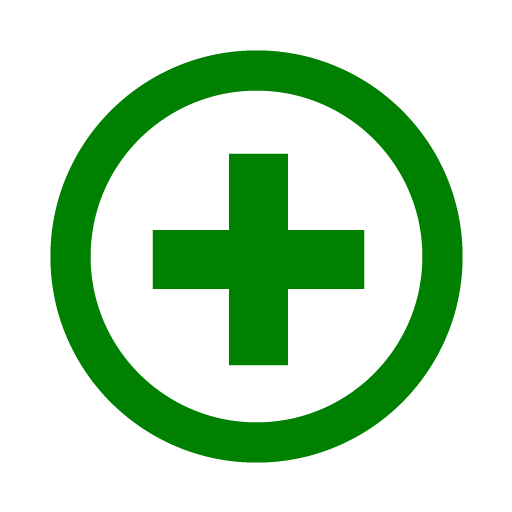** | **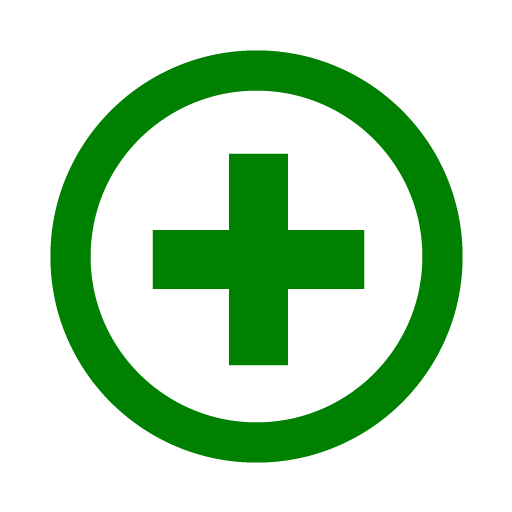** | **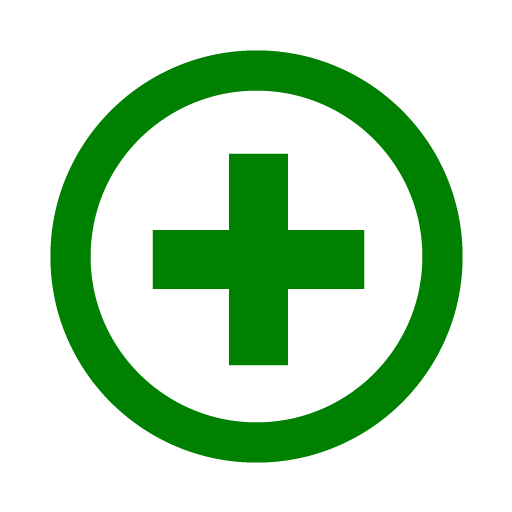** | **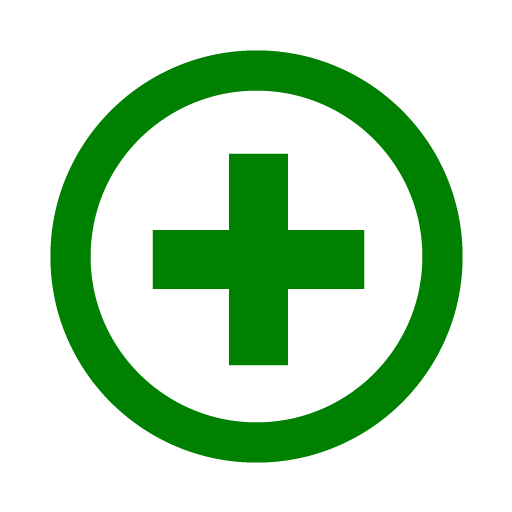** | N/A | **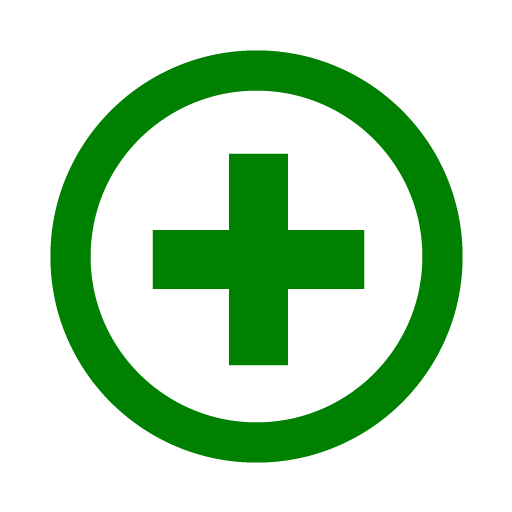** | 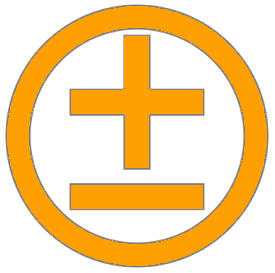 | N/A | **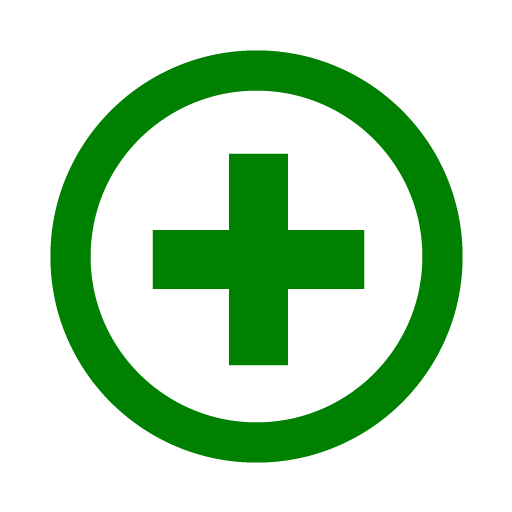** | **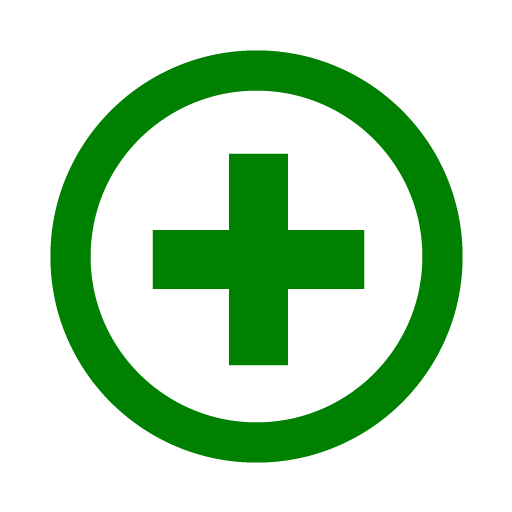** |
| Carter (2012) Canada [59] | **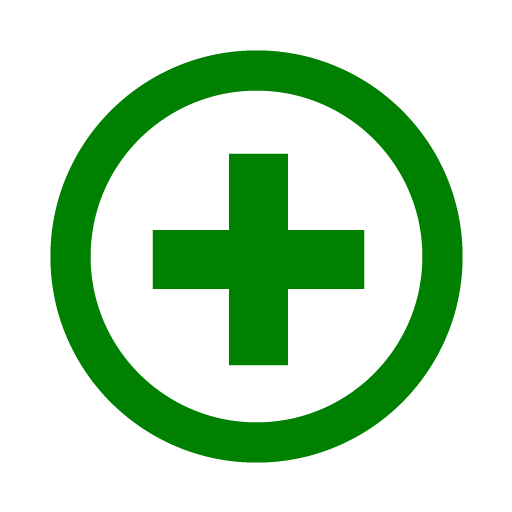** | **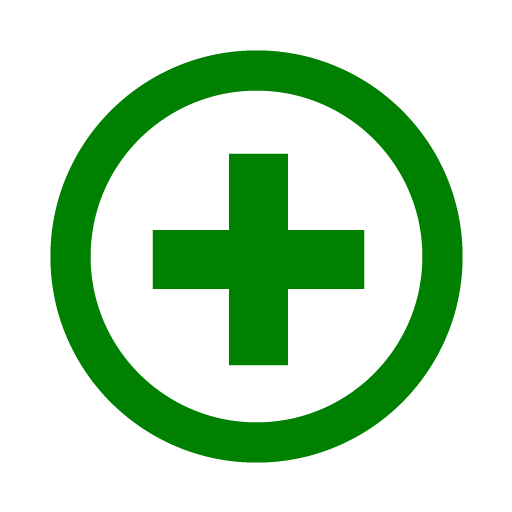** | **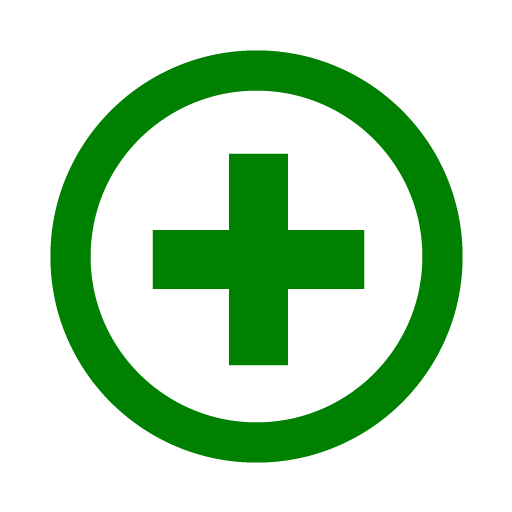** | **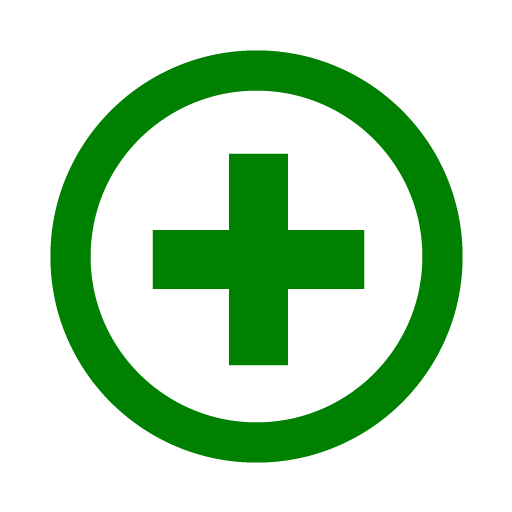** | **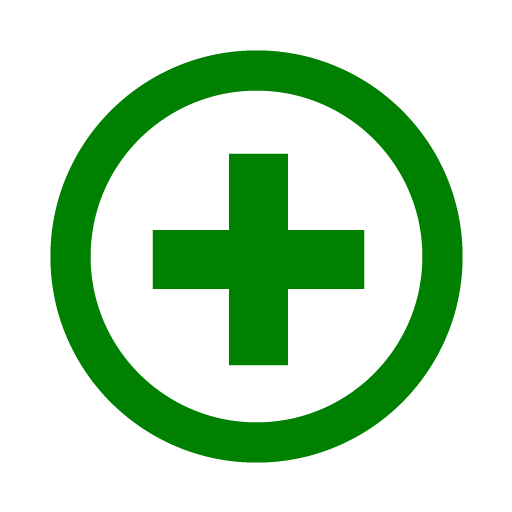** | **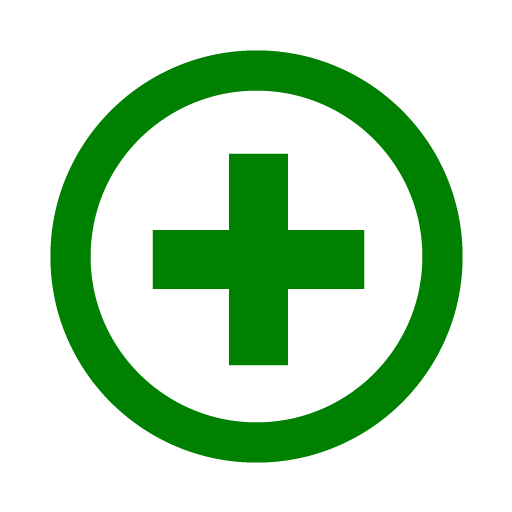** | **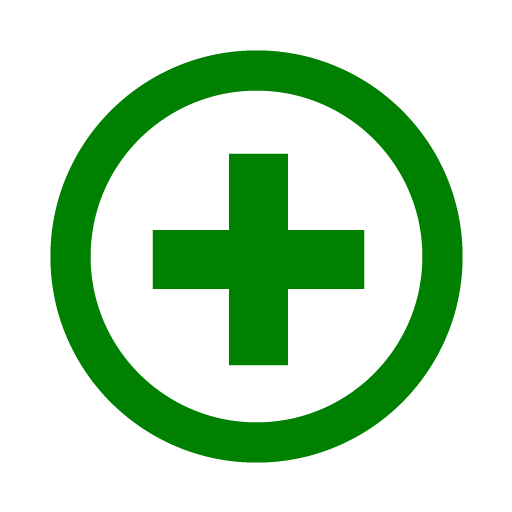** | **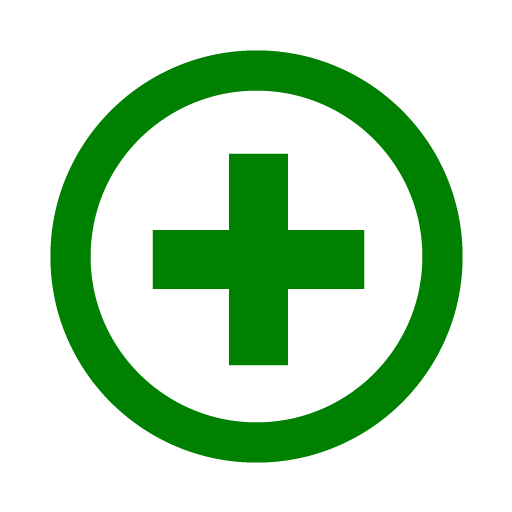** | N/A | 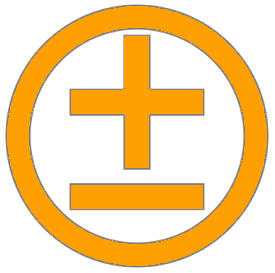 | 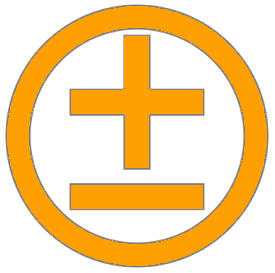 |
| Chen (1996) Taiwan [60] | **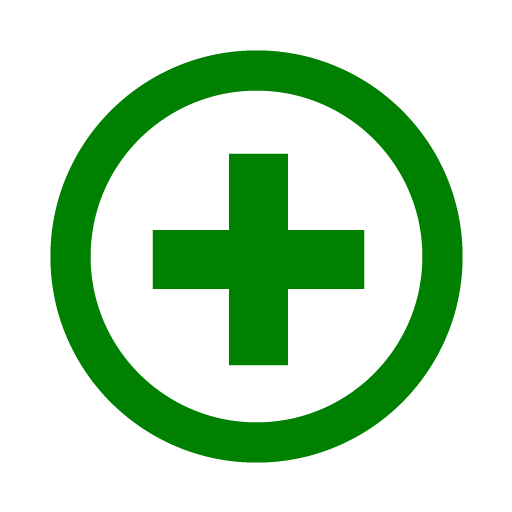** | **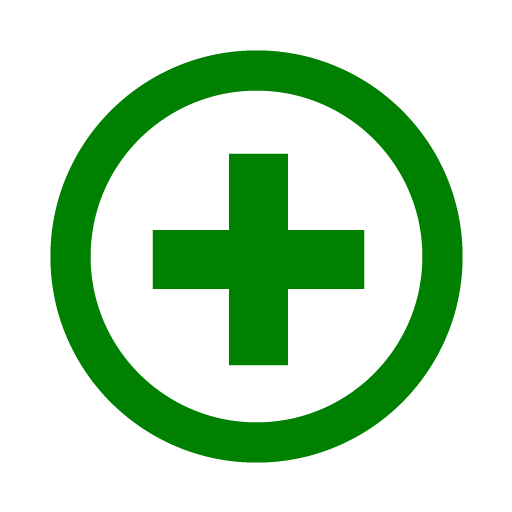** | **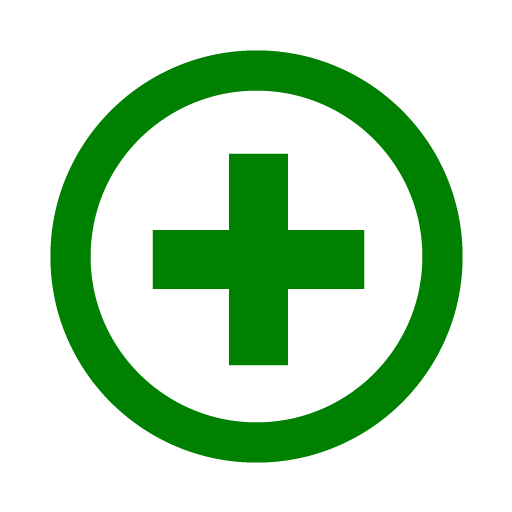** | 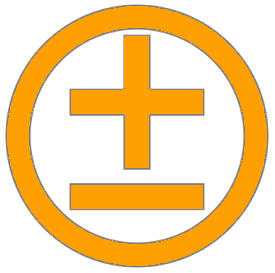 | 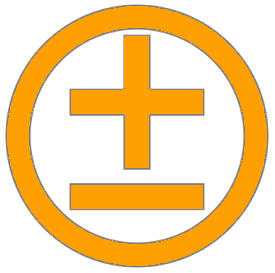 | **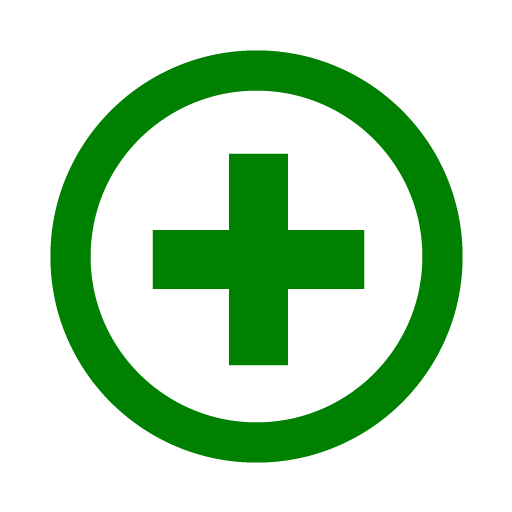** | **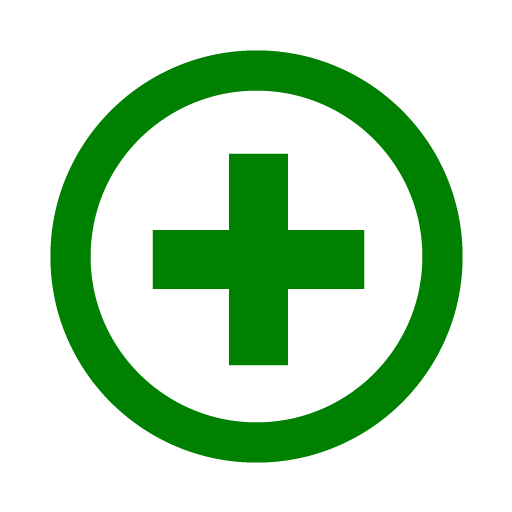** | N/A | N/A | **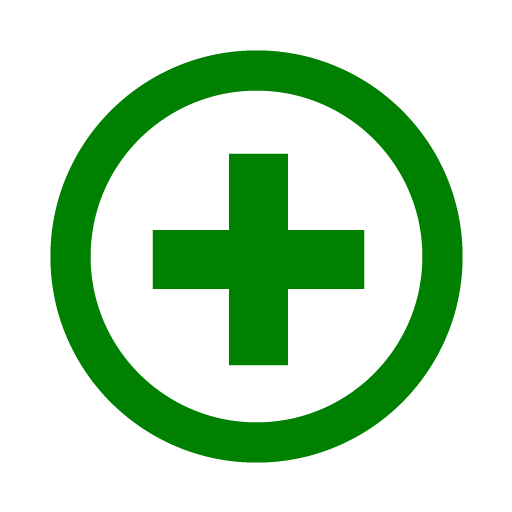** | 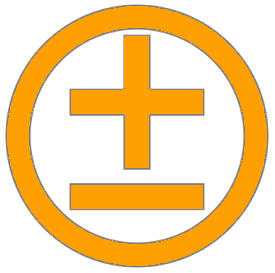 |
| Cone (1995) USA [8] | **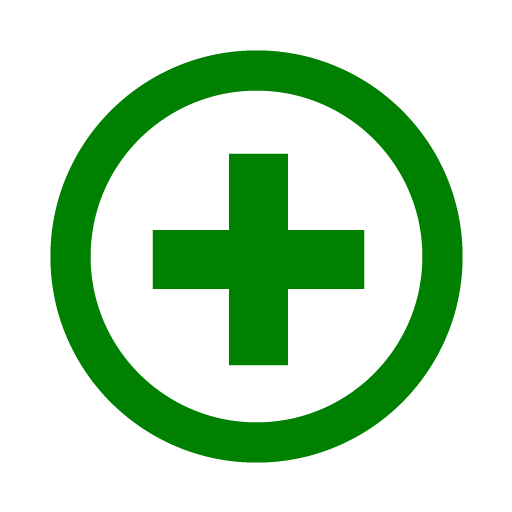** | **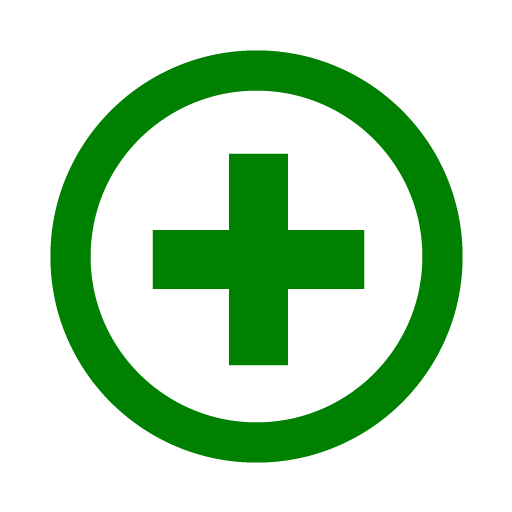** | **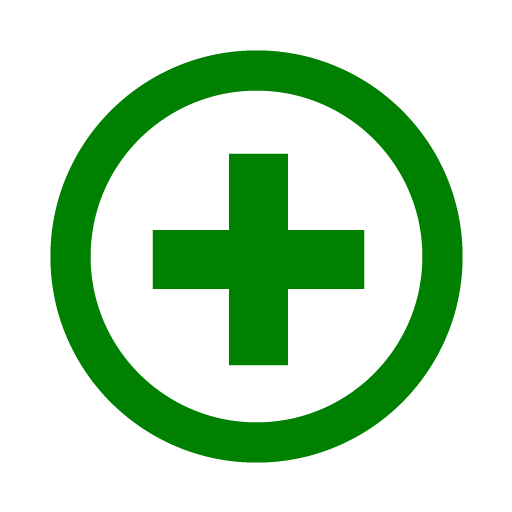** | 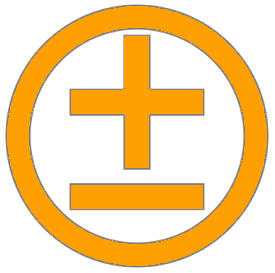 | **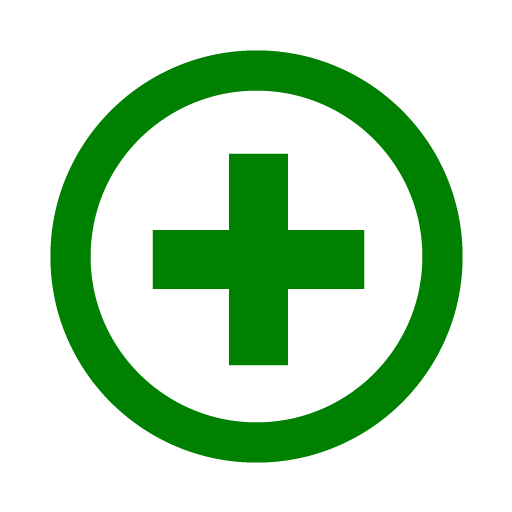** | N/A | **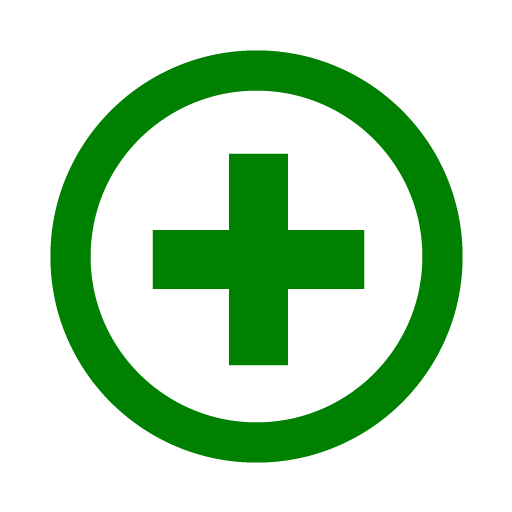** | N/A | N/A | **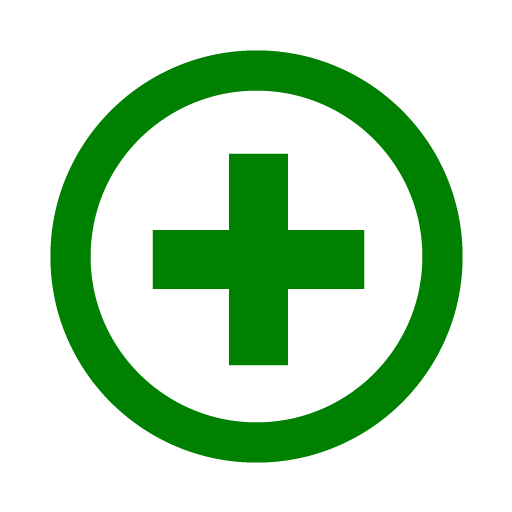** | **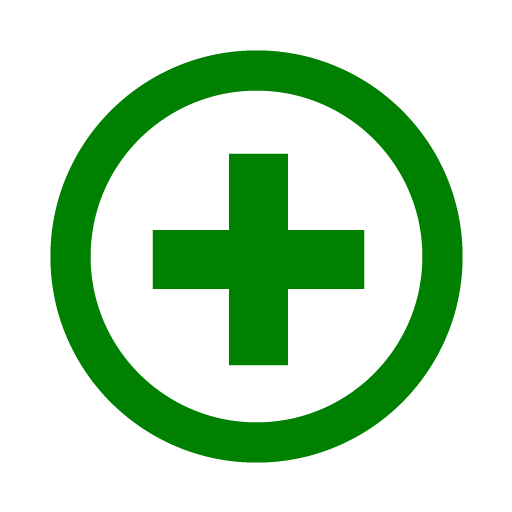** |
| Deasy (2008) Ireland [61] | 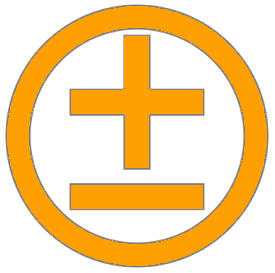 | **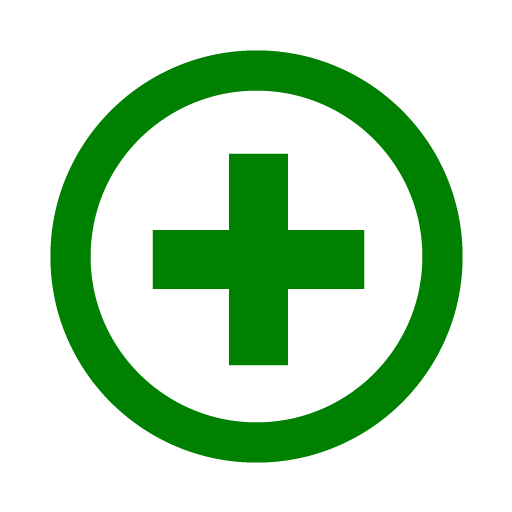** | 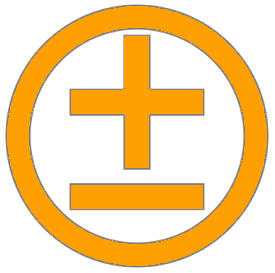 | 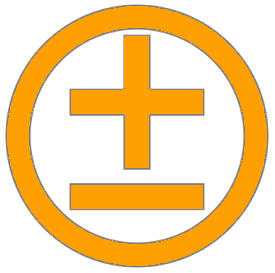 | **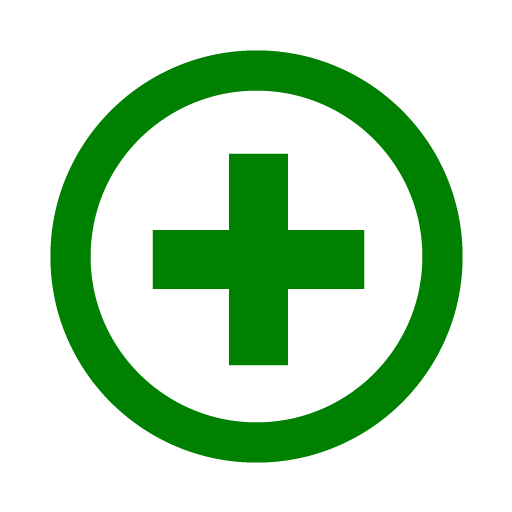** | **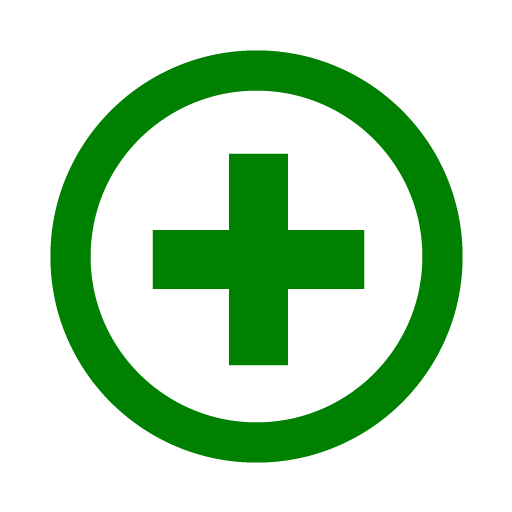** | **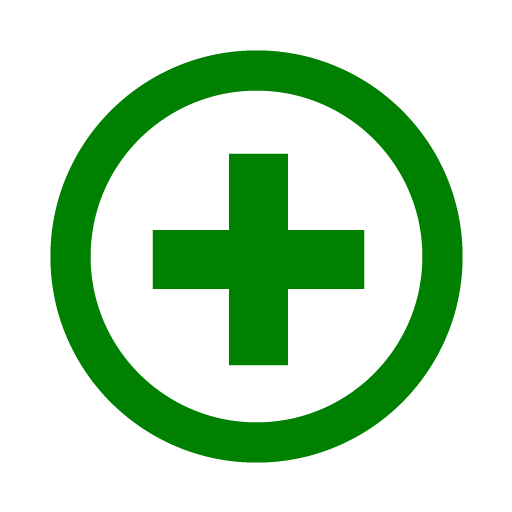** | N/A | N/A | **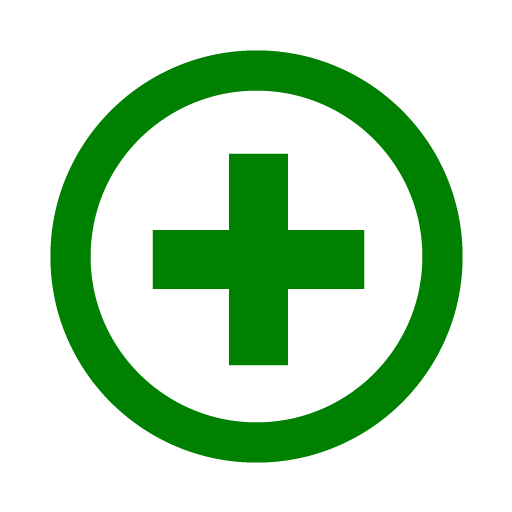** | **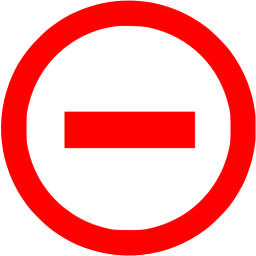** |
| Gerlacher (2001) USA [79] | **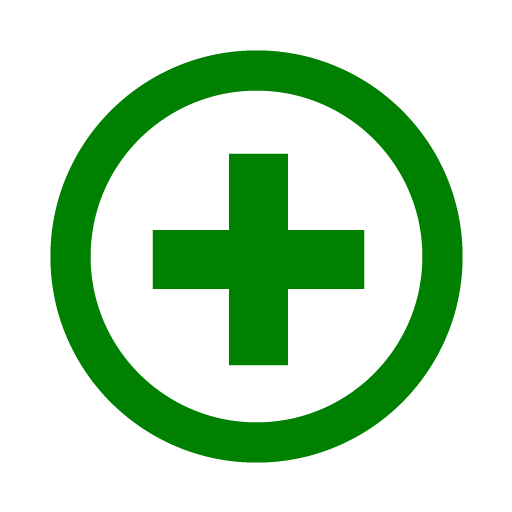** | **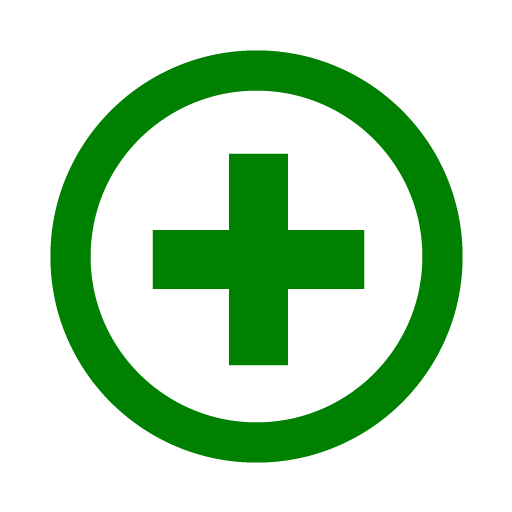** | **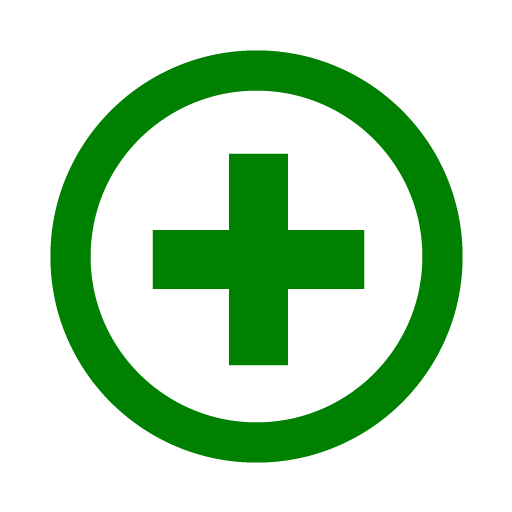** | **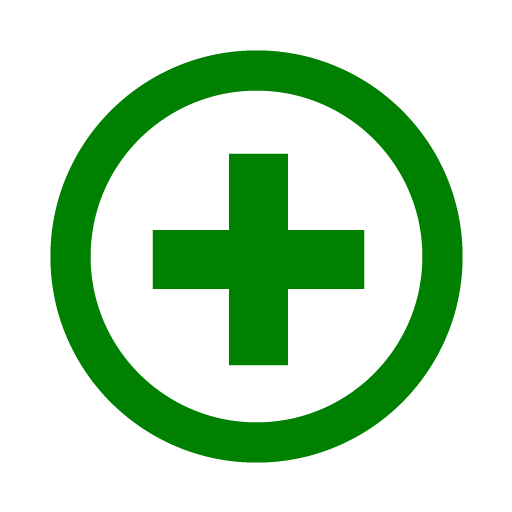** | **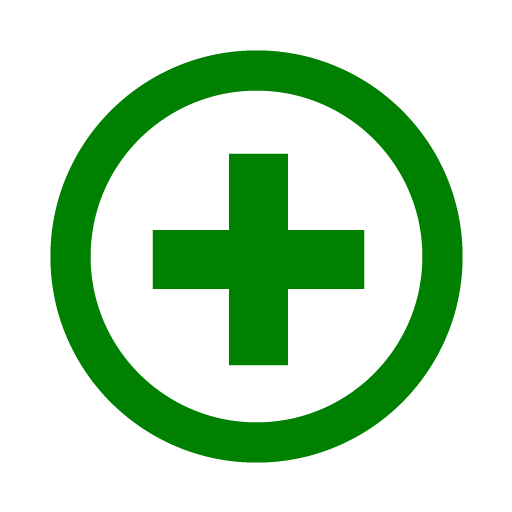** | **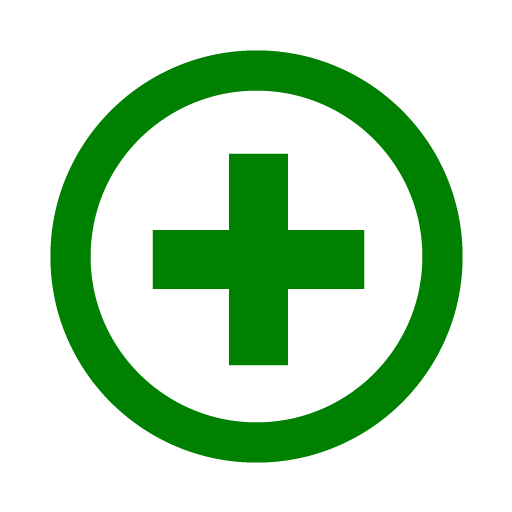** | **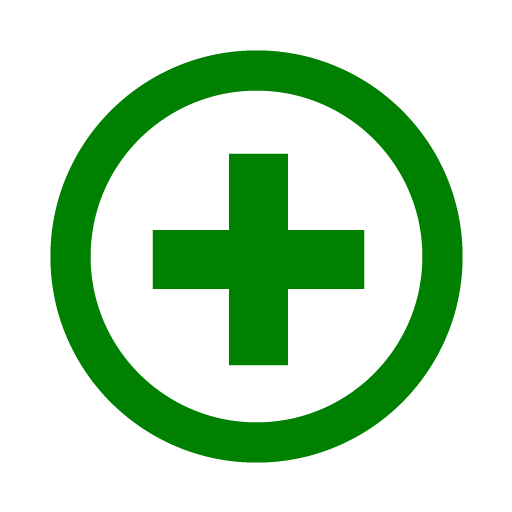** | **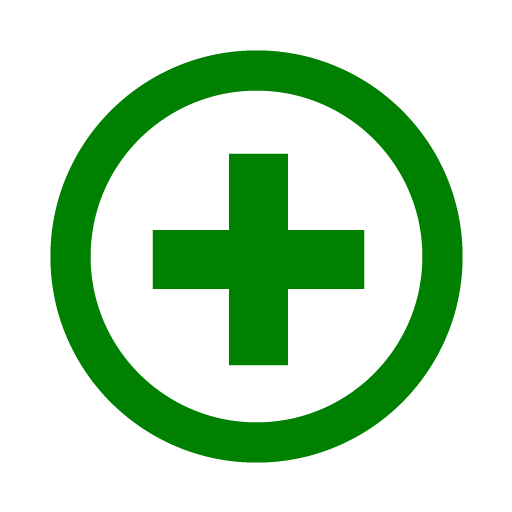** | N/A | **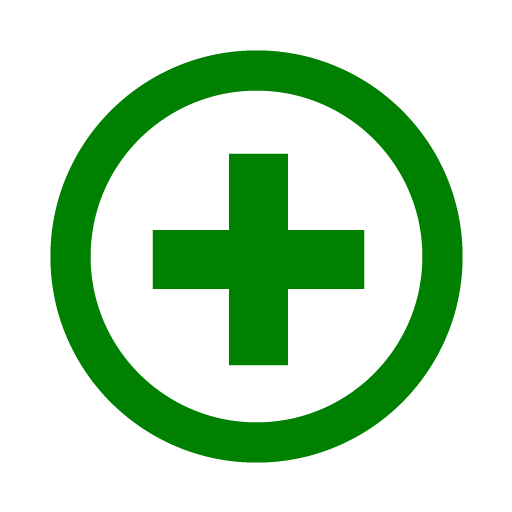** | **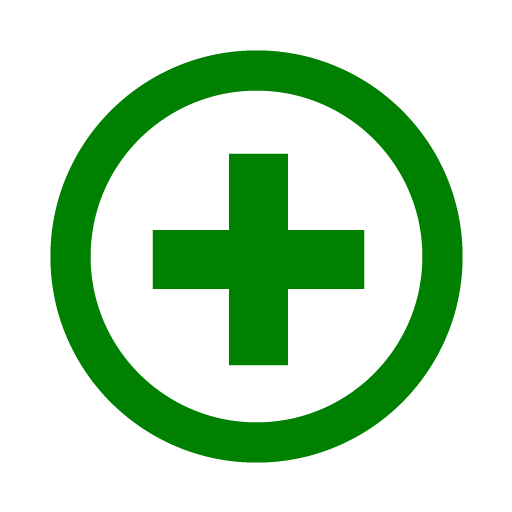** |
| Goldstein (2015) Canada [33] | **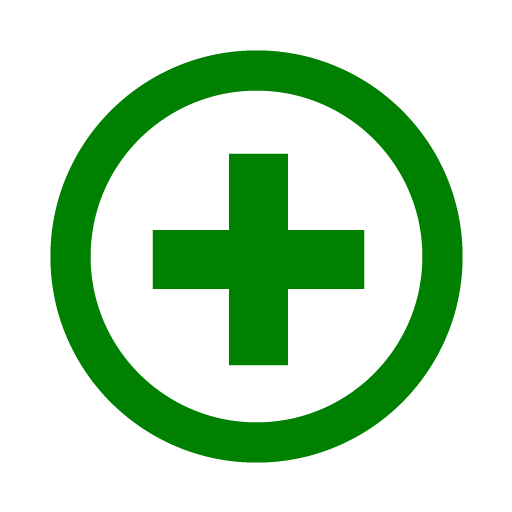** | **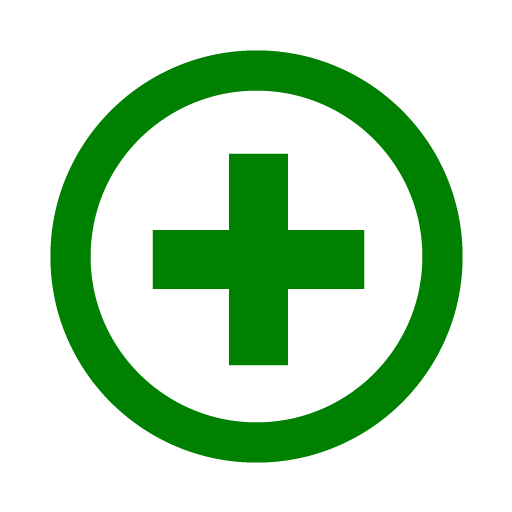** | **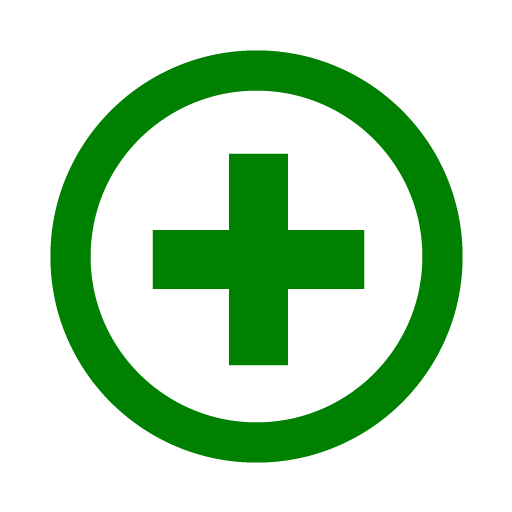** | **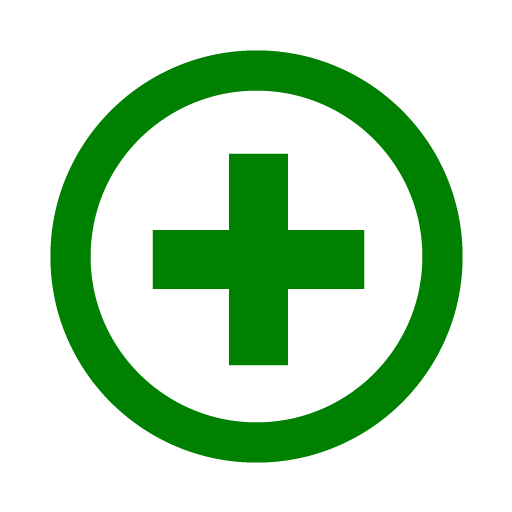** |  |  |  |  | N/A |  |  |
| Haines (2006) USA [62] |  |  |  |  |  |  |  |  | N/A |  |  |
| Hipskind (1997) USA [63] |  |  |  |  |  |  |  |  | N/A |  |  |
| Højfeld (2014) Denmark [34] |  |  |  |  |  |  |  |  | N/A |  |  |
| Jensen (2013) Canada [64] |  |  |  |  |  |  |  |  | N/A |  |  |
| Kahalé (2006) Canada [65] |  |  |  |  |  |  |  |  | N/A |  |  |
| Kamper (2001) USA [35] |  |  |  |  |  |  |  |  | N/A |  |  |
| Kannikeswaran (2007) USA [36] |  |  |  |  |  |  |  |  | N/A |  |  |
| Knight (2003) USA [37] |  |  |  |  |  |  |  |  | N/A |  |  |
| Lerner (2003) USA [66] |  |  |  |  |  | N/A |  | N/A | N/A |  |  |
| Magnusson (2016) Sweden [38] |  |  |  |  |  |  |  |  | N/A |  |  |
| Marks (2002) UK [9] |  |  |  |  |  |  |  |  | N/A |  |  |
| Mechem (1998) USA [67] |  |  |  |  |  |  |  |  | N/A |  |  |
| Minhas (2015) Canada [39] |  |  |  |  |  |  |  |  | N/A |  |  |
| Moss (1998) USA [40] |  |  |  |  |  |  |  | N/A | N/A |  |  |
| Newton (2015) South Africa [68] |  |  |  |  |  |  |  | N/A | N/A |  |  |
| Persse (2002) USA [69] |  |  |  |  |  |  |  |  | N/A |  |  |
| Peyravi (2013) Iran [41] |  |  |  |  |  |  |  | N/A | N/A |  |  |
| Peyravi (2015) Sweden [42] |  |  |  |  |  |  |  |  | N/A |  |  |
| Pringle (2005) USA [43] |  |  |  |  |  |  |  |  | N/A |  |  |
| Rudolph (2011) Denmark [44] |  |  |  |  |  |  |  |  | N/A |  |  |
| Schmidt (1998) USA [71] |  |  |  |  |  |  |  |  | N/A |  |  |
| Schmidt (2000) USA [72] |  |  |  |  |  |  |  |  |  |  |  |
| Schmidt (2001) USA [70] |  |  |  |  |  |  |  |  | N/A |  |  |
| Schmidt (2006) USA [45] |  |  |  |  |  |  |  |  | N/A |  |  |
| Selden (1990) USA [46] |  |  |  |  |  |  |  |  | N/A |  |  |
| Seltzer (2001) USA [47] |  |  |  |  |  |  | N/A |  | N/A |  |  |
| Shaw (2006) UK [81] |  |  |  |  |  | N/A |  | N/A | N/A |  |  |
| Simpson (2014a) Australia [74] |  |  |  |  |  |  |  |  |  |  |  |
| Simpson (2014b) Australia [73] |  |  |  |  |  |  |  |  | N/A |  |  |
| Socransky (1998) USA [48] |  |  |  |  |  |  |  |  | N/A |  |  |
| Stark (1990) USA [49] |  |  |  |  |  |  |  |  |  |  |  |
| Staudenmayer (2011) USA [50] |  |  |  |  |  |  |  |  | N/A |  |  |
| Strote (2008) USA [75] |  |  |  |  |  |  |  |  |  |  |  |
| Stuhlmiller (2005) USA [51] |  |  |  |  |  |  |  | N/A | N/A |  |  |
| Tiedemann (2013) Australia [76] |  |  |  |  |  |  |  |  | N/A |  |  |
| Tohira (2016a) Australia [53] |  |  |  |  |  |  |  |  |  |  |  |
| Tohira (2016b) Australia [52] |  |  |  |  |  |  |  |  |  |  |  |
| Van der Pols (2011) Netherlands [77] |  |  |  |  |  |  |  |  | N/A |  |  |
| Vilke (1999) USA [54] |  |  |  |  |  |  |  |  | N/A |  |  |
| Vilke (2002) USA [78] |  |  |  |  |  |  |  | N/A | N/A |  |  |
| Zachariah (1992) USA [55] |  |  |  |  |  |  |  |  | N/A |  |  |
| Zorab (2015) UK [80] |  |  |  |  |  |  |  |  | N/A |  |  |
