# Supplementary material for: A patient-safety and professional perspective on non-conveyance in ambulance care: a systematic review
Source: Scand J Trauma Resusc Emerg Med. 2017 Jul 17;25:71. doi: 10.1186/s13049-017-0409-6 (PMC5513207; doi:10.1186/s13049-017-0409-6)
Supplement: Supplementary file 7 — Appendix 6 Quality of qualitative studies (n = 8) (DOC 910 kb) [file 13049_2017_409_MOESM7_ESM.doc]

| **Appendix 6 - Quality of qualitative studies (n=8)**  **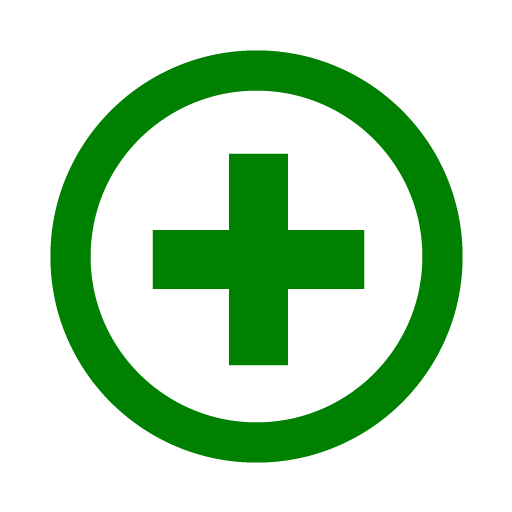 Yes,** 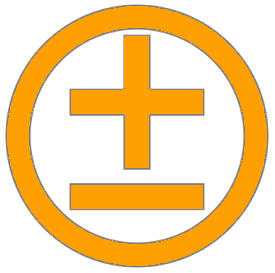**partial , 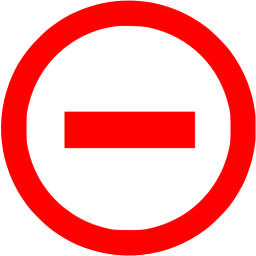 No** | | | | | | | | | | |
| --- | --- | --- | --- | --- | --- | --- | --- | --- | --- | --- |
| **First author (Year) [ref]** | **Question / objective sufficiently described?** | **Study design evident and appropriate?** | **Context for the study clear?** | **Connection to a theoretical framework / wider body of knowledge?** | **Sampling strategy described, relevant and justified?** | **Data collection methods clearly described and systematic?** | **Data analysis clearly described and systematic?** | **Use of verification procedure(s) to establish credibility?** | **Conclusions supported by the results?** | **Reflexivity of the account?** |
| Burrell (2013) UK [82] | **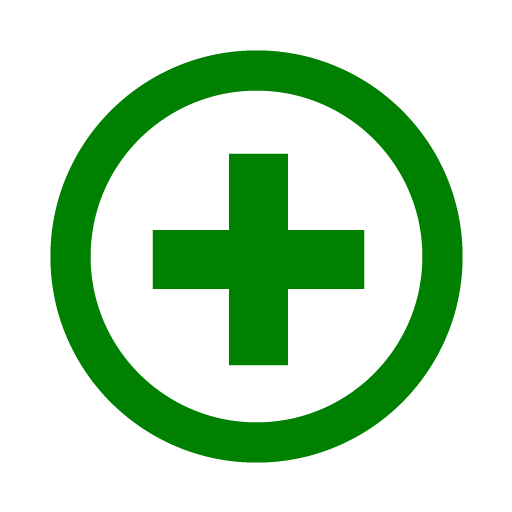** | **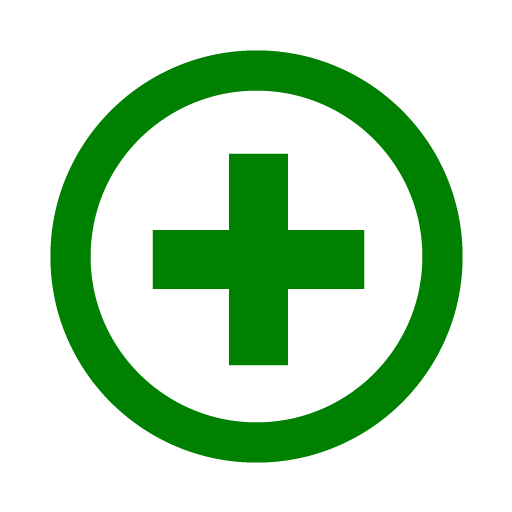** | **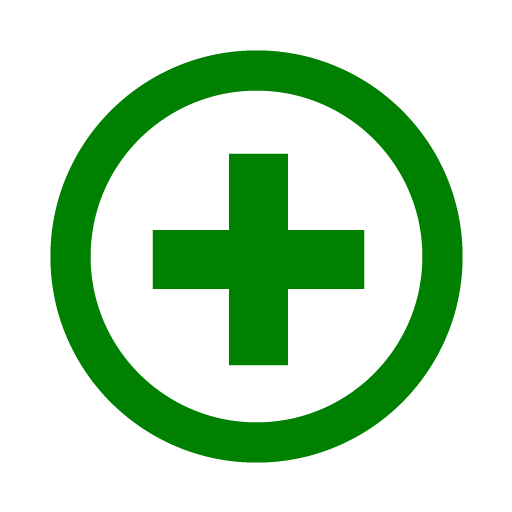** | **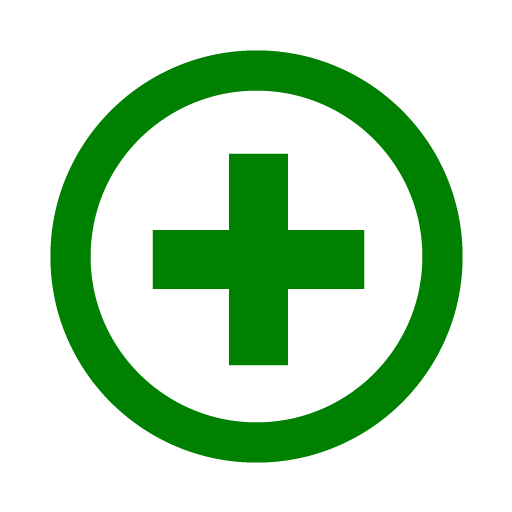** | **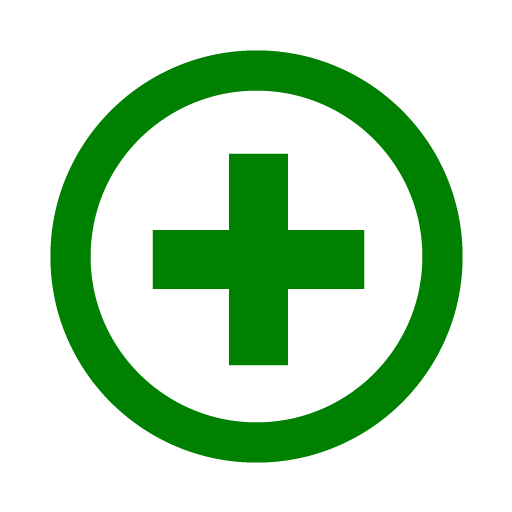** | **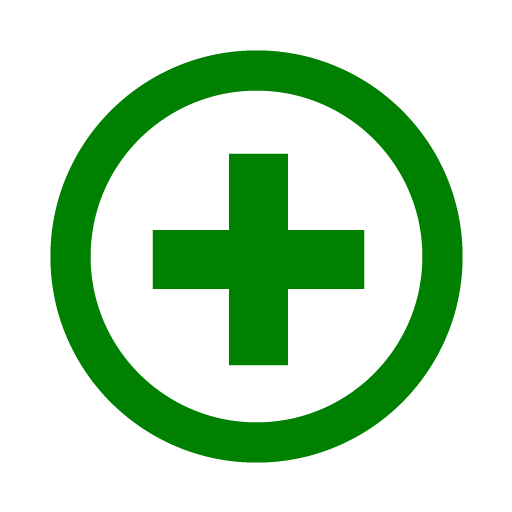** | **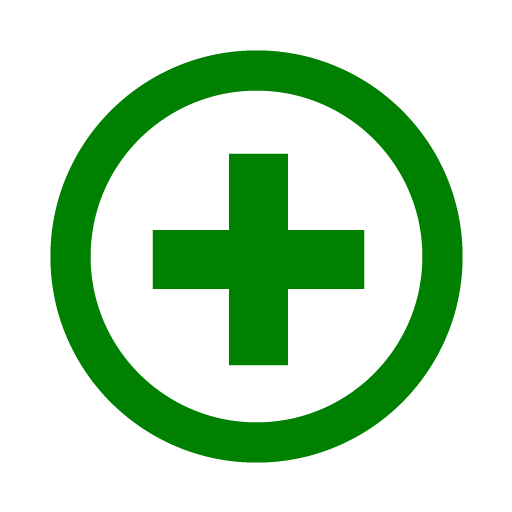** | **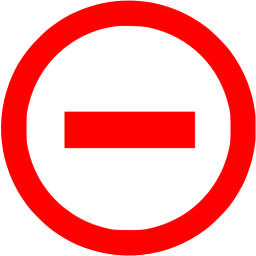** | 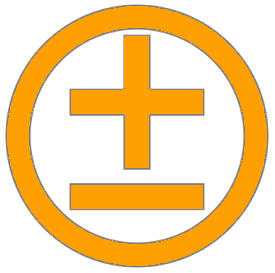 | 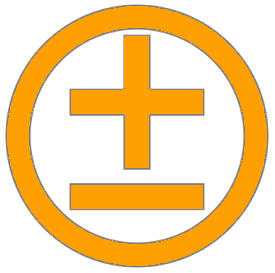 |
| Ebrahimian (2014) Iran [83] | **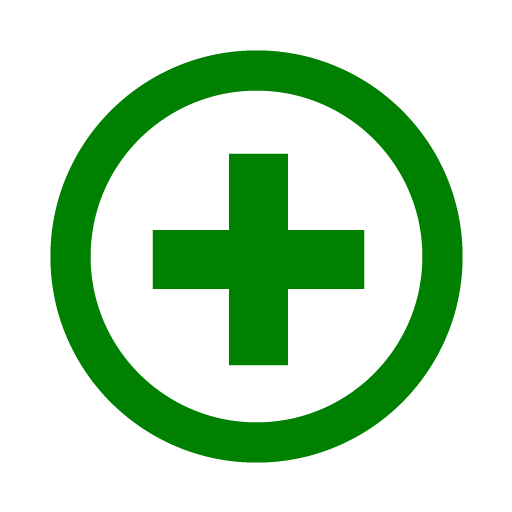** | **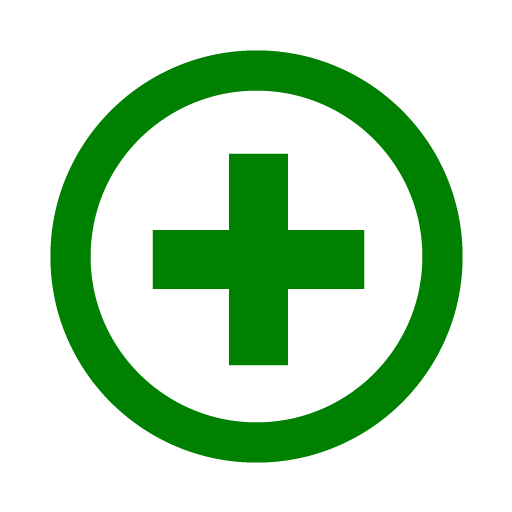** | **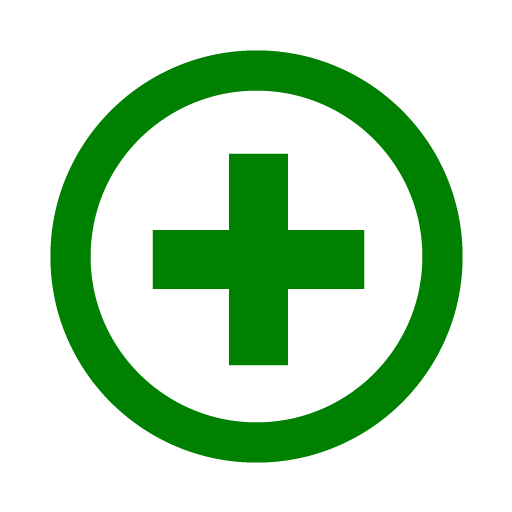** | **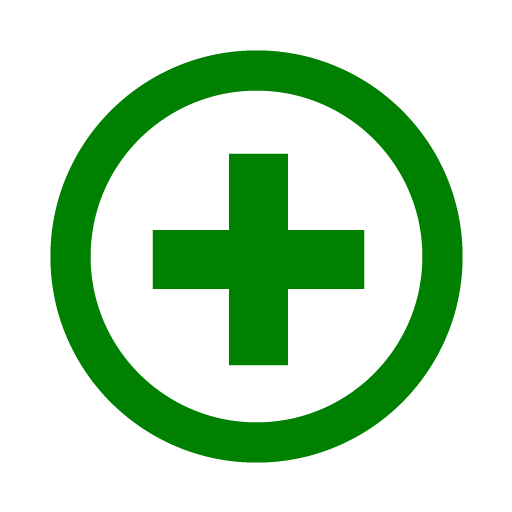** | **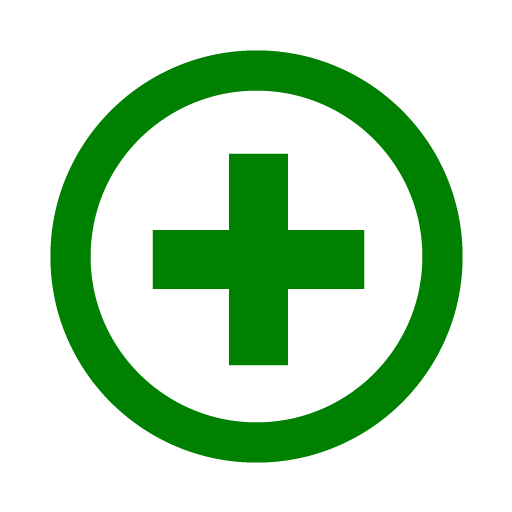** | **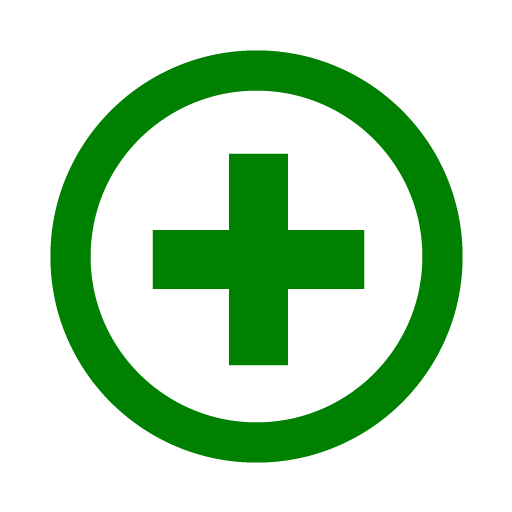** | **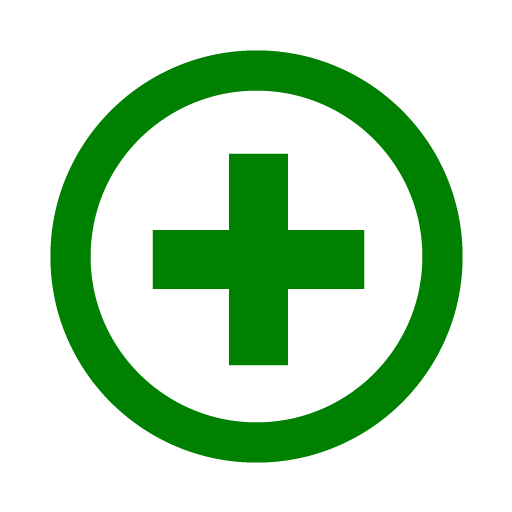** | **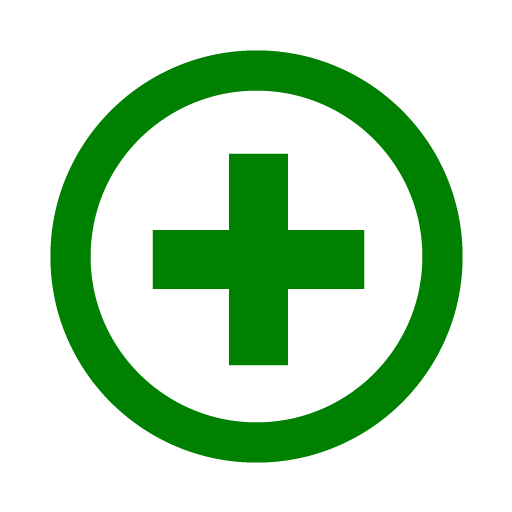** | **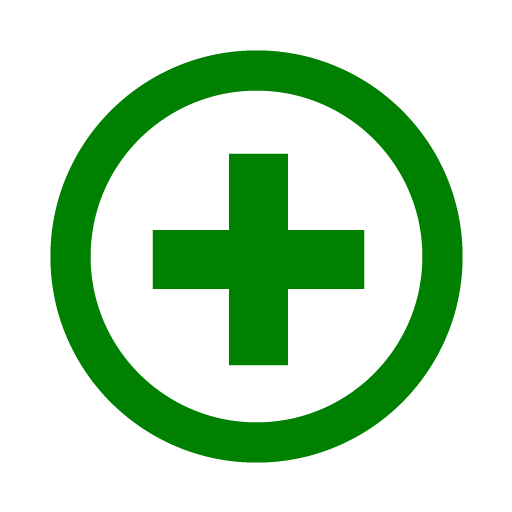** | **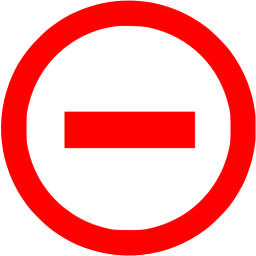** |
| Halter (2011) UK [84] | **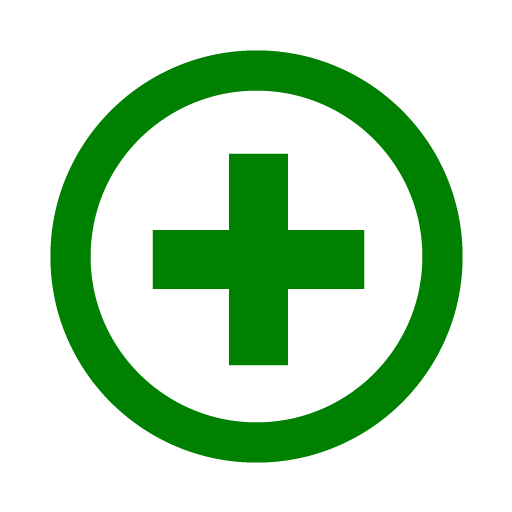** | **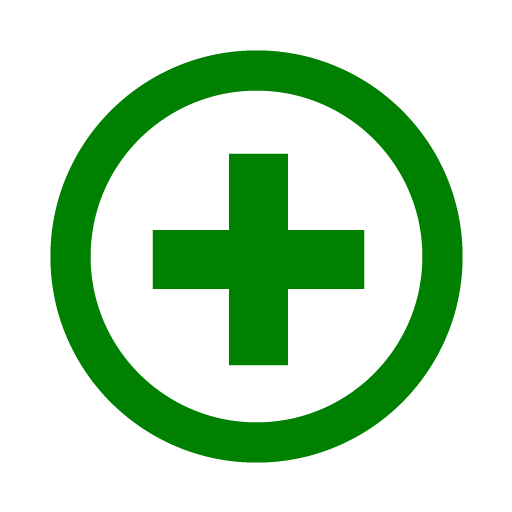** | **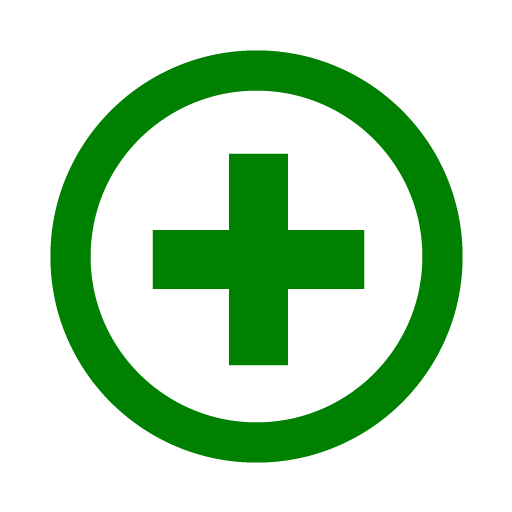** | **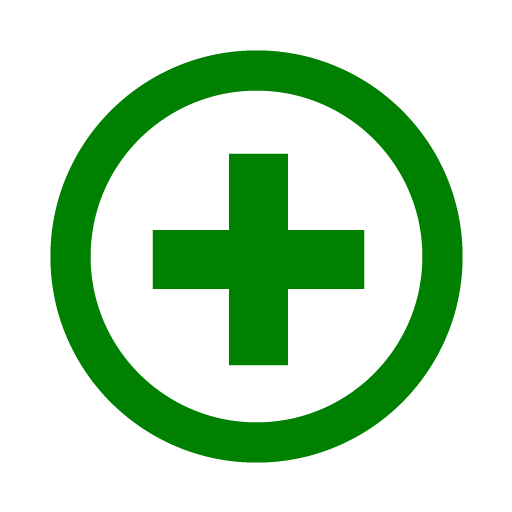** | 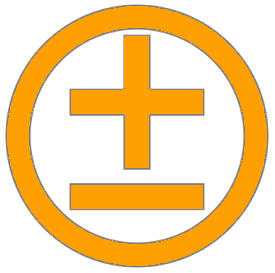 | 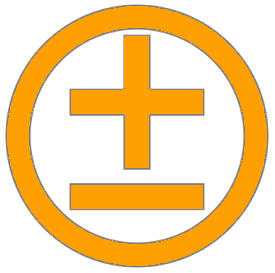 | **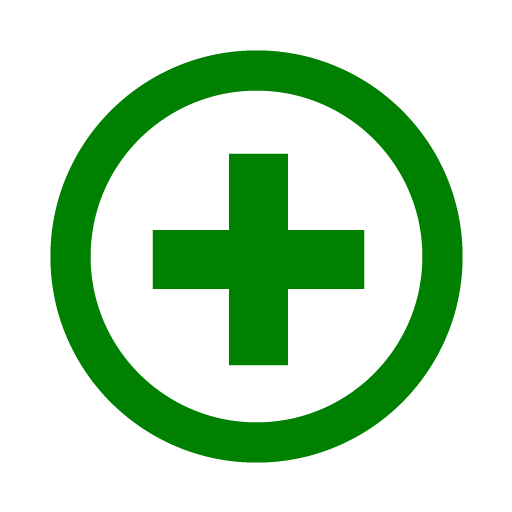** | **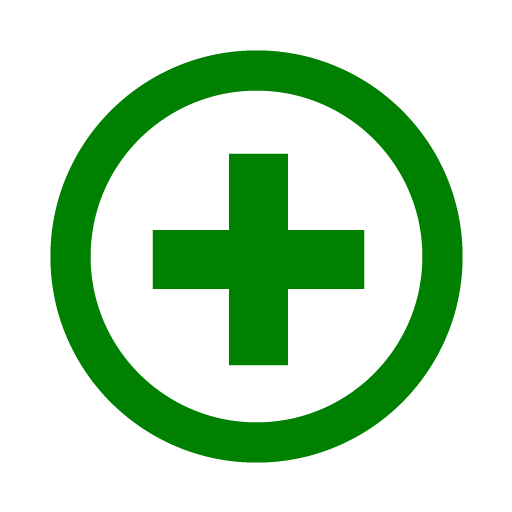** | **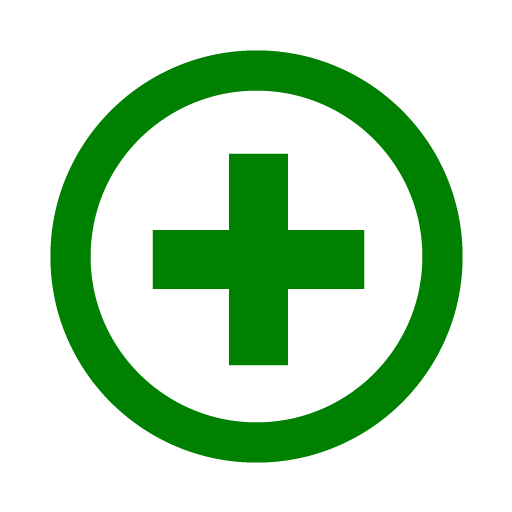** | **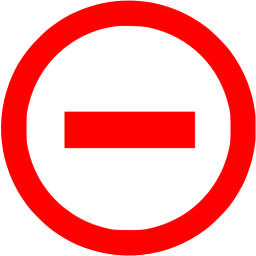** |
| Keene (2015) Australia [85] | **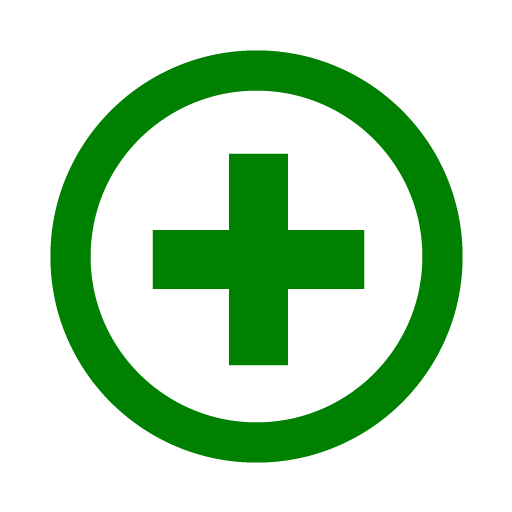** | **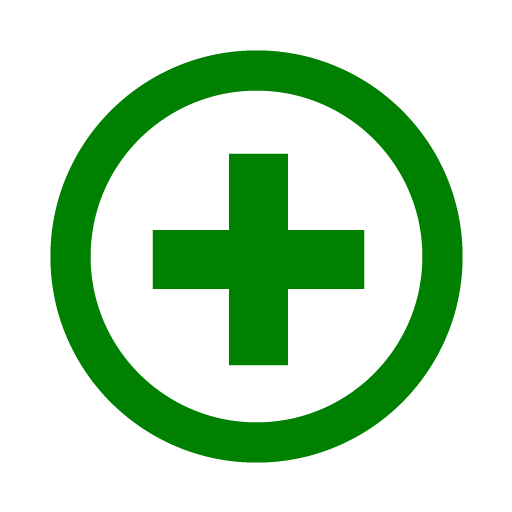** | **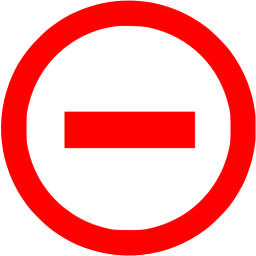** | 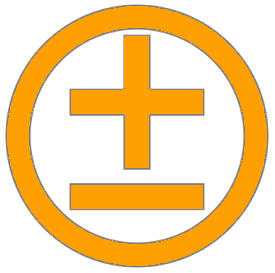 | **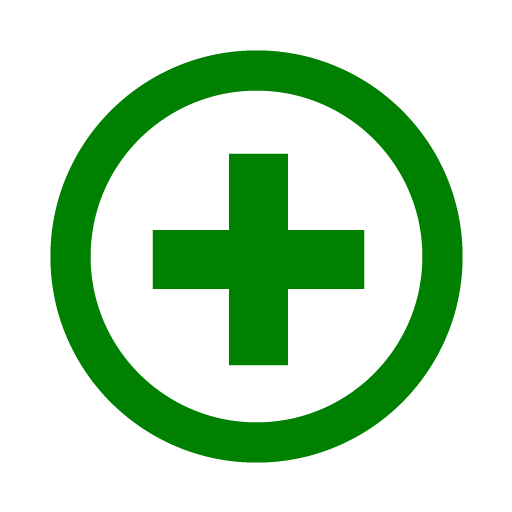** | **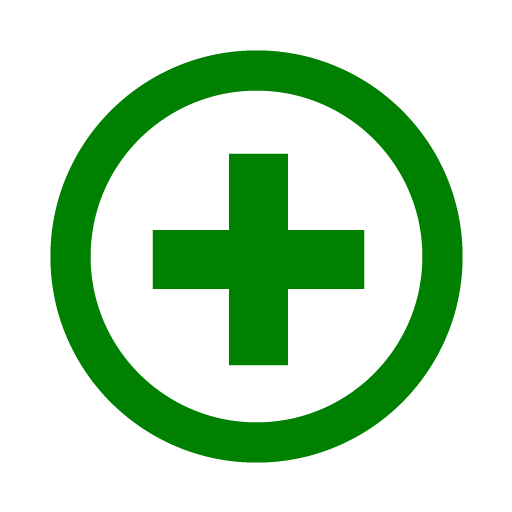** | **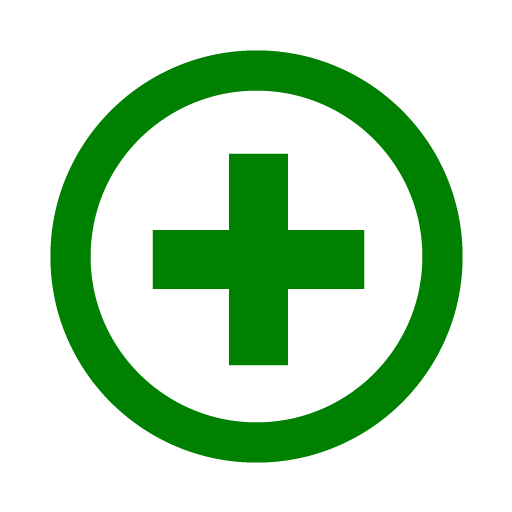** | **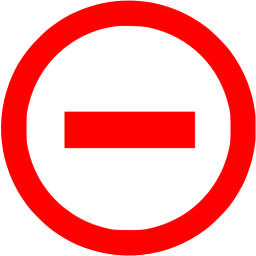** | **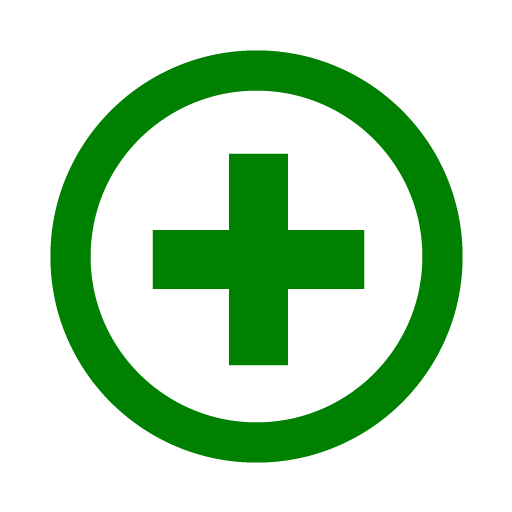** | **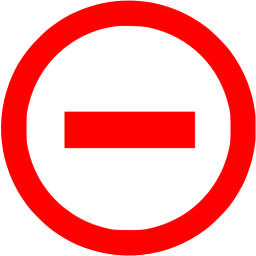** |
| Murphy-Jones (2016) UK [86] | **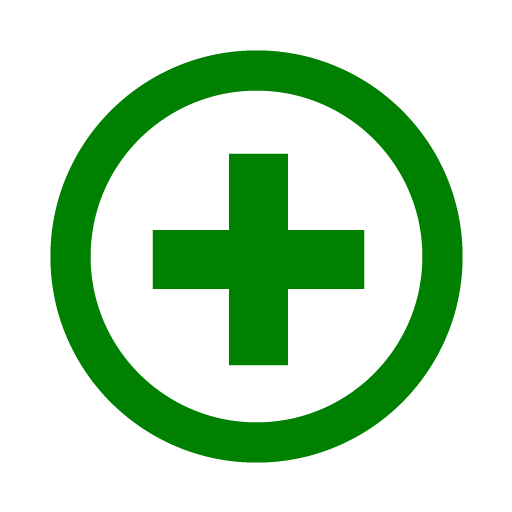** | **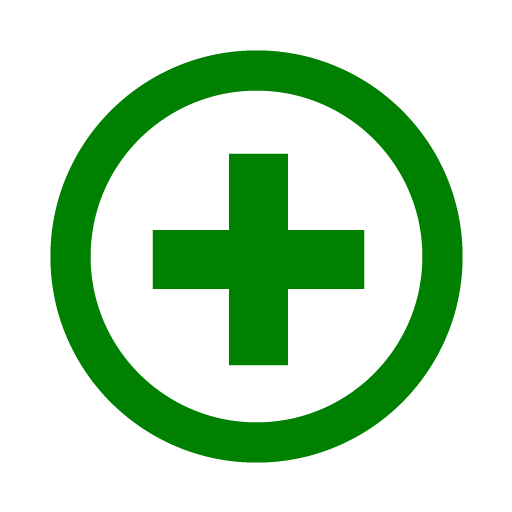** | 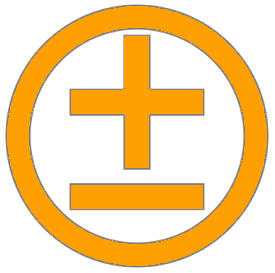 | **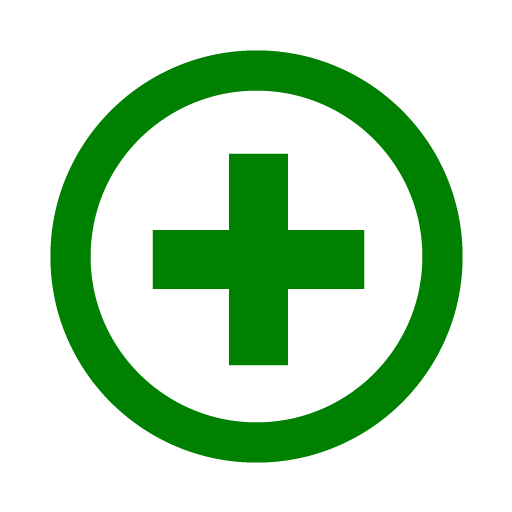** | **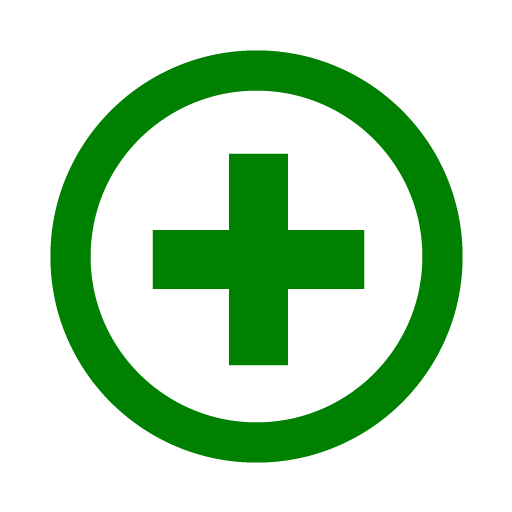** | **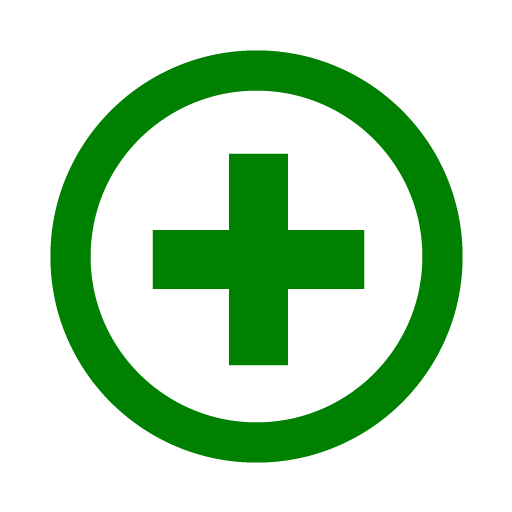** | **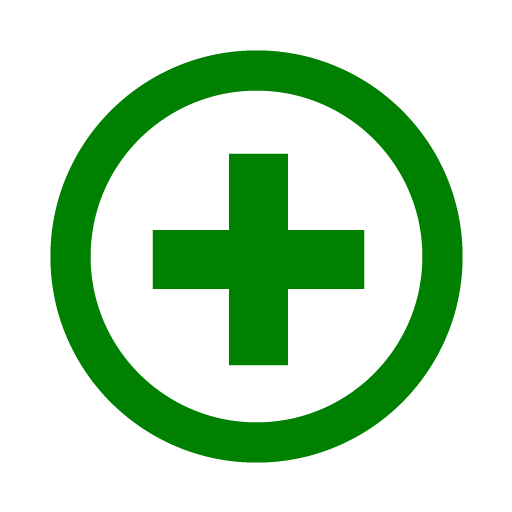** | **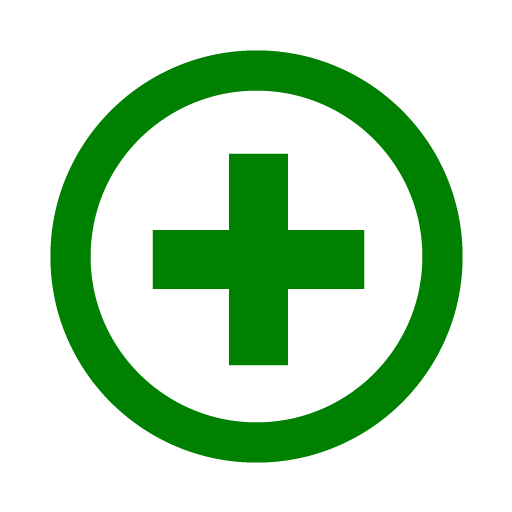** | 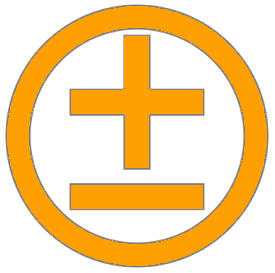 | **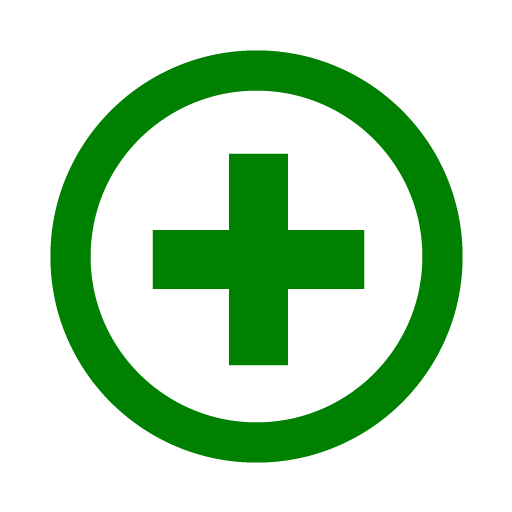** |
| O'Hara (2015) UK [87] | **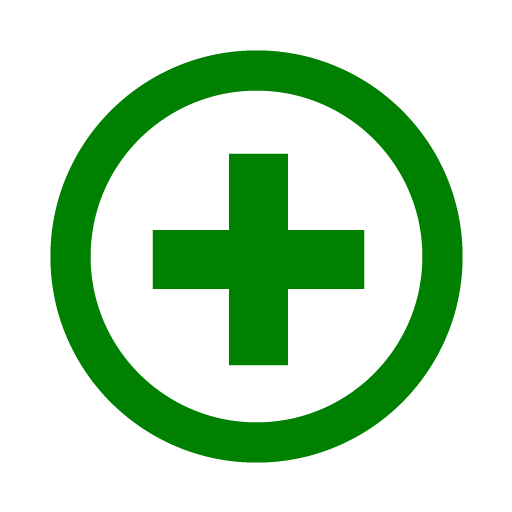** | **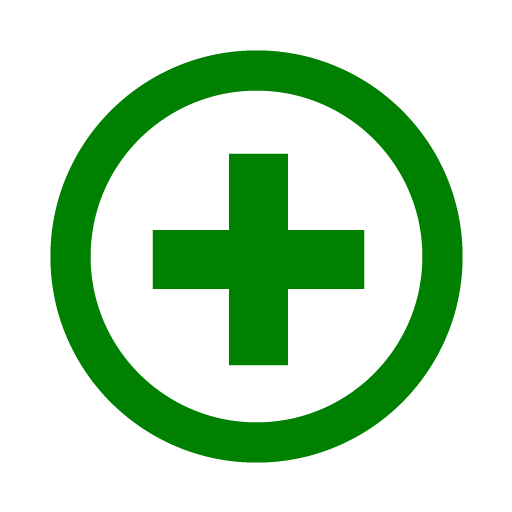** | 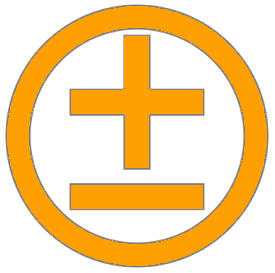 | **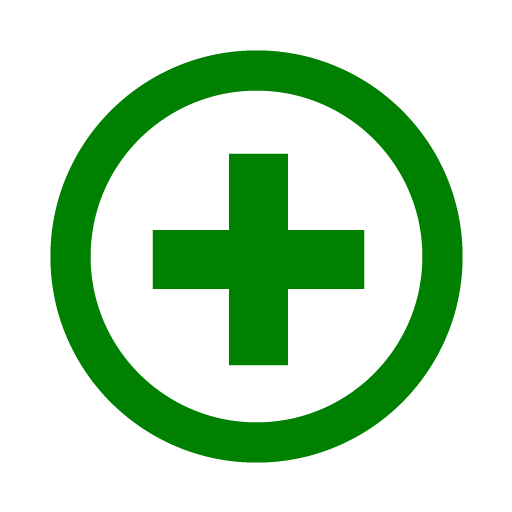** | **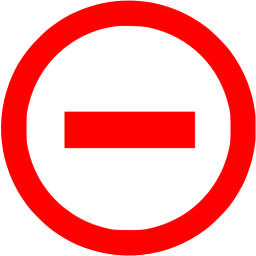** | **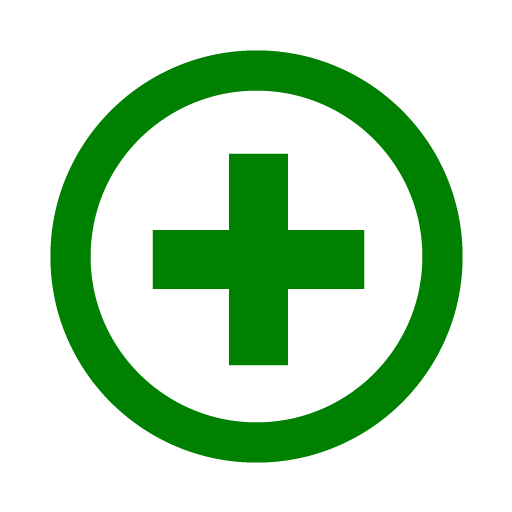** | **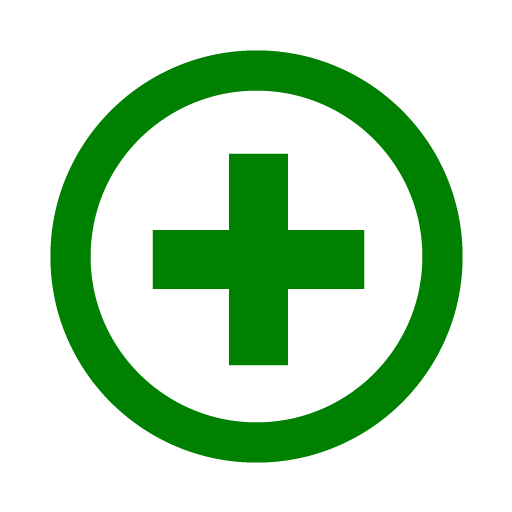** | **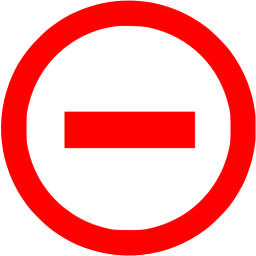** | **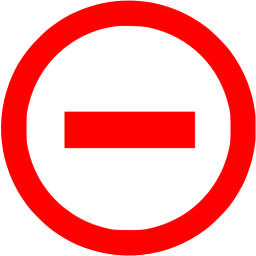** | **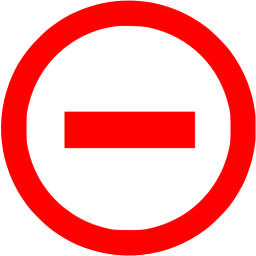** |
| Porter (2007) UK [88] | 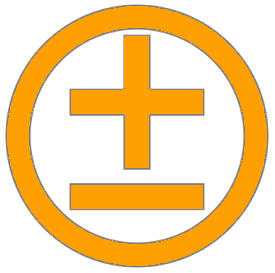 | 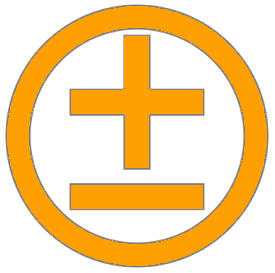 | **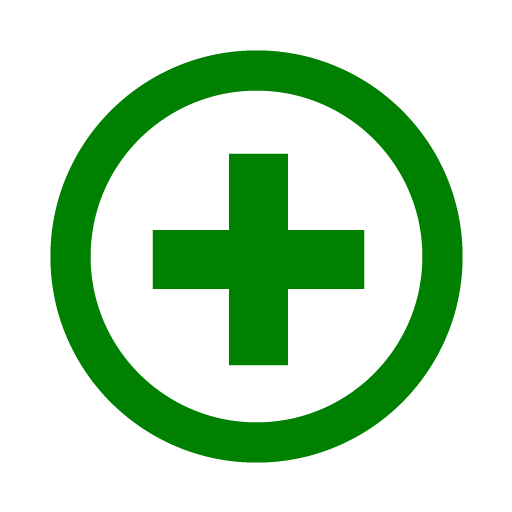** | **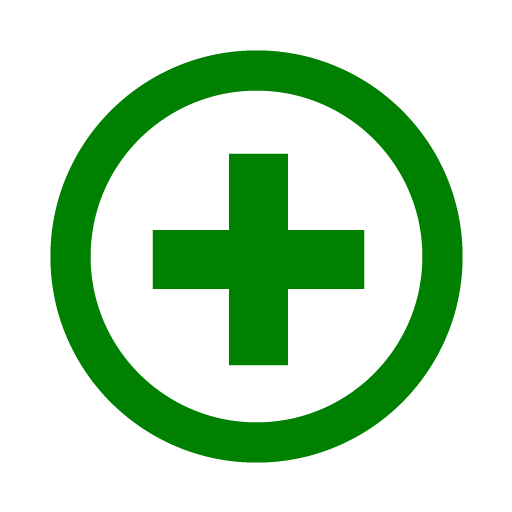** | 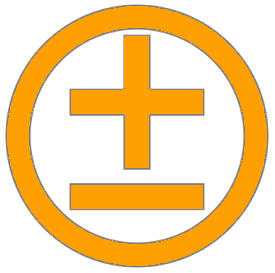 | 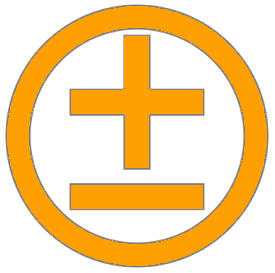 | 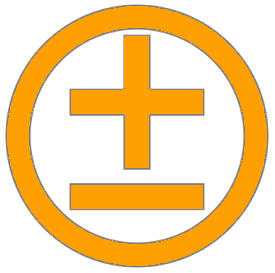 | **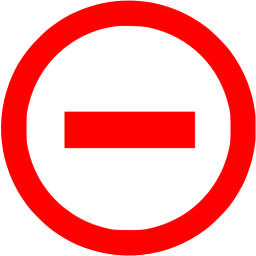** | 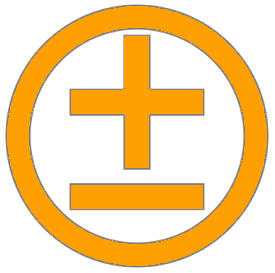 | **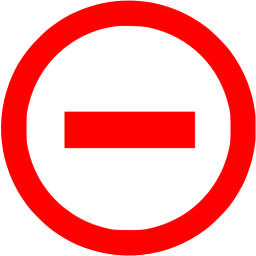** |
| Snooks (2005) UK [89] | **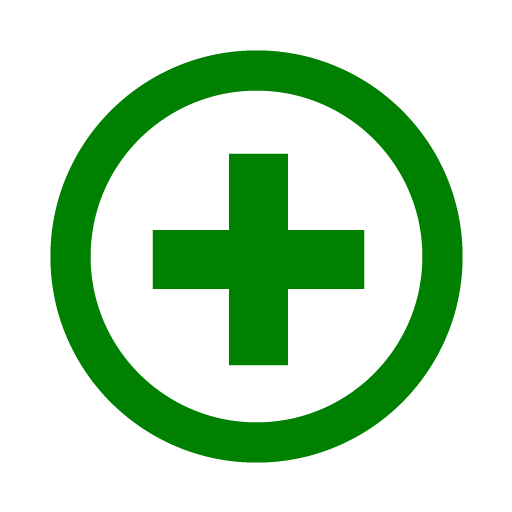** | 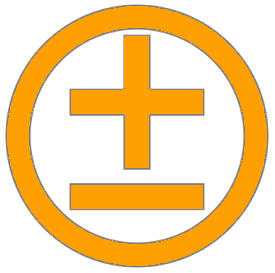 | 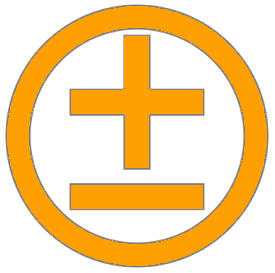 | **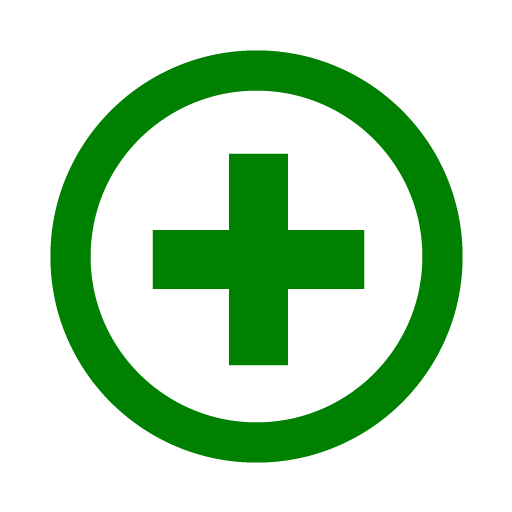** | 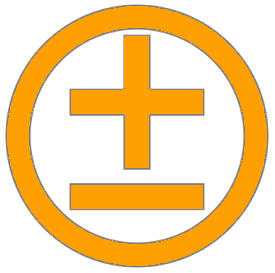 | **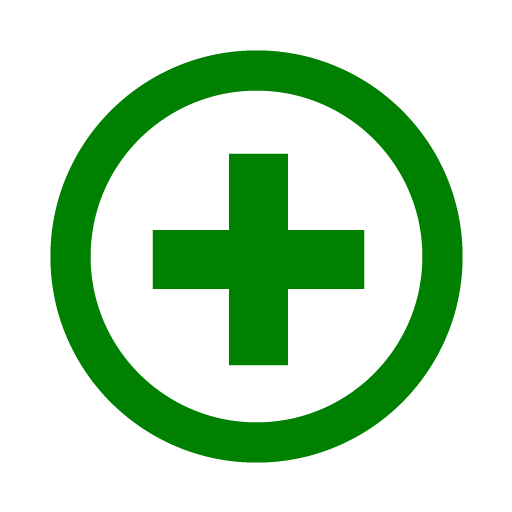** | 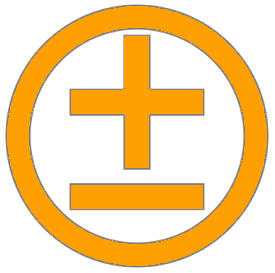 | **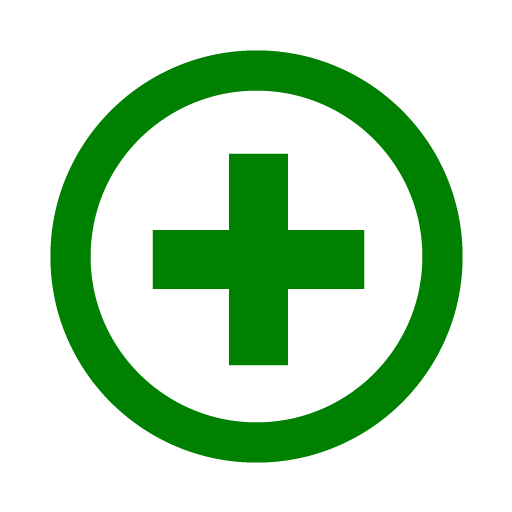** | **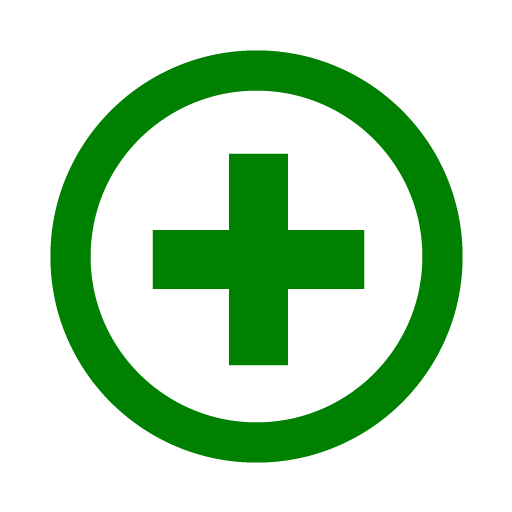** | **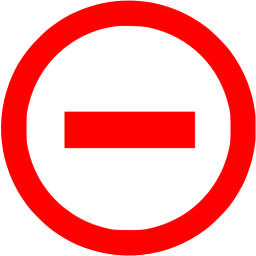** |
