# Supplementary material for: A patient-safety and professional perspective on non-conveyance in ambulance care: a systematic review
Source: Scand J Trauma Resusc Emerg Med. 2017 Jul 17;25:71. doi: 10.1186/s13049-017-0409-6 (PMC5513207; doi:10.1186/s13049-017-0409-6)
Supplement: Supplementary file 8 — Appendix 7 Non-conveyance rates and patient characteristics (DOCX 48 kb) [file 13049_2017_409_MOESM8_ESM.docx]

| **Appendix 7 - Non-conveyance rates and patient characteristics** | | | | |  |
| --- | --- | --- | --- | --- | --- |
| **1st author**  **(year)**  **Country [ref]** | **Non-conveyance rates** | **Patient category**  **(general or specific)** | **Characteristics described** | **Patient characteristics** | **Chief complaints according to ICD-10 classification (ICD-10 chapter #)** |
| **Professional and patient initiated non-conveyance** | | | | |  |
| Alrazeeni  (2016)  Saudi-Arabia [31] | 1041/7178 (14.5%) | General | Demographic – age | 71/1041 (6.8%) received treatment on-scene:   - 1-15y 4/71 (5.6%) - 15-30y 23/71 (32.4%) - 31-50y 19/71 (26.8%) - >50y 14/71 (19.7%) - NA 11/71 (15.5%)   970/1041 (93.2%) refuse by patient/relative   - 1-15y 39/970 (4.0%) - 15-30y 368/970 (38.0%) - 31-50y 204/970 (21.0%) - >50y 272/970 (28.0%) - NA 87/970 (9.0%) | - |
| Anderson  (2002)  Denmark [32] | 968/1148 (84.3%) | Specific: Hypoglycaemia | Not described | Not described | - |
| Cain  (2003)  USA [58] | 145/220 (65.9%) | Specific: Hypoglycaemia | Not described | Not described | - |
| Carter  (2002)  Canada [59] | 41/60 (68.3%) | Specific: Hypoglycaemia | Not described | Not described | - |
| Haines  (2006)  USA [62] | 704/5336 (13.2%) | Specific: children <21 years | Demographics – age  Demographics – gender  Clinical – chief complaints | Age: weighted mean 8.3 years  Gender:   - Male: 360/704 (51.1%) - Female: 344/704 (48.9%)   Medical complaint:   - Trauma 388/704 (55.1%) - Medical 309/704 (43.9%) - Other 7/704 (1.0%) | Chapter 20 - 388/704 (55.1%)  Unspecified 316/704 (44.9%) |
| Højfeld  (2014)  Denmark [34] | 1609/15392 (10.5%) | General | Clinical – chief complaints | Chief complaints/mechanism of injury:   - Factors influencing health status and contact with health services 452/1609 (28.1%)   - Observation for suspected disease or condition, unspecified 169/452 (37.4%)   - Examination and observation following transport accident 143/452 (31.6%)   - Examination and observation for other specified reasons 86/452 (19.0%)   - Examination and observation following other accident 34/452 (7.5%)   - Unspecified problem related to medical facilities and other health care 11/452 (2.4%)   - Observation for suspected myocardial infarction 4/452 (0.9%)   - Personal history of other specified conditions 3/452 (0.7%)   - Malingerer (conscious simulation) 2/452 (0.4%) - Symptoms, signs and abnormal clinical and laboratory findings, not elsewhere classified 382/1609 (23.7%) - Injury, poisoning and certain other consequences of external causes 2011/1609 (13.1%) - Endocrine, nutritional and metabolic diseases 174/1609 (10.8%) - Mental and behavioral disorders 112/1609 (7.0%) - Diseases of the respiratory system 86/1609 (5.3%) - Diseases of the circulatory system 86/1609 (5.3%) - Diseases of the nervous system 49/1609 (3.0%) - Diseases of the musculoskeletal system and connective tissue 33/1609 (2.1%) - Diseases of the digestive system 14/1609 (0.9%) - Certain infectious and parasitic diseases 8/1609 (0.5%) - Diseases of the genitourinary system 2/1609 (0.1%) | Chapter 21 - 452/1609 (28.1)  Chapter 18 - 382/1609 (23.7%)  Chapter 19 - 2011/1609 (13.1%)  Chapter 4 - 174/1609 (10.8%)  Chapter 5 - 112/1609 (7.0%)  Chapter 10 - 86/1609 (5.3%)  Chapter 9 - 86/1609 (5.3%)  Chapter 6 - 49/1609 (3.0%)  Chapter 13 - 33/1609 (2.1%)  Chapter 11 - 14/1609 (0.9%)  Chapter 1 - 8/1609 (0.5%)  Chapter 14 - 2/1609 (0.1%) |
| Jensen  (2013)  Canada [64] | 1. 21/98 (21.4%) regular ambulance crew 2. 98/140 (70.0%) extended Care Paramedics | General | Not described | Not described | - |
| Kamper  (2001)  USA [35] | 17003/53627 (31.7%) | General | Not described | Not described | - |
| Kannikeswaran  (2007)  USA [36] | 1655/5976 (27.7%) | Specific: children < 18 years | Not described | Not described | - |
| Key  (2003)  USA [30] | 1. 271/290 (93.4%) vs. 300/335 (89.6%) alertgroup 2. 3965/6463 (61.3%) vs. 2259/3372 (67.0%) MVIgroup 3. 869/1028 (84.5%) D/Cgroup | General | Not described | Not described | - |
| Mikolaizak  (2013)  Australia [26] | 11 studies: 11-56% | Specific: people >65 years who have fallen | Not described | Not described | - |
| Minhas  (2015)  Canada [39] | 76/229 (33.2%) | Specific: Supraventricular tachycardia | Demographics – age  Demographics – gender | Demographic information available for 75 patients  Age: mean 53 years  Gender:   - Male: 43/75 (57.3%) - Female 32/75 (42.7%) | - |
| Persse  (2002)  USA [69] | 1. 254/2207 (11.5%) phase 1 2. 198/1846 (10.7%) phase 2 | Specific: people >65 years | Not described | Not described | - |
| Peyravi  (2013)  Iran [41] | 1. 1999-2000 cohort: 41.8% patients received definitive on-scene treatment, 3.6% of the patients had transfer with private vehicle or refused conveyance, 2. 2011-2012 cohort: 36.2% patient received definitive on-scene treatment, 8.8% of the patients had transfer with private vehicle or refused conveyance | General | Not described | Not described | - |
| Peyravi  (2015)  Iran [42] | 3019/81999 (3.7%) | General | Not described | Not described | - |
| Pringle  (2005)  USA [43] | 906/1894 (47.8%) | General | Not described | Not described | - |
| Rudolph  (2011)  Denmark [44] | 3668/4762 (77.0%) | Specific: opioid overdose | Not described | Not described | - |
| Schmidt  (2006)  USA [45] | 191/1501 (12.7%) | General | Demographics – age  Demographics – gender  Demographics – ethnicity  Clinical – chief complaints | 1501 ambulance runs accounted for 1059 unique patients, of which 128 were not conveyed  Age: mean 46 (range 0-91)  Gender:  Male: 47/128 (36.7%)  Female: 81/128 (63.3%)  Ethnicity:  White: 115/128 (90.6%)  Non-white: 13/128 (9.4%)  Chief complaints/mechanism of injury  based on 191 runs:   - Other 55/191 (28.8%) - Trauma 52/191 (27.2%) - Neurologic 22/191 (11.5%) - Psychiatric 15/191 (7.9%) - Respiratory 12/191 (6.3%) - Endocrine 11/191 (5.8%) - Cardiovascular 7/191 (3.7%) - Rheumatic/musculoskeletal 7/191 (3.7%) - Infectious disease 5/191 (2.6%) - Gastrointestinal 3/191 (1.6%) - Skin 2/191 (1.0%) | Unspecified - 55/191 (28.8%)  Chapter 20 - 52/191 (27.2%)  Chapter 6 - 22/191 (11.5%)  Chapter 5 - 15/191 (7.9%)  Chapter 10 - 12/191 (6.3%)  Chapter 4 - 11/191 (5.8%)  Chapter 9 - 7/191 (3.7%)  Chapter 13 - 7/191 (3.7%)  Chapter 1 - 5/191 (2.6%)  Chapter 11 - 3/191 (1.6%)  Chapter 12 - 2/191 (1.0%) |
| Selden  (1990)  USA [46] | 2696/11780 (22.9%) | General | Not described | Not described | - |
| Simpson  (2014a)  Australia [74] | 419/1484 (28.2%) | Specific: people >65 years who have fallen | Demographics – age  Demographics – gender  Demographics – location  Clinical – chief complaints  Clinical – patient history | Age:   - 65-74y 77/419 (18.4%) - 75-84y 165/419 (39.4%) - ≥85y 156/419 (37.2%) - Not reported 21/419 (5.0%)   Gender:   - Male 166/419 (39.6%) - Female 249/419 (59.4%) - Not reported 4/419 (1.0%)   Chief complaints/mechanism of injury:   - Patient physiology: - Normal 274/419 (65.4%) - Abnormal 105/419 (25.1%) - Not reported 40/419 (9.5%) - New injury/pain - Yes 172/419 (41.1%) - No 243/419 (57.9%) - Not reported 4/419 (1.0%)   Patient history:   - Patient on medication, anticoagulant: - Yes 128/419 (30.5%) - No 249/419 (59.4%) - Not described 42/419 (10.0%)   Geographic area:   - Metropolitan 243/419 (58.0%) - Regional/rural 176/419 (42.0%) | - |
| Simpson  (2014b) Australia [73] | 430/1610 (26.7%) | Specific: people >65 years who have fallen | Demographics – age  Demographics – gender  Clinical – patient history | Age:   - 65-74y 78/430 (18.1%) - 75-84y 167/430 (38.8%) - ≥85y 163/430 (37.9%) - Not reported 22/430 (5.1%)   Gender:   - Male 170/430 (39.5%) - Female 256/430 (59.5%) - Not reported 4/430 (1.0%)   Patient history:   - Number of medications - 0-3: 124/430 (28.8%) - 4-7: 168/430 (39.1%) - ≥8: 97/430 (22.6%) - Missing: 41/430 (9.5%) - Patient on medication, anticoagulant: - Yes: 141/430 (32.8%) - No: 257/430 (59.8%) - Missing: 32/430 (7.4%) | - |
| Snooks  (2014)  UK [27] | 1. Intervention group: 183/436 (42.0%) 2. Control group: 126/343 (36.7%) | Specific: People who have fallen | Not described | Not described | - |
| Snooks  (2004a)  UK [28] | 1. Intervention group: 93/251 (37.1%) 2. Control group: 195/537 (36.3%) | General | Not described | Not described | - |
| Socransky  (1998)  USA [48] | 412/571 (72.2%) | Specific: hypoglycemia | Demographics – age  Demographics – gender  Clinical – patient history | Age: mean 46.8y (±17.4)  Gender:   - Male 218/412 (52.9%) - Female 194/412 (47.1%)   Patient history   - Insulin 382/412 (92.7%) - Oral hypoglycaemic 30/412 (7.3%) | - |
| Staudenmayer (2011)  USA [50] | 5865/68440 (8.6%) | Specific: injury | Demographics – age  Demographics – gender  Clinical – vital signs  Clinical – mechanism of injury | Age: mean 37.0 years (± 22.0)  Gender:   - Male 3201/5865 (54.6%) - Female 2664/5865 (45.4%)   Vital signs:   - Mean systolic blood pressure 134.7 mmHg (±21.1) - Mean hart rate 91.8 (± 15.9) - Mean GCS 15.0 (± 0.3)   Chief complaints/mechanism of injury:   - Not described 2081/5865 (35.5%) - Motor vehicle crash 1732/5865 (29.5%) - Fall 667/5865 (11.4%) - Assault and/or rape 632/5865 (10.8%) - Other 612/5865 (10.4%) - Pedestrian 93/5865 (1.6%) - Gunshot wound and/or stabbing 48/5865 (0.8%) | - |
| Tohira  (2016b)  Australia [52] | 19732/127574 (15.5%) | General | Demographics – age  Demographics – gender  Clinical – vital signs  Clinical – chief complaints | Complete data for 11096 patients  Age:   - 0-13 years: 846/11096 (7.6%) - 14-69 years: 6252/11096 (56.3%) - 70-79 years: 1303/11096 (11.7%) - ≥80 years: 2695/11096 (24.3%)   Gender:   - Male: 5306/11096 (47.8%) - Female: 5790/11096 (52.2%)   Abnormal vital signs (systolic blood pressure, oxygen saturation, glasgow coma scale, body temperature): 1658/11096 (14.9%)  Chief complaints/mechanism of injury:   - Trauma: 3167/11096 (28.5%) - Illness: 2866/11096 (25.8%) - Unknown: 1006/11096 (9.1%) - Neurological: 645/11096 (5.8%) - Intoxication: 616/11096 (5.6%) - Other: 592/11096 (5.3%) - Abdominal: 518/11096 (4.7%) - Respiratory: 508//11096 (4.6%) - Debility: 367/11096 (3.3%) - Psychosocial: 330/11096 (3.0%) - Musculoskeletal: 233/11096 (2.1%) - Infection: 98/11096 (0.9%) - Cardiology: 76/11096 (0.7%) - Urology: 74/11096 (0.7%) | Chapter 20 - 3167/11096 (28.5%)  Chapter 18 - 3233/11096 (29.1%)  Unspecified - 1598/11096 (14.4%)  Chapter 6 - 645/11096 (5.8%)  Chapter 19 - 616/11096 (5.6%)  Chapter 11 - 518/11096 (4.7%)  Chapter 10 - 508//11096 (4.6%)  Chapter 5 - 330/11096 (3.0%)  Chapter 13 - 233/11096 (2.1%)  Chapter 1 - 98/11096 (0.9%)  Chapter 9 - 76/11096 (0.7%)  Chapter 14 - 74/11096 (0.7%) |
| Van der Pols  (2011)  The Netherlands [77] | 603/1664 (36.2):   1. 275/468 (58.8%) motor ambulance 2. 328/1196 (27.4%) regular ambulance | General | Clinical – chief complaints | Chief complaints/mechanism of injury:   - Trauma 136/603 (22.6%) - Psychiatric 103/603 (17.1%) - Non-cardiac collapse 71/603 (11.8%) - Hyperventilation 67/603 (11.1%) - Gastrointestinal 56/603 (9.3%) - Convulsion 52/603 (8.6%) - Cardiology 51/603 (8.5%) - Hypoglycaemia 30/603 (5.0%) - Febrile seizure 17/603 (2.8%) - Neurology 8/603 (1.3%) - Respiratory 8/603 (1.3%) - Obstetric/gyneacology 3/603 (0.5%) | Chapter 20 - 136/603 (22.6%)  Chapter 5 - 103/603 (17.1%)  Chapter 18 - 71/603 (11.8%)  Chapter 10 - 75/603 (12.4%)  Chapter 11 - 56/603 (9.3%)  Chapter 6 - 77/603 (12.8%)  Chapter 9 - 51/603 (8.5%)  Chapter 4 - 30/603 (5.0%)  Chapter 15 - 3/603 (0.5%) |
| **Patient initiated non-conveyance** | | | | |  |
| Alicandro  (1995)  USA [29] | Not described | General | Demographic - Age  Clinical – chief complaints | Patients (n=361) who refused out of hospital medical conveyance  Age- weighted mean & ±SD (39.4 ± 21.6)  Chief complaints/mechanism of injury:   - Trauma-related 154/361 (42.7%) - Cardiorespiratory 63/361 (17.2%) - Neurologic 34/361 (9.4%) - Miscellaneous 26/361 (7.2%) - Musculoskeletal 22/361 (6.1%) - Toxicologic/environmental 20/361 (5.4%) - Gastrointestinal 16/361 (4.4%) - Obstetric/gynecologic 9/361 (2.5%) - Altered mental status 6/361 (1.7%) - Diabetes-related 6/361 (1.7%) - Psychiatric 6/361 (1.7%) | Chapter 20 - 154/361 (42.7%)  Chapters 9&10 - 63/361 (17.2%)  Chapter 6 - 40/361 (11.1%)  Unspecified - 26/361 (7.2%)  Chapter 13 - 22/361 (6.1%)  Chapter 19 - 20/361 (5.4%)  Chapter 11 - 16/361 (4.4%)  Chapter 15 - 9/361 (2.5%)  Chapter 4 - 6/361 (1.7%)  Chapter 5 - 6/361 (1.7%) |
| Burstein  (1998)  USA [57] | 69/130 (53.1%) | General | Clinical – chief complaints | Chief complaints/mechanism of injury:   - Burn/trauma 16/69 (23.2%) - Other/unknown 15/69 (21.7%) - Syncope 12/69 (17.4%) - Seizure 11/69 (15.9%) - Chest pain 8/69 (11.6%) - Respiratory distress 4/69 (5.8%) - Altered mental status 1/69 (1.4%) - Overdose/intoxication 1/69 (1.4%) - Diabetic related symptoms 1/69 (1.4%) |  |
| Chen  (1996)  Taiwan [60] | 59/1035 (5.7%) | General | Not described | Not described | - |
| Knight  (2003)  USA [37] | 26574/277221 (9.6%) | General | Not described | Not described | - |
| Moss  (1998)  USA [40] | 443/6512 (6.8%) | General | Demographics – age  Demographics – gender  Clinical – chief complaints | Age:   - <18y 93/443 (21.0%) - 18-35y 133/443 (30.0%) - 36-65y 142/443 (32.1%) - >65y 75/443 (16.9%)   Gender:   - Male: 213/443 (48.1%) - Female: 230/443 (51.9%)   Chief complaints/mechanism of injury:   - Medical chief complaints 287/443 (64.8%):   - Not-reported 108/287 (37.6%)   - Cardiac chest pain 66/287 (23.0%)   - Respiratory distress/shortness of breath 21/287 (7.3%)   - Seizure 20/287 (7.0%)   - Other-medical 72/287 (25.1%) - Trauma 156/443 (35.2%)   - 150/156 (96.2%) had a GCS of 15 | Unspecified 108/443 (40.6%)  Chapter 9 - 66/443 (14.9%)  Chapter 10 - 21/443 (4.7%)  Chapter 6 - 20/443 (4.5%)  Chapter 20 - 156/443 (35.2%) |
| Shaw  (2006)  UK [81] | 12018/76635 (15.7%) | General | Not described | Not described | - |
| Stark  (1990)  USA [49] | 89/1715 (5.2%) | General | Not described | Not described | - |
| Stuhlmiller  (2005)  USA [51] | 119/137 (86.9%) | General | Not described | Not described | - |
| Vilke  (1999)  USA [54] | 5702/94466 (6.0%) | Specific: heroin overdose | Demographics – age  Dempgraphics – gender | Data available for a subgroup of patients (n=317) who received naloxone and were released on-scene against medical advice  Age: mean 37.0 years, range 18-79 years  Gender:   - Male: 262/317 (82.7%) - Female: 55/317 (17.3%) | - |
| **Professional initiated non-conveyance** | | | | |  |
| Deasy  (2008)  UK [61] | 81/263 (30.8%) | General | Clinical – chief complaints | Chief complaints/mechanism of injury:   - Not reported 35/81 (43.2%) - Minor road traffic accident 20/81 (24.7%) - Wounds 8/81 (9.9%) - Seizures 7/81 (8.6%) - Alcohol intoxication 7/81 (8.6%) - Vasovagal event 4/81 (4.9%) | Unspecified - 35/81 (43.2%)  Chapter 20 - 20/81 (24.7%)  Chapter 19 - 15/81 (18.5%)  Chapter 6 - 7/81 (8.6%)  Chapter 18 - 4/81 (4.9%) |
| Gerlacher  (2001)  USA [79] | 3057/15409 (19.8%) | Specific: children < 12 years | Demographics – age  Dempgraphics – gender  Demographics – ethnicity  Clinical – chief complaints | Age   - <2 years 948/3057 (31.0%) - 2-6 years 1126/3057 (36.8%) - 7-12 years 983/3057 (32.2%)   Gender   - Male 1637/3057 (53.5%) - Female 1420/3057 (46.5%)   Etnicity   - Afro-american 1478/3057 (48.3%) - Hispanic 927/3057 (30.4%) - Caucasian 516/3057 (16.9%) - Other 107/3057 (3.5%)   Chief complaints/mechanism of injury:   - Injury 849/3057 (27.8%) - Motor vehicle accident 623/3057 (20.4%) - Choking episode 311/3057 (10.2%) - Respiratory problems 211/3057 (6.9%) - Other 199/3057 (6.5%) - Febrile illness 188/3057 (6.1%) - Poisoning 112/3057 (3.7%) - Vomiting/diarrhea 102/3057 (3.3%) - Pain 81/3057 (2.6%) - Nosebleed 62/3057 (2.0%) - Bite (human or animal) 62/3057 (2.0%) - Foreign body 53/3057 (1.7%) - Auto-pedestrian accident 47/3057 (1.5%) - Seizure 45/3057 (1.5%) - Locked in a car 44/3057 (1.4%) - Crying 39/3057 (1.3%) - Cut ring off finger 29/3057 (0.9%) | Chapter 19 - 1014/3057 (33.2%)  Chapter 20 - 732/3057 (23.9%)  Chapter 10 - 584/3057 (19.1%)  Unspecified - 199/3057 (6.5%)  Chapter 18 - 269/3057 (8.8%)  Chapter 11 - 102/3057 (3.3%)  Chapter 6 - 45/3057 (1.5%) |
| Magnusson  (2016)  Sweden [38] | 200/529 (37.8%) | General | Demographics – age  Demographics – gender  Clinical – chief complaints | Age: 53.4 years (±26 years), median 54.5  Gender:   - Male: 88/200 (44.0%) - Female: 112 (56.0%)  \| Chief complaints/mechanism of injury:   - Medical 109/200 (54.5%)   - Unspecific condition, proneness to fall 18/109 (16.5%)   - Infection, fever 13/109 (11.9%)   - Vertigo, headache 28/109 (25.7%)   - Chest pain, arrhythmia 19/109 (17.8%0   - Miscellaneous 31/109 (28.4%) - Surgical 41/200 (21.5%)   - Abdominal pain 21/41 (51.2%)   - Trauma, thorax/head 11/41 (26.8%)   - Miscellaneous 9/41 (22.0%) - Orthopaedic 25/200 (12.5%)   - Trauma extremity 11/25 (44.0%)   - Back pain 11/25 (44.0%)   - Miscellaneous 3/25 (12.0%) - Psychiatric 25/200 (12.5%)   - Anxiety, psychotic behavior 8/25 (32.0%)   - Social deterioration, alcohol 11/25 (44.0%)   - Miscellaneous 6/25 (24.0%) \| \| --- \| | Chapter 20 – 22/200 (11.0%)  Chapter 1 – 13/200 (6.5%)  Chapter 18 - 88/200 (44.0%)  Chapter 9 – 19/200 (9.5%)  Chapter 5 – 19/200 (9.5%)  Chapter 6 – 28/200 (14.0%)  Chapter 13 – 11/200 (5.5%) |
| Strote  (2008)  USA [75] | 402/2359 (17.0%) | Specific: hypoglycaemia | Not described | Not described | - |
| Tohira  (2016a)  Australia [53] | 1. 88/629 (14.0%) post-ictal patients 2. 74/609 (12.2%) hypoglycaemic patients | Specific: hypoclycaemia, post-ictal | Not described | Not described | - |
| **Initiation unknown/not described** | | | | |  |
| Burstein  (1996)  USA [56] | Not described | General | Demographics – age  Clinical – chief complaints | Patients not conveyed (n=321)  Average age: 39 years (range 0-94)  Chief complaints/mechanism of injury:   - Trauma 139/321 (43.3%) - Cardiac/respiratory 52/321 (16.2%) - Miscellaneous 29/321 (9.0%) - Neurological 28/321 (8.7%) - Musculoskeletal 23/321 (7.2%) - Intoxication (incl. ethanol) 15/321 (4.7%) - Gastrointestinal 12/321 (3.7%) - Obstetric/gynecological 7/321 (2.2%) - Diabetic-related 6/321 (1.9%) - Altered mental status 5/321 (1.6%) - Psychiatric 5/321 (1.6%) | Chapter 20 - 139/321 (43.3%)  Chapters 9&10 - 52/321 (16.2%)  Chapter 18 -29/321 (9.0%)  Chapter 6 - 33/321 (10.3%)  Chapter 13 - 23/321 (7.2%)  Chapter 19 - 15/321 (4.7%)  Chapter 11 - 12/321 (3.7%)  Chapter 14 - 7/321 (2.2%)  Chapter 4 - 6/321 (1.9%)  Chapter 5 - 5/321 (1.6%) |
| Goldstein  (2015)  Canada [33] | 1. 11489/63076 (18.2%) ≥16 years 2. 3693/63076 (5.9%) ≥65 years 3. 7796/63076 (12.4%) 16-64 years | General | Demographics – age  Demographics – gender  Demographics – location | Demographics of patients ≥65 years are described  Age: mean 78.32±8.51  Gender:   - Male: 1666/3693 (45.1%) - Female: 2027/3693 (54.9%)   Location   - Rural 701/3693 (19.0%) - Urban/not indicated 2992/3693 (81.0%) | - |
| Hipskind  (1997)  USA [63] | Not described | General | Demographics – age  Demographics – gender  Clinical – chief complaints  Clinical – vital signs  Clinical – patient history | Patients (n=683) who refused conveyance:  Age:   - 0-10 years 129/683 (18.9%) - 11-40 years 388/683 (56.8%) - 41-60 years 93/683 (13.6%) - >60 years 73/683 (10.7%)   Gender:   - Male 350/683 (51.2%) - Female 333/683 (48.8%)   Chief complaints/mechanism of injury:   - Autocrash 286/683 (41.9%) - Other trauma 182/683 (26.6%) - Medical 136/683 (19.9%) - Other 40/683 (5.8%) - Psychiatric 14/683 (2.0%) - Cardiac 11/683 (1.6%) - Seizure 7/683 (1.0%) - Hypoglycemia 6/683 (0.9%) - Not registered 1/683 (0.1%)   Vital signs:   - Blood pressure: - Within normal limits 478/683 (70.0%) - Not registered 156/683 (22.8%) - Diastolic blood pressure >100 mmHg 38/683 (5.6%) - Systolic blood pressure <100 mmHg 9/683 (1.3%) - Pulse rate: - Within normal limits 493/683 (72.2%) - Not registered 104/683 (15.2%) - >100 bpm 82/683 (12.0%) - <60 bpm 4/683 (0.5%) - Respiratory rate: - Within normal limits 432/683 (63.2%) - >18 pm 134/683 (19.6%) - Not registered 116/683 (17.0%) - <12 pm 1/683 (0.1%)   Patient history:   - None 469/683 (68.7%) - Other 59/683 (8.6%) - Not registered 40/683 (5.9%) - Cardiac 26/683 (3.8%) - Hypertension 26/683 (3.8%) - Diabetes 22/683 (3.2%) - Seizure disorder 13/683 (1.9%) - Psychiatric 11/683 (1.6%) - Alcohol abuse 6/683 (0.9%) - COPD 5/683 (0.7%) - CVA 4/683 (0.6%) - Cancer 1/683 (0.1%) - Drug abuse 1/683 (0.1%) | Chapter 20 – 468/683 (68.5%)  Unspecified – 177/683 (25.9%)  Chapter 5 - 14/683 (2.0%)  Chapter 9 - 11/683 (1.6%)  Chapter 6 - 7/683 (1.0%)  Chapter 4 - 6/683 (0.9%) |
| Kahalé  (2006)  Canada [65] | Not described | Specific: children <16 years | Demographics – age  Demographics – gender  Demographics – location  Clinical – chief complaints | Age: mean 6.0 years (±5.1 year) range (0–15 year)  Gender:   - Male 201/345 (58.3%) - Female 144/345 (41.7%)   Location of patient pick-up:   - Residence 202/345 (58.6%) - Street/highway/road 75/345 (21.7%) - Public place 36/345 (10.4%) - Other 21/345 (6.1%) - School 6/345 (1.7%) - Unobtainable 5/345 (1.5%)   Chief complaints/mechanism of injury:   - Trauma 175/345 (50.7%) - Medical 156/345 (45.2%):   - Respiratory 54/156 (34.6%)     - Shortness of breath 19/54 (35.2%)     - Choking–resolved 17/54 (31.5%)     - Asthma 8/54 (14.8%)     - Hyperventilation 6/54 (11.1%)     - Cough 4/54 (7.4%)   - Gastrointestinal/vomiting 17/156 (10.9%)   - Neurologic/seizure 14/156 (9.0%)   - Syncope 13/156 (8.3%)   - Fever 10/156 (6.4%)   - Abdominal pain 7/156 (4.5%)   - Drug overdose 7/156 (4.5%)   - Nosebleed 5/156 (3.2%)   - Rash 4/156 (2.6%)   - Other 25/156 (16.0%) - Unobtainable 14/345 (4.1%) | Chapter 20 - 175/345 (50.7%)  Chapter 10 - 59/345 (17.1%)  Unspecified – 39/345 (11.3%)  Chapter 11 - 17/345 (4.9%)  Chapter 6 - 14/345 (4.1%)  Chapter 18 - 20/345 (5.88%)  Chapter 1 - 10/345 (2.9%)  Chapter 19 - 7/345 (2.0%)  Chapter 12 - 4/345 (1.2%) |
| Keene  (2015)  Australia [85] | Not described | General | Demographics – age  Demographics – gender  Clinical – chief complaints | Patients (n=20) not conveyed  Age: mean 68.5 years, range 24-91 years, median 77 years, IQR 60-86 years  Gender:   - Male 10/20 (50.0%) - Female 10/20 (50.0%)   Chief complaints/mechanism of injury:   - Fall 4/20 (20.0%) - Laceration 3/20 (15.0%) - Motor vehicle crash 2/20 (10.0%) - Soft tissue injury 2/20 (10.0%) - Febrile illness 2/20 (10.0%) - Apparent syncope 2/20 (10.0%) - Post seizure 1/20 (5%) - Vomiting 1/20 (5%) - Chest infection 1/20 (5%) - Chest pain 1/20 (5%) - Hypoglycaemia 1/20 (5%) | Chapter 20 - 6/20 (30.0%)  Chapter 19 – 5/20 (25.0%)  Chapter 1 – 1/20 (5.0%)  Chapter 18 - 4/20 (20.0%)  Chapter 6 - 1/20 (5%)  Chapter 11 - 1/20 (5%)  Chapters 9&10 - 1/20 (5%)  Chapter 4 - 1/20 (5%) |
| Lerner  (2003)  USA [66] | Not described | Specific: hypoglycaemia | Demographics – age  Demographics – gender | Hypoglycemic patients (n=36) not conveyed  Age: mean 47 years (±19 years)  Gender:   - Male 19/36 (53.0%) - Female 17/36 (47.0%) | - |
| Marks  (2002)  UK [9] | Not described | General | Demographics – age  Demographics – gender  Clinical – chief complaints | Patients (n=498) not conveyed  Age distribution shown in figure 1, but precise numbers cannot be described. Distribution shows peak in the ages of 75 to 90 years.  Gender:   - Male: 229/498 (46.0%) - Female: 269/498 (54.0%)   Chief complaints/mechanism of injury:   - Falls 170/498 (34.1%) - Non-specific illness 51/498 (10.2%) - Road traffic accident 39/498 (7.8%) - Psychiatric (non self-harm) 30/498 (6.0%) - Insufficient to categorize 30/498 (6.0%) - Intoxication 30/498 (6.0%) - Diabetes 26/498 (5.2%) - General assistance 24/498 (4.8%) - Other accident/injury 22/498 (4.4%) - Epilepsy 15/498 (3.0%) - Self-harm (including overdose) 14/498 (2.8%) - Respiratory 10/498 (2.0%) - Assault 10/498 (2.0%) - Musculoskeletal 10/498 (2.0%) - Gastrointestinal 7/498 (1.4%) - Cardiac 4/498 (0.8%) - Obstetrics and gynaecology 4/498 (0.8%) - Neurological 1/498 (0.2%) - Not reported 1/498 (0.2%) | Chapter 20 – 233/498 (46.8%)  Chapter 18 - 51/498 (10.2%)  Chapter 5 - 30/498 (6.0%)  Unspecified - 31/498 (6.2%)  Chapter 4 - 26/498 (5.2%)  Chapter 21 - 24/498 (4.8%)  Chapter 19 - 52/498 (10.4)  Chapter 6 - 16/498 (3.2%)  Chapter 10 - 10/498 (2.0%)  Chapter 13 - 10/498 (2.0%)  Chapter 11 - 7/498 (1.4%)  Chapter 9 - 4/498 (0.8%)  Chapter 14 - 4/498 (0.8%) |
| Mechem  (1998)  USA [67] | Not described | Specific: hypoglycaemia | Demographics – age  Demographics – gender | Patients (n=103) with hypoglycaemia  Age:   - Male: mean age 54y (±3) - Female: mean age 60y (±3)   Gender:   - Male 56/103 (54.4%) - Female 47/103 (45.6%) | - |
| Newton  (2015)  South Africa [68] | 144/1121 (12.8%) | General | Not described | Not described | - |
| Tiedemann  (2013)  UK [76] | Not described | Specific: people ≥70 years who have fallen | Demographics – age  Demographics – gender  Clinical – past medical history  Clinical – patient history | Patients (n=251) who have fallen and were not conveyed  Age: mean±SD 82.6 years (±5.9 years)  Gender:   - Male 110/251 (43.8%) - Female 141/251 (56.2%)   Patient history:   - Urinary incontinence: 110/251 (43.8%) - Central nervous system disorder: 98/251 (39.0%) - Painful feet: 88/251 (35.1%) - Diabetes mellitus: 58/251 (23.1%) - Macular degeneration: 46/251 (18.3%) - Pain interfered “extremely’ with activities in the past 4 weeks: 26/251 (10.4%) - >8 medications: 129/251 (51.4%) - Takes any central nervous system medications: 117/251 (46.6%) | - |
| Vilke  (2002)  USA [78] | Not described | Specific: patients ≥65 years signed out against medical advice | Demographics – age  Clinical – chief complaints | Patients (n=100) who signed out against medical advice  Age: mean±SD 72.2years ±6.4 years  Chief complaints/mechanism of injury:   - Trauma: 41/100 (41.0%)   - Motor vehicle crash: 18/41 (43.9%%)   - Fall: 17/41 (41.5%)   - Assault: 3/41 (7.3%)   - Burn: 2/41 (4.9%)   - Bicycle crash: 1/41 (2.3%) - Neurological: 29/100 (29.0%)   - Loss of consciousness: 16/29 (55.2%)   - Seizure: 8/29 (27.6%%)   - Migraine: 2/29 (6.9%%)   - Head pain: 2/29 (6.9%)   - Autonomic dysregulation: 1/29 (3.4%) - Other: 15/100 (15.0%)   - Abdominal pain: 4/15 (26.7%)   - Bleeding: 2/15 (13.3%)   - Leg pain: 2/15 (13.3%)   - Nausea: 2/15 (13.3%)   - Anaphylaxis: 1/15 (6.7%)   - Flank pain: 1/15 (6.7%)   - Heartburn: 1/15 (6.7%)   - Medication refill: 1/15 (6.7%)   - Shoulder pain: 1/15 (6.7%) - Cardiovascular: 9/100 (9.0%)   - Chest pain: 7/9 (77.8%)   - Pulselessness: 2/9 (22.2%) - Respiratory: 6/100 (6.0%)   - Shortness of breath: 4/6 (66.7%)   - Hyperventilation: 2/6 (33.3%) | Chapter 20 - 41/100 (41.0%)  Chapter 6 - 29/100 (29.0%)  Unspecified - 15/100 (15.0%)  Chapter 9 - 9/100 (9.0%)  Chapter 10 - 6/100 (6.0%) |

*Patient initiated can also be relative or next of kin initiated
