# Supplementary material for: A patient-safety and professional perspective on non-conveyance in ambulance care: a systematic review
Source: Scand J Trauma Resusc Emerg Med. 2017 Jul 17;25:71. doi: 10.1186/s13049-017-0409-6 (PMC5513207; doi:10.1186/s13049-017-0409-6)
Supplement: Supplementary file 9 — Appendix 8 Guidelines/protocols/triage criteria (DOCX 29 kb) [file 13049_2017_409_MOESM9_ESM.docx]

| **Appendix 8 - Guidelines/protocols/triage criteria** | |
| --- | --- |
| **1st author**  **(year)**  **Country [ref]** | **Guidelines/protocols/triage criteria** |
| Alicandro  (1995)  USA [29] | High risk criteria to identify patients at high risk for an adverse event if not conveyed:   1. Disorientation to person or place or time or situation (yes/no) 2. Altered level of consciousness (yes/no) 3. Head injury (yes/no/suspected) 4. Alcohol/drug ingestion by exam or history (yes/no/suspected) 5. Age < 17 years (yes/no) 6. Pulse >110 or <50 bpm (yes/no) 7. Systolic blood pressure >200mmHg or <90mmHg (yes/no) 8. Respiration rate >30 or <12 pm (yes/no) 9. Serious chief complaints, such as chest pain, dyspnea, syncope (yes/no)   If any of these criteria scores ‘yes’ then the EMS professional should make special efforts to convince the patient to go to the hospital for further evaluation |
| Carter  (2002)  Canada [59] | Treat-and-release criteria for hypoglycemic patients:   1. Normal Glasgow Coma Scale (GCS) 2. A responsible adult on-scene |
| Gerlacher  (2001)  USA [79] | Non-transport criterion for pediatric patients:   1. The decision must be agreed upon by the consulting doctor at On Line Medical Control |
| Halter  (2011)  UK [84] | Assessment and referral tool for elderly patients who have fallen:   1. Head injury or any alteration in level of consciousness (yes/no) 2. Concerns with respiratory system:    1. Change in respiratory pattern (yes/no)    2. Respiratory distress (yes/no) 3. Concerns with other observations:    1. Systemic upset (yes/no)    2. Cardiac chest pain (yes/no) 4. Worrying injury (yes/no) 5. Change in level of function with impact on ability to remain at home safely (yes/no) 6. Patient asks to be conveyed (yes/no)   If any of these criteria scores ‘yes’ then the patient should be conveyed to the ED. In case of non-conveyance (all criteria score ‘no’), then inform GP. |
| Minhas  (2015)  Canada [39] | Treat-and-release criteria for patients with supraventricular tachycardia:   1. The patient is between 18 and 65 years of age 2. The patient has a history of recurrent SVT known to be responsive to adenosine or vagal manoeuvres, without previous recurrence of SVT shortly following treatment 3. The patient does not have any concurrent acute illness 4. The patient has remained asymptomatic and hemodynamically stable for at least 15 minutes post-conversion 5. The patient has not experienced an episode of SVT requiring treatment in the past 24 h 6. The patient agrees and is comfortable with the decision not to be transported to the hospital at this time 7. The patient has the means to immediately call 9-1-1 if symptoms recur, and EMS advises the patient to do this 8. There is a responsible adult who will remain with the patient for at least 4 h 9. EMS has answered all patient questions about their care 10. The patient has signed the release waiver on the SVT information sheet 11. The patient understands and agrees to follow EMS recommendations for follow-up care 12. The SVT treat-and-refer patient information sheet was left with someone at the scene |
| Moss  (1998)  USA [40] | Criteria for patients who want to sign out against medical advice: 1. Is patient oriented to person, place, date? 2. Is patient impaired by drugs or alcohol? 3. Is patient competent to refuse care? 4. Have risks and consequences of refusal been discussed? 5. Has patient been advised that medics will return if called back? 6. Has AMA form been signed? |
| Porter  (2007)  UK [88] | Non-conveyance criteria for patients:   1. The patient refused to travel 2. The patient met the criteria for non-conveyance in the hypoglycaemia treat and refer protocol (criteria not specified) 3. The patient was dead and met the criteria for non-resuscitation |
| Pringle  (2005)  USA [43] | Criteria for non-conveyance:   1. Cardio-respiratory    1. Shortness of breath    2. Chest pain any age    3. Diastolic blood pressure (BP) of 110 or above with symptoms or >120 without symptoms 2. Abdominal pain 3. Overdose or suspected overdose 4. Neurological    1. Unconsciousness    2. Seizures    3. Acute neurological deficit 5. Pregnancy    1. With a seizure or history of seizures    2. BP of 140/90 or greater    3. History of ruptured membranes    4. Any unusual vaginal bleeding or discharge associated with    5. pregnancy    6. Any premature labor before 9th month of pregnancy    7. Trauma during pregnancy 6. Age >70 or <12 years who does not clearly meet nontransport criteria or is unable to effectively communicate or express his/her symptoms 7. Motor vehicle collision 8. Alcohol: breath alcohol content >.3 with symptoms of lethargy or coma 9. Carbon monoxide exposure   If any of these criteria scores ‘yes’ then the patient should be conveyed to the ED. |
| Schmidt  (2000; 2001)  USA [70;72] | Transport criteria for patients:   1. Unconsciousness or changed mental status 2. Respiratory distress 3. Sustained abnormal vital signs*    1. Systolic blood pressure less than 90 mm Hg    2. Pulse less than 60 or more than 110 beats/min    3. Respiratory rate less than 12 or more than 24 breaths/min 4. Comprised airway 5. Uncontrolled bleeding 6. Suspected abuse of any type 7. Suspected cervical spinal injury 8. Infants less than 3 months of age   If any of these criteria scores ‘yes’ then the patient should be conveyed to the ED. |
| Selden  (1990)  USA [46] | Criteria for Release:   1. History 2. Normal vital signs 3. Physical examination appropriate for complaint 4. Mental status assessment (alert and oriented) 5. No significant impairment due to drugs, alcohol, other organic cause, or mental illness 6. Risks of refusing care or transport explained and understood by patient |
| Simpson  (2014b)  Australia [73] | Treat-and-release criteria for people who have fallen:   1. Are high risk criteria present?    1. Positive red emergency response criteria    2. Positive yellow clinical review criteria    3. Major trauma criteria    4. Suspected medical cause    5. Injury requiring treatment in ED    6. Patient on regular anti-coagulant medication 2. Is the patient injured? 3. Are mobility assessment criteria met?    1. Can the patient stand from sitting    2. without paramedic assistance?    3. Can the patient weight bear?    4. Is the patient able to mobilize (i.e. toileting, for food and drink)?    5. Is the patients level of function normal for them? 4. Is the professional concerned that the patient may have another fall <48 hours? |
| Snooks  (2004a)  UK [28] | Treat-and-refer protocols were developed on 23 subject, no complete protocols described:   1. Minor allergic reactions 2. Insect bites and stings 3. Boils/abscesses 4. Splinter removal 5. Postoperative wound problem 6. Dressing problem 7. Wounds (minor) 8. Soft tissue injuries (minor) 9. Epistaxis 10. Sore throat 11. Cold or flu symptoms 12. Toothache 13. Fit (= seizure) in known epileptic 14. Resolved hypoglycaemia in known diabetic (insulin dependent) 15. Back pain 16. Diarrhea 17. Constipation 18. Blocked urinary catheter 19. Emotional or hysterical reaction 20. Alcohol intoxication 21. Social problems 22. Faints 23. Falls |
| Strote  (2008)  USA [75] | Treat-and-release criteria for hypoglycemic patients:   1. respond to the glucose and are fully alert with a posttreatment glucose of at least 60 mg/dL 2. able to eat 3. someone present for at least 6 hours of observation   Patients receive written instructions from the paramedic:   1. to call the GP before the next insulin dose or reduce insulin dose with 25% if GP cannot be contacted 2. to check blood glucose frequently for the next several hours 3. in case of worsening symptoms: call EMS |
| Stuhlmiller  (2005)  USA [51] | Transport criteria worksheet with 9 criteria for patient assessment:   1. Oriented to person, place, time, and situation (yes/no) 2. Altered level of consciousness (yes/no) 3. Head injury (yes/no) 4. Alcohol or drug ingestion by exam or history (yes/no) 5. Nature of complaint:    1. Chest pain (yes/no)    2. Shortness of breath (yes/no)    3. Other (yes/no) 6. Pulse rate <50 or >100 bpm (yes/no) 7. Systolic blood pressure <100mmHg or >200mmHg (yes/no) 8. Diastolic blood pressure <50mmHg or >100mmHg (yes/no) 9. Respiratory rate <12 or >24 per minute (yes/no)   If any of these criteria scores ‘yes’ then the patient should be conveyed to the ED. |
| Tohira  (2016a)  Australia [53] | Checklists for 2 patient groups are reported:  Post-ictal patients:   1. Age >16 year old 2. Known epileptic 3. Normal seizure duration 4. No evidence of the patient feeling unwell prior to the seizure 5. Not pregnant 6. No history of a recent head injury 7. Not intoxicated 8. No recurrent fits during the past week 9. No significant injury resulting from the fit 10. Apyrexic 11. There is a competent care giver   Patients with hypoglycemia:   1. Age >16 years old 2. Able to consume oral carbohydrates 3. Blood sugar level is increasing since the administration of glucagon and/or oral carbohydrates 4. Known diabetic 5. Score on the Glasgow Coma Scale = 15 6. Blood sugar level >5.0 mmol/L 7. Not intoxicated 8. No history of recurrent episodes of hypoglycemia during the past week 9. No significant injury resulting from hypoglycemia 10. No irritability or altered behavior   For both patient groups all items must be met to be discharged at the scene |
| Tohira  (2016b)  Australia [52] | Paramedics were allowed to discharge a patient when, in their clinical judgement, they felt it was unnecessary to transport the patient to ED. |
| Vilke  (1999)  USA [54] | Release against medical advice criteria for patients with a suspected heroin overdose:   1. Is the patient oriented? (yes/no) 2. Is the patient not impaired by drugs or alcohol? (yes/no) 3. Is the patient competent to refuse care? (yes/no) 4. Have risks and consequences been discussed? (yes/no) 5. Has the patient been advised that medics will return if called back? (yes/no) 6. Has the AMA form been signed? (yes/no)   If all of these criteria scores ‘yes’, and responses favorably to naloxone 2mg IV/IM/4mg via endotracheal tube, and want to leave, then the patient can be signed out against medical advice |
